# A multicenter clinical study on parent-implemented early intervention for children with global developmental delay

**DOI:** 10.3389/fped.2023.1052665

**Published:** 2023-02-15

**Authors:** Ping Dong, Qiong Xu, Ying Zhang, Dong-yun Li, Bing-rui Zhou, Chun-chun Hu, Chun-xue Liu, Xin-rui Tang, Shi-yun Fu, Lan Zhang, Hai-feng Li, Fei-yong Jia, Xiu-bin Tong, Jie Wang, Hui-ping Li, Xiu Xu

**Affiliations:** ^1^ Department of Child Healthcare, Children’s Hospital of Fudan University, National Children’s Medical Center, Shanghai, China; ^2^ Department of Child Healthcare, Chengdu Women’s and Children’s Central Hospital, Chengdu, China; ^3^ Department of Rehabilitation, Children’s Hospital of Zhejiang University School of Medicine, Hangzhou, China; ^4^ Department of Developmental-Behavioral Pediatrics, The First Hospital of Jilin University, Changchun, China; ^5^ Department of Child Healthcare, Children’s Hospital of Fudan University at Xiamen, Xiamen, China; ^6^ Department of Child Healthcare, Shanghai Maternal and Child Health Hospital of Changning District, Shanghai, China

**Keywords:** global developmental delay, children, parent-implemented early intervention program, parenting stress, multicenter study

## Abstract

**Objective:**

Early identification and intervention for children with global developmental delay (GDD) can significantly improve their prognosis and reduce the possibility of developing intellectual disability in the future. This study aimed to explore the clinical effectiveness of a parent-implemented early intervention program (PIEIP) for GDD, providing a research basis for the extended application of this intervention strategy in the future.

**Methods:**

During the period between September 2019 and August 2020, children aged 3 to 6 months diagnosed with GDD were selected from each research center as the experimental group and the control group. For the experimental group, the PIEIP intervention was conducted for the parent-child pair. Mid-term and end-stage assessments were performed, respectively, at 12 and 24 months of age, and parenting stress surveys were completed.

**Results:**

The average age of the enrolled children was 4.56 ± 1.08 months for the experimental group (*n* = 153) and 4.50 ± 1.04 months for the control group (*n* = 153). The comparative analysis of the variation in the progress between the two groups by independent *t*-test showed that, after the experimental intervention, the developmental quotient (DQ) of locomotor, personal-social, and language, as well as the general quotient (GQ) of the Griffiths Mental Development Scale-Chinese (GDS-C), the children in the experimental group demonstrated higher progress than those in the control group (*P* < 0.05). Furthermore, there was a significant decrease in the mean standard score of dysfunctional interaction, difficult children and the total level of parental stress in the term test for the experimental groups (*P* < 0.001 for all).

**Conclusions:**

PIEIP intervention can significantly improve the developmental outcome and prognosis of children with GDD, especially in the areas of locomotor, personal-social, and language.

## Introduction

Global developmental delay (GDD) refers to a delay in two or more domains of development in children under the age of five years, including activities of daily living, personal social skills, motor skills, and cognitive and language/speech development (1). The developmental outcome of GDD has many possibilities. Timeous treatment and intervention for GDD can restore some of the children's measured intelligence quotient (IQ) when they reach the age of the feasible intelligence test and mitigate the onset of severe intellectual disability (ID). And those who do not intervene in a timely manner or are seriously retarded may develop into ID. The global prevalence of GDD is approximately between 1% and 3% (2, 3). Furthermore, GDD is often caused by genetic factors (i.e., chromosomes, genes, metabolic diseases, etc.), perinatal factors (i.e., congenital infection, toxic exposure, birth injury, asphyxia, premature delivery, intracranial hemorrhage, etc.), social and cultural factors (i.e., isolation, lack of stimulation, and loss of learning opportunities), and early diseases (i.e., severe concussion, traumatic brain injury, malnutrition, poisoning, and endocrine diseases). Thus, GDD has a complex etiology and a high disability rate. Moreover, no effective cure has been discovered, which exacerbates the mental and economic burden imposed on the family and society, severely affecting the quality of the population.

The first three years of life are considered a critical period for infant brain development. During this period, neurogenesis, myelination, and synaptogenesis interact. Repeated exposure of infants to multiple types of stimulation during early growth enhances the activation of the underlying circuits in the brain and forms new neural connections. Therefore, this period is also considered important for developing the sensitivity of the infant's brain to stimulation. Scientific interventions that target this critical period of brain development are crucial for improving neural development and reducing disabilities.

Several studies have shown that parental or caregiver involvement in interventions has a positive effect on functional improvement in children with neurodevelopmental disorders (NDDs) such as autism spectrum disorder (ASD) and attention deficit hyperactivity disorder (ADHD) (4–9). Furthermore, a few studies have examined family interventions for cerebral palsy (10–12), impaired vision (13), and school-age ID (14). But so far, including very young children in the first two years of life is relatively few, and the most commonly reported disability of child participants included was ASD (15). To date, only a few studies have investigated family interventions for children with GDD in the early postnatal period (i.e., the first three years). A previous clinical study showed that early intervention and the addition of a structured home activity program (HAP) demonstrated a positive effect on brain development in children with GDD (16). Furthermore, in 2018, several scholars published an article on the influence of parental involvement on the occupational treatment of children with GDD (17). Overall, the sample size of the two studies was small, and the initial age of intervention was relatively older (i.e., the average age of these participants at enrollment was 20.7 and 46.8 months, respectively).

Currently, there is increasing advocacy for interventions aimed at children with various types of NDDs in natural scenes (18). Particularly, families comprise the natural scenes within which children are most exposed. Hence, the active participation of parents in early intervention activities is crucial. Moreover, they play the dual roles of parents and educational trainers in the intervention of children with GDD during the first three years after birth when the brain plasticity is most strong. Thus, we designed and developed a parent-implemented early intervention program (PIEIP). Providing training for caregivers of children with GDD to accurately understand and implement family-appropriate interventions is a key feature of the PIEIP.

In addition, parents with various NDDs experience increased parenting stress in contrast to parents with children with typical development (TD) (19). However, limited studies underscore psychosocial mediators that influence parental intervention on child development outcomes. Family interventions are associated not only with parenting stress but also with developmental outcomes in children with GDD. Therefore, parenting stress is considered one of the mediators of both family interventions and developmental outcomes in children with GDD.

By conducting this multicenter pretest-posttest experimental study, we aimed to assess the effect of the PIEIP intervention for children with GDD compared to the control group based on child outcomes at 24 months. Furthermore, the target group that is likely to gain the most from early intervention needs to be chosen considering the extensive amount of time and money required for early intervention. Thus, the second aim of this study was to investigate the possible predictors of PIEIP efficacy. Possible predictors included paternal variables (i.e., parents' age, educational level, parenting stress, and family income) and child variables (i.e., age of children at enrollment, sex, gestational age, birth weight, and developmental level at baseline).

## Participants and methods

### Ethical approval

This study was approved by the Ethics Committee of the Children's Hospital of Fudan University [Children's Hospital of Fudan University Ethics Protocol (2016) no.131]. This study follows the Declaration of Helsinki and informed consent was obtained from the guardian of the child with GDD.

### Participants

The inclusion criteria of children with GDD were based on: (1) a chronological age ranging from 3 to 6 months (corrected age for use in premature infants); (2) the assessment of a physician and therapists where children with significant delays in two or more areas of development were diagnosed with GDD (Griffiths Mental Development Scale-Chinese (GDS-C) assessed developmental quotient (DQ) < 70); and (3) the knowledge and consent of the guardians.

The exclusion criteria comprised: (1) children diagnosed with a genetic disorder, congenital deformity, physical disability, audiovisual disability, traumatic brain injury, neurodegenerative diseases, or neuro-musculoskeletal disorders; and (2) children with other severe chronic diseases.

**Figure 1:**
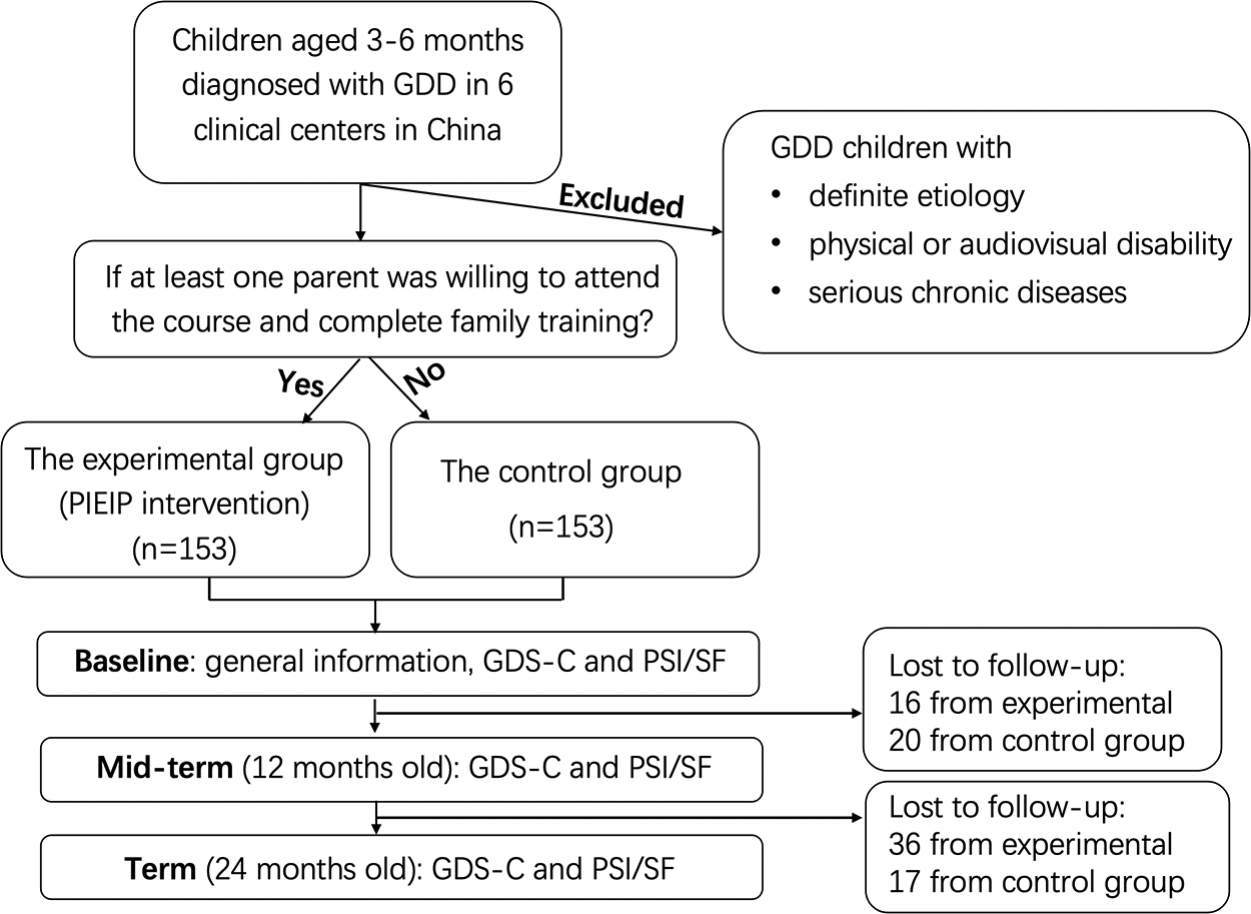
Recruitment and follow-up flow chart. GD, global developmental delay; PIEIP, parent-implemented early intervention program; GDS-C, Griffiths Mental Development Scale-Chinese; PSI/SF, parental stress index-short form.

According to the voluntary principle of parents, children with GDD were assigned to the experimental group or the control group. For the experimental group, at least one parent was willing to attend the course and complete family training. The recruitment and follow-up flowchart is shown in Figure 1.

### Research design and process

**Figure 2:**
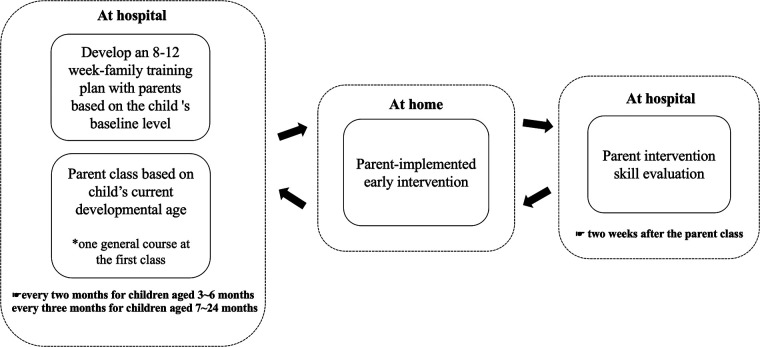
The process framework of parent-implemented early intervention program (PIEIP) intervention.

The research design for the experimental (PIEIP intervention) group involved: (1) a baseline assessment based on basic demographic data, medical history, GDS-C and parental stress index-short form (PSI/SF); (2) Several processes followed after enrollment including (1) performing a baseline ability assessment and drafting the first family intervention training plan; (2) participation of parents in the parent class at the hospital corresponding with the current development level of children; (3) begin training in the family environment; (4) return of parents to the hospital for parent intervention skill evaluation after two weeks of parent class; (5) return home and continue training; (6) regularly (i.e., once every two months for children aged 3–6 months old and once every three months for 7–24 month-year-olds) come back to the hospital to reassess the ability of children and write the next stage of the family training plan. Furthermore, parents were required to participate in the subsequent stages of the parent class and continue family training repeatedly until the child reached 24 months of age. We summarize the process framework of PIEIP intervention with Figure 2. All the children could be enrolled in any additional institutional therapy.

Furthermore, with regard to our PIEIP: (1) Family intervention training plan formulation: we used our specific assessment scales and tools to assess the current ability of children to write 8–12-week family intervention training programs. The evaluation scale included seven aspects, namely, understanding communication, expressive communication, social interaction, play skills, fine motor skills, gross motor skills, and self-care ability. Each developmental domain had to write 3–4 training plans, namely, (1) to align development with children's current skills, (2) each training objective ought to be specific and measurable, and (3) deliverables expected to be achieved within subsequent 8–12 weeks. (2) Parent classes comprised: (1) one general course introducing the basic law of neuromotor development in children and some basic intervention-related technologies such as applied behavior analysis (ABA), types and utilization of assistance, establishing the training environment, maintaining the face-to-face position, and managing children's problematic behavior during training; (2) eight parent courses for different age stages (i.e., 3–4 months, 5–6 months, 7–9 months, 10–12 months, 13–15 months, 16–18 months, 19–21 months and 22–24 months) to introduce the developmental level of each developmental domain at this age stage. Furthermore, the parents are coached on how to encourage the development of children's corresponding skills through a variety of item games or sensory social games in the family environment. Moreover, this course includes other suggestions about integrating training goals into daily care. The teaching form first constituted theoretical teaching before transforming into a demonstration and discussion, comprising a total of 90 min. (3) Parent intervention skill evaluation: parents were required to submit two 10 to 15-minute home training videos two weeks after the parent class and rate the video clips according to a fidelity table and the guidance feedback for parents.

The control group: (1) Baseline assessment: basic demographic data, medical history, GDS-C; and PSI/SF; (2) After enrollment: parents were free to participate in any institutional-based rehabilitation, telephone follow-up occurred every three months, where parents were asked about the basic milestone level of children and providing child health care guidance until 24 months of age.

### Outcome measurement

Mid-term and final assessments were conducted in the two groups at the ages of 12 months and 24 months. The content included the GDS-C and PSI/SF.

1. GDS-C: Child cognitive development before and after the intervention was evaluated (20, 21). The Griffiths Mental Development Scale (GMDS) was originally developed by Ruth Griffiths in the United Kingdom in 1954, which is a widely used diagnostic measure in many countries and has good psychometric properties (22). And the Chinese version of the GMDS, namely GDS-C, which was used in our current study has been adapted to assess the development of Chinese children after completing the revision of China norm research in seven cities between 2009 and 2013. It displays good reliability and validity (21). To manage children in a laboratory setting, physicians assessed different aspects of mental development in infants and children through semi-structured activities. The five subscales that were administered and scored for children under two ages included locomotor [A] (assessing a child's gross motor skills including his or her balance and ability to coordinate movements), personal-social [B] (assessing a child's self-care ability and the ability of interact with other children, language [C] (assessing a child's receptive language and expressive language ability), eye-hand coordination [D] (assessing a child's fine motor skills, finger dexterity, and visual tracking, and performance [E] (assessing a child's visual spatial ability, including processing speed and accuracy). According to the Chinese norm, the raw sub-scale scores were converted into percentiles and developmental age equivalents. The DQ = developmental age/chronological age × 100. The general quotient (GQ) was derived by calculating the average of the raw scores of the five subscales. All scores were standardized (M = 100, SD = 15).
2. PSI/SF: PSI/SF is widely used clinically as a standardized tool to identify stress early in parent-child relationships (23). The PSI/SF contains 36 terms rated on a five-point Likert scale. Based on the three scales (Parental Distress (PD), Parent-Child Dysfunctional Interaction (P-CDI), and Difficult Child (DC)), the PSI/SF yielded a total stress score. Considering only the individual factors of parents as caregivers, PD was used to define caregiver stress levels. During their interactions with their children, parents perceived P-CDI as a difficult and problematic situation. Specifically, the feeling of rejection by their children comprised a form of P-CDI. Moreover, DC examined parents' perceptions of a grumpy child in the household and the child's behavioral characteristics.

### Statistical analysis

SPSS software [version 26; SPSS, Inc., Chicago, IL, United States] was used to perform statistical analyses. Descriptive analysis, independent *t*-test, *χ*^2^ test, paired-sample *t*-test, and multivariate linear regression analysis were used in this study. The significance level for all statistical methods was set at *P* < 0.05.

## Results

### Children's demographic characteristics and baseline development level

A total of 306 children were recruited for this study according to the criteria. The participants' characteristics are shown in Table 1. There were no significant differences in chronological age (*t* = 0.535, *P* = 0.593), sex (*χ*^2 ^= 1.096, *P* = 0.295), birth weight (Z = −0.685, *P* = 0.493), delivery mode (*χ*^2 ^= 0.511, *P* = 0.475), parents' education level (*χ*^2 ^= 0.052, *P* = 0.819), family income (*χ*^2 ^= 0.327, *P* = 0.567), and parents' age (*χ*^2 ^= 0.678, *P* = 0.410) between the experimental and control groups. Table 1 shows a significant difference in gestational age (Z = −2.143, *P* = 0.032).

**Table 1:** Baseline characteristics of infants with GDD.

Characteristics of children	All cases (*n* = 306)	Intervention group (*n* = 153)	Control group (*n* = 153)	X^2^/t/Z-value	*P*-value
Age at enrollment [mo, mean(SD)]	4.53 (1.06)	4.56 (1.08)	4.50 (1.04)	0.535	0.593
Male [n (%)]	181 (59.2%)	86 (56.2%)	95 (62.1%)	1.096	0.295
Gestational age [wk, M(P25, P75)]	38.0 (37.0, 39.0)	37.6 (37.0, 38.15)	38.0 (37.0, 39.0)	−2.143	0.032*
Birth weight [g, M (P25, P75)]	2670.00 (2437.50, 3150.00)	2660.00 (2430.00, 3127.50))	2680.00 (2445.00, 3264.00)	−0.685	0.493
Cesarean section [n (%)]	196 (64.1%)	101 (66.0%)	95 (62.1%)	0.511	0.475
**Parents’ education level [n (%)]**					
<12 years of education	154 (50.3%)	76 (49.7%)	78 (51.0%)	0.052	0.819
>12 years of education	152 (49.7%)	77 (50.3%)	75 (49.0%)		
**Family income**					
<20,000 USD	147 (48.0%)	76 (49.7%)	71 (46.4%)	0.327	0.567
>20,000 USD	159 (52.0%)	77 (50.3%)	82 (53.6%)		
**Parents’ age**					
30 and under	189 (61.8%)	98 (64.1%)	91 (59.5%)	0.678	0.410
31 and over	117 (38.2%)	55 (35.9%)	62 (40.5%)		

GDD, global developmental delay; SD, standard deviation; USD, United States dollar.

* *P*-value <0.05.

During the initial assessment, the average age of the enrolled children was 4.53 ± 1.06 [standard deviation (SD)] months. Based on the GDS-C, the pretest developmental age was 2.09 ± 1.04 months for locomotor, 2.28 ± 1.12 months for personal-social, 2.30 ± 1.13 months for language, 2.52 ± 1.29 months for eye-hand coordination and 2.72 ± 1.30 months for performance. The mean deferred times for these children were 2.44 months for locomotor, 2.25 months for personal-social, 2.23 months for language, 2.01 months for eye-hand coordination, and 1.81 months for performance. Furthermore, there was no difference in the pretest developmental age-equivalent between groups in each development domain (*P* > 0.05, for all analyses) (Table 2).

**Table 2:** The developmental age-equivalent at baseline of GDD children (GDS-C).

Groups	n	Locomotor (A)	Personal-Social (B)	Language (C)	Eye-hand coordination (D)	Performance (E)
Intervention group	153	2.04 (1.04)	2.21 (1.13)	2.19 (1.13)	2.45 (1.30)	2.65 (1.29)
Control group	153	2.13 (1.04)	2.34 (1.12)	2.40 (1.13)	2.59 (1.28)	2.80 (1.31)
*t*-value		−0.767	−0.992	−1.647	−0.907	−0.990
*P*-value		0.444	0.322	0.101	0.365	0.323

GDS-C, Griffiths Mental Development Scale-Chinese.

### Developmental differences between the two groups before and after the intervention

During the follow-up period, the participation of 89 children was withdrawn. Consequently, a total of 217 24-month-year old children with GDD (i.e., 101 and 116 children in the experimental and control group, respectively) completed the assessment. Children with GDD showed significant improvements in development, as assessed by the GDS-C. The results of paired *t*-test showed that the DQ of locomotor, personal-social, language, eye-hand coordination, and performance, as well as the total GQ score in the experimental group, changed significantly after the PIEIP intervention (*P* < 0.001 for all) (Table 3). Moreover, significant differences were also found in the mean DQ of the above GDS-C subscales as well as the total GQ score in the control group (*P* < 0.001 for all).

**Table 3:** Comparison of pretest and posttest of the developmental quotient between two groups (GDS-C).

Variable groups	Pretest (T1)		Posttest (T2)		Difference (T2–T1)		t-value	*P*-value
	Mean	SD	Mean	SD	Mean	SD		
**Locomotor (A)**							2.034^a^	0.044^a^^,^ *
Intervention group	45.43	18.29	85.04	14.92	39.60	23.73	14.643^b^	<0.001^b^^,^ *
Control group	49.23	24.94	79.20	17.09	29.97	32.31	7.704^b^	<0.001^b^^,^ *
**Personal-Social (B)**							2.150^a^	0.033^a^^,^ *
Intervention group	50.29	22.79	86.71	13.27	36.43	27.55	11.601^b^	<0.001^b^^,^ *
Control group	52.07	27.46	77.69	17.09	25.61	33.19	6.411^b^	<0.001^b^^,^ *
**Language (C)**							3.109^a^	0.002^a^^,^ *
Intervention group	48.81	22.41	89.26	15.83	40.44	26.22	13.535^b^	<0.001^b^^,^ *
Control group	52.96	28.53	78.26	17.65	25.30	32.56	6.453^b^	<0.001^b^^,^ *
**Eye-hand coordination (D)**							1.595^a^	0.113^a^
Intervention group	54.53	24.09	84.20	14.34	29.67	27.88	9.338^b^	<0.001^b^^,^ *
Control group	59.84	31.55	80.76	16.58	20.92	38.06	4.567^b^	<0.001^b^^,^ *
**Performance (E)**							1.394^a^	0.165^a^
Intervention group	57.62	23.40	85.04	16.23	27.42	30.18	7.974^b^	<0.001^b^^,^ *
Control group	59.65	32.10	79.44	17.93	19.79	35.90	4.580^b^	<0.001^b^^,^ *
**Total**							2.363^a^	0.019^a^^,^ *
Intervention group	51.34	12.53	84.83	12.74	33.49	19.44	15.116^b^	<0.001^b^^,^ *
Control group	54.75	21.04	79.07	15.44	24.32	27.18	7.433^b^	<0.001^b^^,^ *

GDS-C, Griffiths Mental Development Scale-Chinese.

^a^ Independent-sample *t*-test between two groups for the developmental progression [Difference (T2–T1)].

^b^ Paired *t*-test within groups for the Pretest (T1) and Posttest (T2).

* *P-*value <0.05.

In the control group, locomotor ability exhibited the most significant progress, achieving an average improvement of 29.97, followed by personal-social with 25.61. However, the performance ability yielded the lowest progress, achieving an average improvement of 19.79.

After the PIEIP intervention, the language DQ score demonstrated the most progress, achieving an average improvement of 40.44 in the experimental group, followed by locomotor, with 39.60. However, the performance ability exhibited the least progress, with an average improvement of 27.42.

After continuous intervention until GDD child reached 2 years of age, an independent *t*-test was used to make a comparison of the progress between two groups to confirm the effectiveness of the PIEIP. The total GQ score of the experimental group made an increase of 33.49, and that of the control group made an increase of 24.32. This development progress before and after the intervention of two groups had a statistically significant difference (*P* = 0.019). It indicates that the overall progress of development in the experimental group is more than that in the control group. The comparative analysis on the differences in the progress between two groups by independent *t*-test also shows that, after experimental intervention, for DQ of locomotor (*P* = 0.044), personal-social (*P* = 0.033) and language (*P* = 0.002), but not eye-hand coordination (*P* = 0.113) and performance (*P* = 0.165), the children in experimental group made more progress than those in the control group (Table 3). From the above results, it can be inferred that PIEIP has significant positive effects on the development of children with GDD, particularly in the locomotor, personal-social and language domains.

### Comparison of the differences in pretest and posttest parenting stress standard scores between the two groups

The results of paired *t*-test showed that, after PIEIP interference, the children with GDD in the experimental group exhibited a significant decrease in the total level of parental stress, mean standard score of dysfunctional interaction, and difficult children (*P* < 0.001 for all) (Table 4). Similarly, the mean standard score for dysfunctional interaction and the total standard scores significantly decreased in the control group (*P* < 0.001 for both). However, the mean standard score of children with difficulties showed no significant change between the pretest and posttest. An independent *t*-test was used to make a comparison of the parenting stress changes between two groups. The analysis result shows that, no significant difference was observed for the change in each sub-scale and the total score between the two groups (*P* > 0.05) (Table 4). This indicates that the addition of PIEIP intervention has no significant effect on further reducing each subscale scores and total scores of parenting stress.

**Table 4:** Comparison of pretest and posttest parenting stress standard scores between two groups (PSI/SF).

Variable groups	Pretest (T1)		Posttest (T2)		Difference (T2–T1)		t-value	*P*-value
	Mean	SD	Mean	SD	Mean	SD		
**Parenting distress**							0.296^a^	0.769^a^
Intervention group	33.26	9.28	30.61	12.67	−2.65	15.01	−0.981^b^	0.334^b^
Control group	29.53	12.80	25.73	9.92	−3.80	15.50	−1.343^b^	0.190^b^
**Dysfunctional interaction**							−1.613^a^	0.112^a^
Intervention group	27.29	8.52	16.68	4.76	−10.61	10.05	−5.878^b^	<0.001^b^^,^ *
Control group	29.93	9.74	23.47	6.34	−6.47	10.03	−3.533^b^	0.001^b^^,^ *
**Difficult child**							−1.208^a^	0.232^a^
Intervention group	30.42	8.80	22.97	9.11	−7.45	13.22	−3.139^b^	0.004^b^^,^ *
Control group	28.20	8.70	24.87	9.79	−3.33	13.42	−1.361^b^	0.184^b^
**PSI total**							−1.036^a^	0.304^a^
Intervention group	90.97	20.03	70.26	17.45	−20.71	29.51	−3.907^b^	<0.001^b^^,^ *
Control group	87.67	23.33	74.07	17.45	−13.60	23.63	−3.152^b^	0.004^b^^,^ *

PSI/SF, parental stress index-short form.

^a^ Independent-sample *t*-test between two groups for the parenting stress changes [Difference (T2–T1)].

^b^ Paired *t*-test within groups for the Pretest (T1) and Posttest (T2).

* *P-*value <0.05.

### Prediction of improvement based on characteristics of parents and children

A multivariate linear regression analysis was performed and the relationships between the factors and their improvement among children and parents are shown in Table 5. For child gender, family income, developmental age-equivalent at baseline, parents' educational level, birth weight, parents' age, and the age of children when recruited didn't have statistical difference between groups, these factors were implemented as covariates into a regression model. And the independent variables included the pretest and posttest parenting stress standard score changes and gestational age. Specifically, 0.05 and 0.1 were set as the entry and removal levels, respectively (model: stepwise). Furthermore, the significant factors (*P* < 0.05) are shown in Table 5. The group factor was significant for most of the test items. Moreover, a decrease in parenting distress contributed significantly to the improvement in locomotor, language, eye-hand coordination, and performance domains (change in DQ score) among children with GDD.

**Table 5:** Prediction of improvement by pre-intervention scores and subject characteristics.

Dependent variable	Factor	Multivariate coefficient	SD	t-value	*P*-value
DQ_Locomotor (T2-T1)	Group	−15.26	4.62	−3.306	0.002
	Parenting distress (T2-T1)	−0.64	0.24	−2.612	0.012
DQ_Personal-Social (T2-T1)	Group	−15.68	4.09	−3.837	0.000
DQ_Language (T2-T1)	Group	−13.69	4.57	−2.995	0.005
	Parenting distress (T2-T1)	−0.59	0.24	−2.434	0.019
	Gestational age	−1.68	0.84	−2.165	0.044
DQ_Eye-hand coordination (T2-T1)	Group	−13.71	4.48	−3.061	0.004
	Parenting distress (T2-T1)	−0.65	0.27	−2.208	0.012
	Dysfunctional interaction (T2-T1)	0.78	0.28	1.996	0.035
DQ_Performance (T2-T1)	Group	−19.67	4.30	−4.573	0.000
	Parenting distress (T2-T1)	−0.71	0.25	−2.373	0.035
GQ (T2-T1)	Group	−15.60	3.92	−3.979	0.000
	Dysfunctional interaction (T2-T1)	0.55	0.21	2.649	0.011

DQ, developmental quotient GQ, general quotient.

Note. The independent factors and coefficient are shown if they had a significant influence on the improvement of test items.

## Discussion

It is widely accepted that a life routine plays the most significant role in the relationship between parental psychology and parenting. The more normal the children's daily life is, the more regularly the intervention can be implemented to yield more of an obvious effect (24). Numerous studies have shown that the cognitive, social, and emotional development of children with TD improves in the context of good interaction between caregivers and children (25, 26). Functional and effective interactions positively influence children's cognitive abilities and overall development. However, experimental studies on the effect of parental participation on the developmental outcomes of children with GDD are limited (16, 17, 27).

In 2018, a study investigated the effect of parental involvement in occupational therapy on treatment outcomes in children with GDD. The study included 30 pairs of children with developmental delay (average age 46.8 ± 16.0 months) and their parents. The cognitive, social, motor, language, and self-care abilities of the children with GDD improved with increased parental involvement in treatment. This intervention is conducted primarily in a medical setting (i.e., a therapeutic room), in which parents watch or act as collaborative therapy personnel (17). Some researchers have used HAP in children with GDD. Therapists have designed the HAP to help children achieve specific goals in their daily lives. HAP is often used to supplement traditional rehabilitation training in hospitals or as an alternative intervention particularly when parents cannot bring their children to hospitals regularly. Tétreault et al. (27) found that families comprising children with GDD showed good adherence to the HAP program. Tang et al. (16) found that children who participated in HAP demonstrated more advanced progress in language, social, cognitive, and motor domains, except for self-help as compared to children who only participated in the weekly interviews. However, the duration of the treatment period was only 12 weeks. Furthermore, the overall sample size was relatively small (*n* = 70 in total) comprising participants of older age at enrollment (average age 20.7 ± 10.0 months).

There were obvious differences between our PIEIP intervention and the above-mentioned studies which also included parents in the early intervention. The PIEIP carried out closed-loop and progressive family intervention from early life (i.e., 3–6 months) until the age of two years through several processes. Namely, evaluating the current development level of children, formulating a family training plan for the subsequent 8–12 weeks, parent class correspondence with the current development level of children, family intervention implementation and parents' intervention technical feedback, regular follow-up, and the following round of parent class and family intervention implementation. Thus, in PIEIP, family members are the main body of intervention, and professionals serve as training, supervision and evaluation, forming an intervention mode of long-term cooperation and joint implementation. Through PIEIP, children with GDD can receive early and higher-frequency intervention in the family environment.

Here, compared with baseline scores, all developmental scores were improved at 2 years of age. Children who received further PIEIP demonstrated significant improvements in locomotor, personal-social, language, and general development, but not eye-hand coordination and performance, as compared to the control group. Thus, the addition of FECIP has a positive effect on the improvement of GDD children’s outcomes, especially in the field of locomotor, personal-social and language. PIEIP intervention is a naturalistic developmental–behavioral intervention. This is a class of interventions for young children with GDD that has been informed by the fields of developmental and communication sciences and applied behavior analysis. It uses a unique blend of developmental and behavioral intervention techniques. They are designed to increase the parent's responsiveness to the child's behavior and teach the child to use new communication, imitation, and play skills within ongoing interactions in daily routines. This may be the main reason for the rapid improvement of children’s personal-social and language ability after PIEIP intervention. As for the promotion of locomotor, it may be related to the fact that PIEIP intervention also introduced many parent-child sports games which are suitable for family and community environments to parents.

Parenting stress can destroy family resources and parenting efficacy, which in turn, negatively impacts the development of the child (28). Parents of children with GDD showed higher levels of stress than parents of children with TD, further affecting parent-child interaction (19). Behavioral problems among children are influenced by their parents’ negative emotions such as stress-induced restlessness. Parental involvement in early interventions for children with NDDs, such as ASD, ADHD, and GDD can increase parental pressure. However, the current study observed a significant decrease in the total level of parental stress and several subdomains at two years of age in both the experimental and control groups. And the variation in the changes between the two groups showed no statistical differences for each sub-scale or the total score. We speculated that the decrease in parenting stress from applying the PIEIP intervention may be because of established positive relationships between parents and doctors. Furthermore, by using the PIEIP, doctors can help children more effectively as it is recognized by the majority of parents. In addition, our multiple regression analysis indicated that the change in the parental stress score might be associated with the outcome of the child. These findings are consistent with previous studies on intervention models for children with NDDs, in which parental stress, especially mothers' stress, emerged as a predictor (18, 29–31). Thus, we conclude that parental stress affects parents' ability to complete home training.

This study was advantageous because it comprised a multicenter intervention across multiple geographic locations in China. However, this study had some limitations. First, this study was not a randomized controlled trial (RCT) but allowed parents to voluntarily choose whether to participate in the PIEIP intervention, which may lead to inherent bias on parental variables. There are many structural barriers that can affect a parent's ability to access and participate in parent-mediated intervention, including child care, work schedule, transportation, and other family responsibilities or life stressors (32). On the other hand, some parents of newly diagnosed children may not yet be emotionally ready to process information and apply it to their children (33). These parents may need a period of grieving before they can fully benefit from a parent-mediated intervention program. Instead, parents who participated in the PIEIP may be those who have more flexibility to participate in these sorts of parent-mediated interventions, and those who have strong intrinsic motivation and believe that their own efforts can improve the development of their children (34, 35). Therefore, we cannot totally attribute the effects to the intervention itself. In addition to parental stress, other psychological factors such as executive function and self-efficacy should also be considered important parental variables for family interventions. Moreover, these should be added to the regression model for further analysis. Furthermore, in the future, we can try to balance these factors by conducting a rigorously designed RCT study to explore the role of PIEIP itself in the developmental outcome of GDD children. Second, we did not collect and compare data based on the content, frequency, and intensity of other institution-based interventions in which the individuals might have participated. Thus, this variable could potentially affect the development outcomes of the two groups of children. Third, the current study conducted interventions targeting individuals from 3 to 6 months after birth until 2 years of age. Although parents regularly brought their children to the hospital for assessment, attended parental classes, and periodically delivered family training videos, their detailed training time and quality of intervention at home were not monitored in this study. Fourth, the rate of lost to follow-up in this study was relatively high, especially in the intervention group. In the future, we will choose a more stable population in the research and design stage, arrange the time of follow-up and parent class in the evening or weekend, carry out distance learning, establish better interpersonal communication with the parents, clarify the meaning of the project more clearly to parents and the importance of follow-up, provide additional consultation and referral services, and continue to provide follow-up services for the lost population to maximize follow-up and compliance.

## Conclusions

We found that the PIEIP is effective as an intervention to improve the development of children with GDD. Using the PIEIP as a supplement to traditional interventions in hospitals or communities can improve the developmental outcomes of children with GDD at the age of two years. Particularly, parental involvement and expansion of capacity acquisition in the natural environment are important throughout the process. In the future, more extensive intervention and follow-up studies are required that include measures such as executive function, parent-child interaction factors, self-efficacy, and other physiological risk factors to better elucidate the underlying pathways linking parent-based early intervention with child outcomes.

## Data Availability

The original contributions presented in the study are included in the article/Supplementary Material, further inquiries can be directed to the corresponding author/s.
