# Supplementary material for: A multicenter clinical study on parent-implemented early intervention for children with global developmental delay
Source: Front Pediatr. 2023 Feb 15;11:1052665. doi: 10.3389/fped.2023.1052665 (PMC9975705; doi:10.3389/fped.2023.1052665)
Supplement: Supplementary file 2 [file Table2.pdf]

Table S2. The relevant code/script file for the data analysis

```
  FILE='/Users/jasmine/Documents/工作/我的科研课题/GDD早期家庭干预队列/GDD 数据集.sav'.
DATASET NAME 数据集1 WINDOW=FRONT.
MEANS TABLES=基线月龄 BY 组别
  /CELLS=MEAN COUNT STDDEV RANGE MIN MAX.
```

平均值

备注

已创建输出		27-JUN-2022 09:20:32
注释		
输入	数据	/Users/jasmine/Documents/工作/我的科研课题/GDD早期家庭干预队列/GDD 数据集.sav
	活动数据集	数据集1
	过滤器	<无>
	权重	<无>
	拆分文件	<无>
	工作数据文件中的行数	306
缺失值处理	对缺失的定义	对于表中的每个因变量，将因变量的用户定义缺失值视为缺失。
	使用的个案数	用于每个表的个案在任何自变量中都不具有缺失值，并非所有因变量都具有缺失值。
语法		MEANS TABLES=基线月龄 BY 组别 /CELLS=MEAN COUNT STDDEV RANGE MIN MAX.
资源	处理程序时间	00:00:00.00
	耗用时间	00:00:00.00

[数据集1] /Users/jasmine/Documents/工作/我的科研课题/GDD早期家庭干预队列/GDD 数据集.sav

个案处理摘要

	个案					
	包括		排除		总计	
	个案数	百分比	个案数	百分比	个案数	百分比
基线月龄 * 组别	306	100.0%	0	0.0%	306	100.0%

报告

基线月龄

组别	平均值	个案数	标准 偏差	范围	最小值	最大值
干预组	4.5595	153	1.07651	3.90	2.90	6.80
对照组	4.4948	153	1.03941	3.90	3.00	6.90
总计	4.5271	306	1.05688	4.00	2.90	6.90

```
T-TEST GROUPS=组别(1 2)
  /MISSING=ANALYSIS
  /VARIABLES=基线月龄
  /CRITERIA=CI(.95).
```

T-检验

备注

已创建输出		27-JUN-2022 09:37:23
注释		
输入	数据	/Users/jasmine/Documents/工作/我的科研课题/GDD早期家庭干预队列/GDD 数据集.sav
	活动数据集	数据集1
	过滤器	<无>
	权重	<无>
	拆分文件	<无>
	工作数据文件中的行数	306
缺失值处理	对缺失的定义	将用户定义的缺失值视为缺失。

	使用的个案数	每项分析的统计都基于符合以下条件的个案：对于该分析中的任何变量，都不具有缺失数据或超范围数据。
语法		T-TEST GROUPS=组别(1 2)<br>/MISSING=ANALYSIS<br>/VARIABLES=基线月龄<br>/CRITERIA=CI(.95).
资源	处理程序时间	00:00:00.00
	耗用时间	00:00:00.00

组统计

	组别	个案数	平均值	标准 偏差	标准 误差平均值
基线月龄	干预组	153	4.5595	1.07651	.08703
	对照组	153	4.4948	1.03941	.08403

独立样本检验

		莱文方差等同性检验		平均值等同性 t 检验						
		F	显著性	t	自由度	Sig.（双尾）	平均值差值	标准误差差值		
基线月龄	假定等方差	.845	.359	.535	304	.593	.06471	.12098		
	不假定等方差			.535	303.627	.593	.06471	.12098		

```
EXAMINE VARIABLES=出生胎龄 BY 组别
  /ID=患者编号
  /PLOT BOXPLOT STEMLEAF HISTOGRAM NPPLOT SPREADLEVEL(1)
  /COMPARE GROUPS
  /MESTIMATORS HUBER(1.339) ANDREW(1.34) HAMPEL(1.7,3.4,8.5) TUKEY(4.685)
  /PERCENTILES(5,10,25,50,75,90,95) HAVERAGE
  /STATISTICS DESCRIPTIVES EXTREME
  /CINTERVAL 95
  /MISSING LISTWISE
  /NOTOTAL.
```

探索

备注

已创建输出		27-JUN-2022 10:11:48
注释		
输入	数据	/Users/jasmine/Documents/工作/我的科研课题/GDD早期家庭干预队列/GDD 数据集.sav
	活动数据集	数据集1
	过滤器	<无>
	权重	<无>
	拆分文件	<无>
	工作数据文件中的行数	306
缺失值处理	对缺失的定义	将因变量的用户定义缺失值视为缺失。
	使用的个案数	统计基于那些对任何所用因变量或因子都没有缺失值的个案。
语法		EXAMINE VARIABLES=出生胎龄 BY 组别<br>/ID=患者编号<br>/PLOT BOXPLOT STEMLEAF HISTOGRAM NPPLOT SPREADLEVEL(1)<br>/COMPARE GROUPS<br>/MESTIMATORS HUBER(1.339) ANDREW(1.34) HAMPEL(1.7,3.4,8.5) TUKEY(4.685)<br>/PERCENTILES(5,10,25,50,75,90,95) HAVERAGE<br>/STATISTICS DESCRIPTIVES EXTREME<br>/CINTERVAL 95<br>/MISSING LISTWISE<br>/NOTOTAL.
资源	处理程序时间	00:00:02.65
	耗用时间	00:00:02.00

组别

个案处理摘要

	组别	有效 N	有效 百分比	缺失 N	缺失 百分比	总计 N	总计 百分比
出生胎龄	干预组	153	100.0%	0	0.0%	153	100.0%
	对照组	153	100.0%	0	0.0%	153	100.0%

描述

	组别			统计	标准误差
出生胎龄	干预组	平均值		36.679	.2402
		平均值的 95% 置信区间	下限	36.205	
			上限	37.154	
		5% 剪除后平均值		36.956	
		中位数		37.600	
		方差		8.828	
		标准偏差		2.9712	
		最小值		27.2	
		最大值		40.2	
		范围		13.0	
		四分位距		1.2	
		偏度		-1.585	.196
		峰度		1.785	.390
	对照组	平均值		37.163	.2271
		平均值的 95% 置信区间	下限	36.714	
			上限	37.611	
		5% 剪除后平均值		37.382	
		中位数		38.000	
		方差		7.891	
		标准偏差		2.8092	
		最小值		28.0	
		最大值		42.0	
		范围		14.0	
		四分位距		2.0	
		偏度		-1.417	.196
		峰度		1.679	.390

M 估计量

	组别	休伯 M 估计量[a]	图基双权[b]	汉佩尔 M 估计量[c]	安德鲁波[d]
出生胎龄	干预组	37.551	37.731	37.777	37.727

	对照组	37.896	38.146	38.028	38.146

a. 加权常量为 1.339。
b. 加权常量为 4.685。
c. 加权常量为 1.700、3.400 和 8.500
d. 加权常量为 1.340*pi。

百分位数

			百分位数						
		组别	5	10	25	50	75	90	95
加权平均（定义 1）	出生胎龄	干预组	29.760	31.280	37.000	37.600	38.150	39.060	40.000
		对照组	30.100	32.840	37.000	38.000	39.000	40.000	40.220
图基枢组	出生胎龄	干预组			37.000	37.600	38.100		
		对照组			37.000	38.000	39.000		

极值

组别				个案号	患者编号	值
出生胎龄	干预组	最大值	1	41	41	40.2
			2	13	13	40.0
			3	23	23	40.0
			4	29	29	40.0
			5	31	31	40.0[a]
		最小值	1	95	95	27.2
			2	135	135	28.0
			3	134	134	28.0
			4	49	49	28.2
			5	48	48	28.2
	对照组	最大值	1	169	16	42.0
			2	167	14	41.0
			3	198	45	41.0
			4	240	87	41.0
			5	246	93	41.0[b]
		最小值	1	296	143	28.0
			2	164	11	28.0
			3	163	10	29.0
			4	295	142	30.0
			5	157	4	30.0

a. 在较大极值的表中，仅显示了不完整的个案列表（这些个案的值为 40.0）。
b. 在较大极值的表中，仅显示了不完整的个案列表（这些个案的值为 41.0）。

正态性检验

	组别	柯尔莫戈洛夫-斯米诺夫(V)[a] 统计	自由度	显著性	夏皮洛-威尔克 统计	自由度	显著性
出生胎龄	干预组	.314	153	.000	.791	153	.000
	对照组	.235	153	.000	.852	153	.000

a. 里利氏显著性修正

方差齐性检验

		莱文统计	自由度 1	自由度 2	显著性
出生胎龄	基于平均值	.239	1	304	.625
	基于中位数	.014	1	304	.907
	基于中位数并具有调整后自由度	.014	1	301.695	.907
	基于剪除后平均值	.004	1	304	.947

出生胎龄

直方图(O)

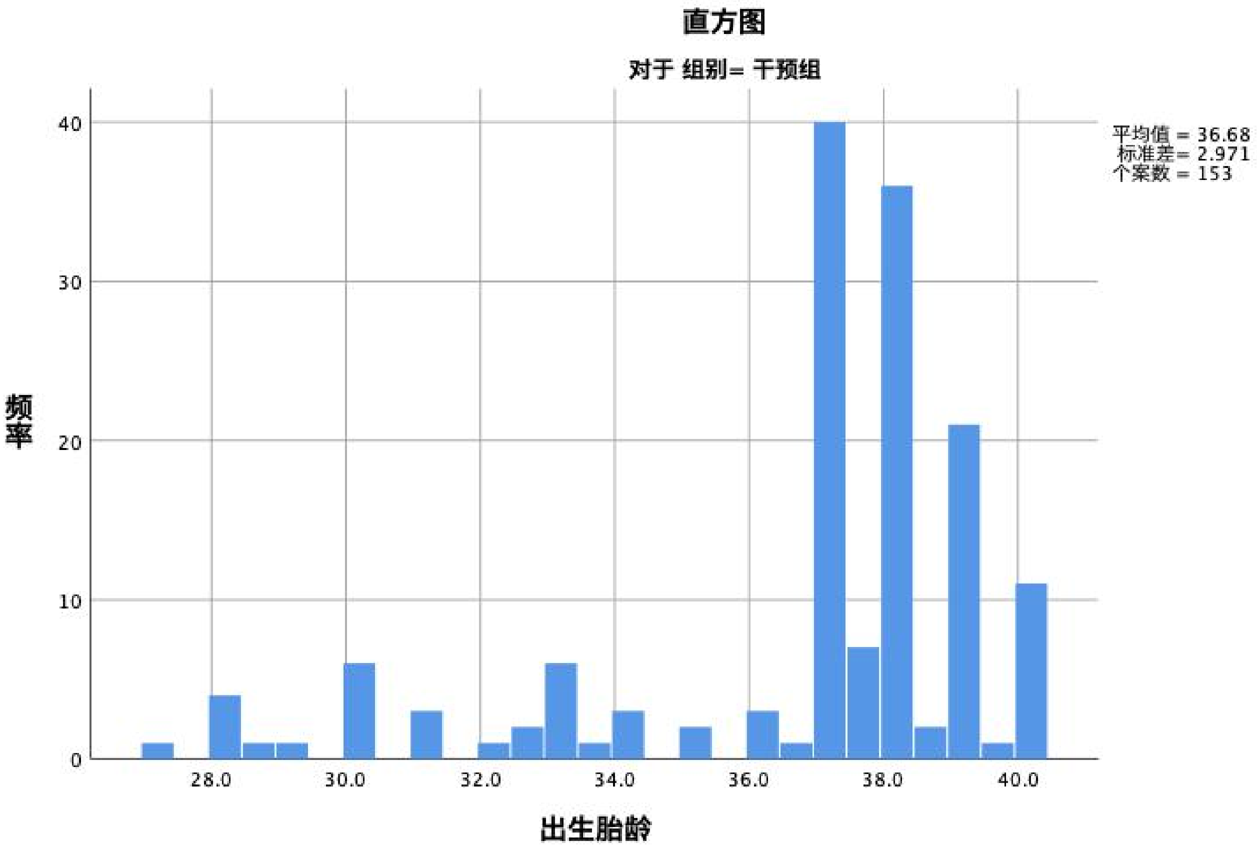

直方图
对于 组别= 干预组
平均值 = 36.68
标准差= 2.971
个案数 = 153
频率
出生胎龄

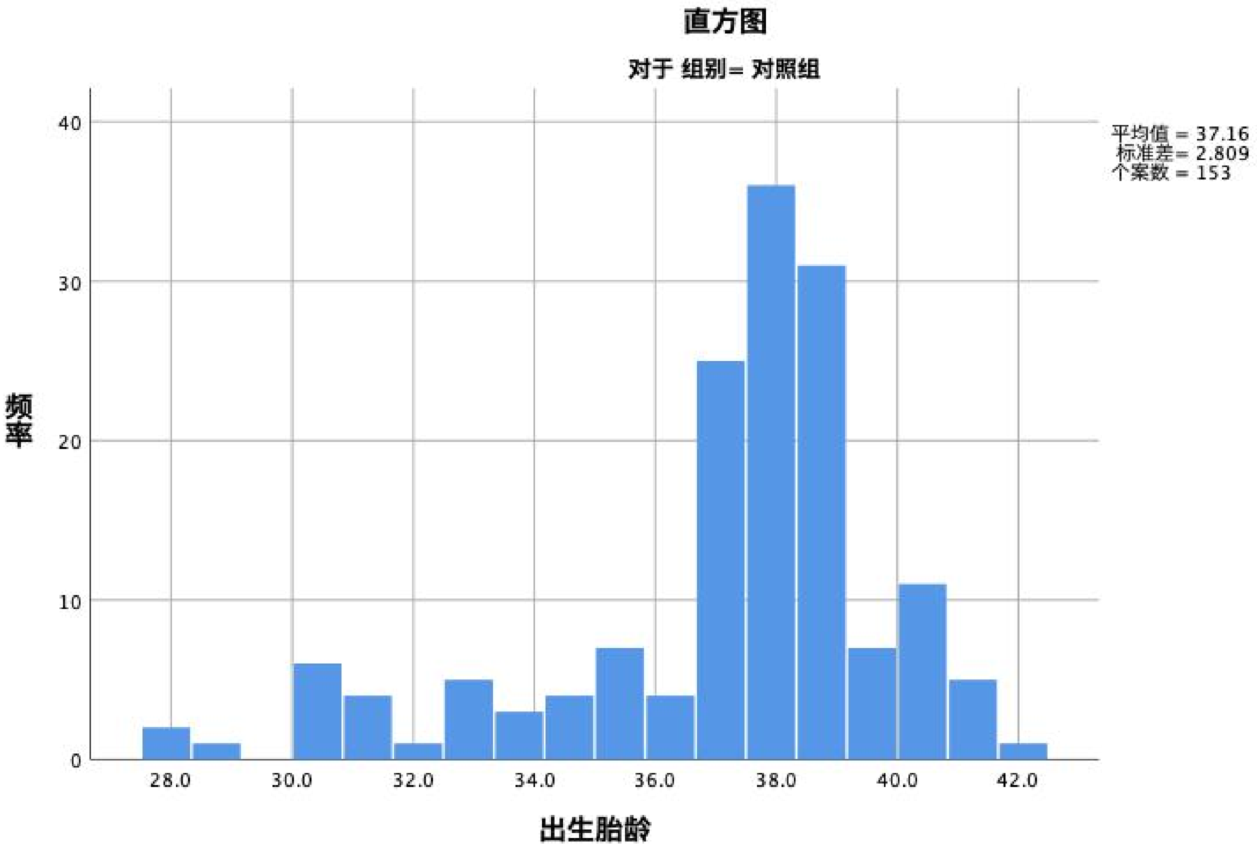

直方图
对于 组别= 对照组
平均值 = 37.16
标准差= 2.809
个案数 = 153
频率
出生胎龄

茎叶图

出生胎龄 茎叶图：
组别= 干预组

频率	Stem &	叶
31.00	Extremes	(=<35.0)
2.00	36 .	01
1.00	36 .	3
1.00	36 .	5
.00	36 .	
.00	36 .	
29.00	37 .	00000000000000000000000011111
10.00	37 .	2222223333
2.00	37 .	45
6.00	37 .	666666
.00	37 .	
33.00	38 .	000000000000000000000000000000111
2.00	38 .	22
3.00	38 .	455
.00	38 .	
.00	38 .	
20.00	39 .	00000000000000000011
.00	39 .	
2.00	39 .	45
11.00	极值	(>=40.0)

主干宽度： 1.0
每个叶： 1 个案

出生胎龄 茎叶图：
组别= 对照组

频率	Stem &	叶
22.00	Extremes	(=<33.5)
3.00	34 .	444
1.00	34 .	5
.00	35 .	
7.00	35 .	5555566
1.00	36 .	3
3.00	36 .	556
25.00	37 .	0000000000000111222233344

4.00	37 .	5556
34.00	38 .	0000000000000000000011112222222344
6.00	38 .	555556
30.00	39 .	000000000000000011111112222223
.00	39 .	
10.00	40 .	0000000001
1.00	40 .	5
5.00	41 .	00000
.00	41 .	
1.00	42 .	0

主干宽度：　　1.0
每个叶：　　1 个案

正态 Q-Q 图

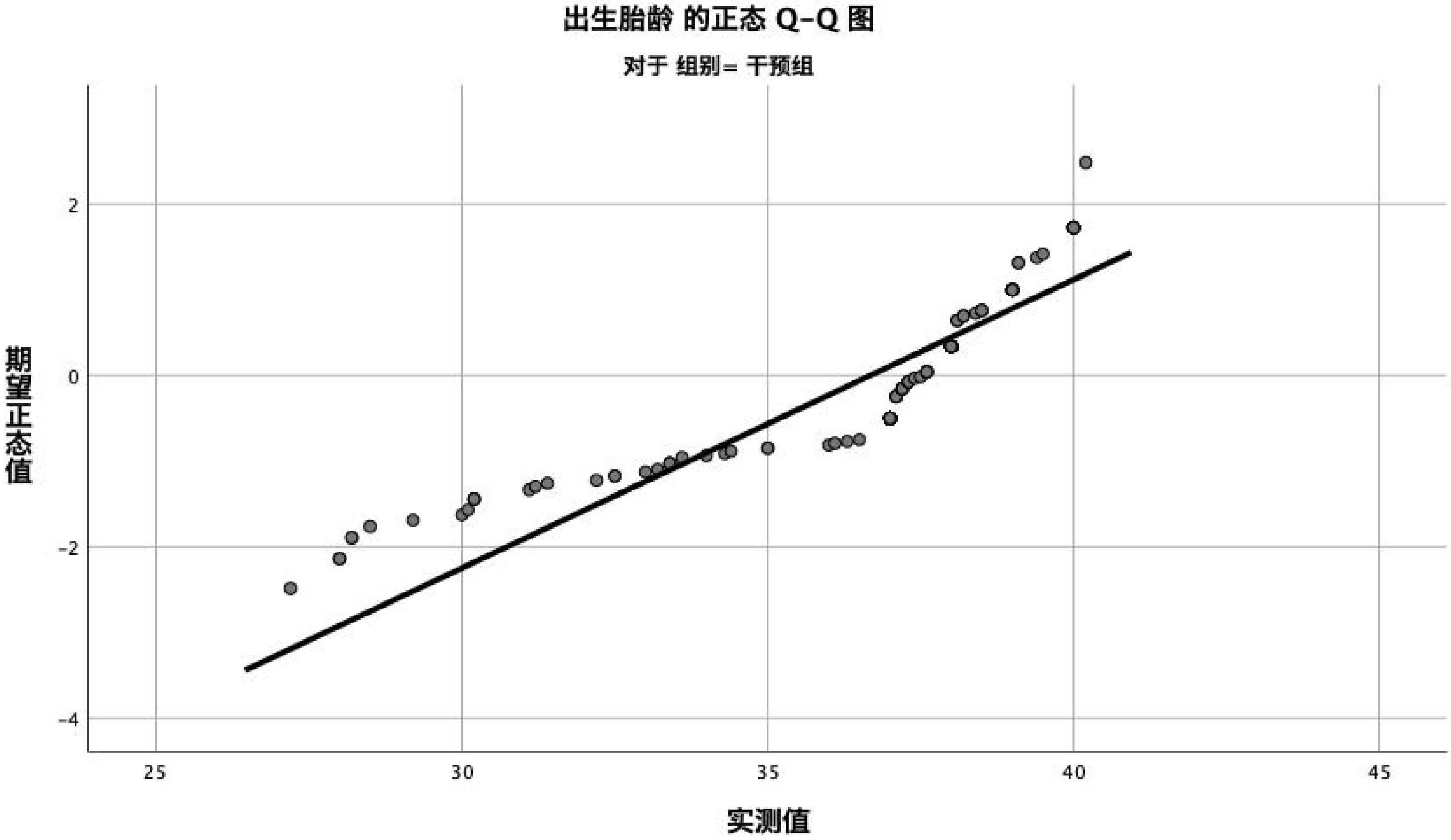

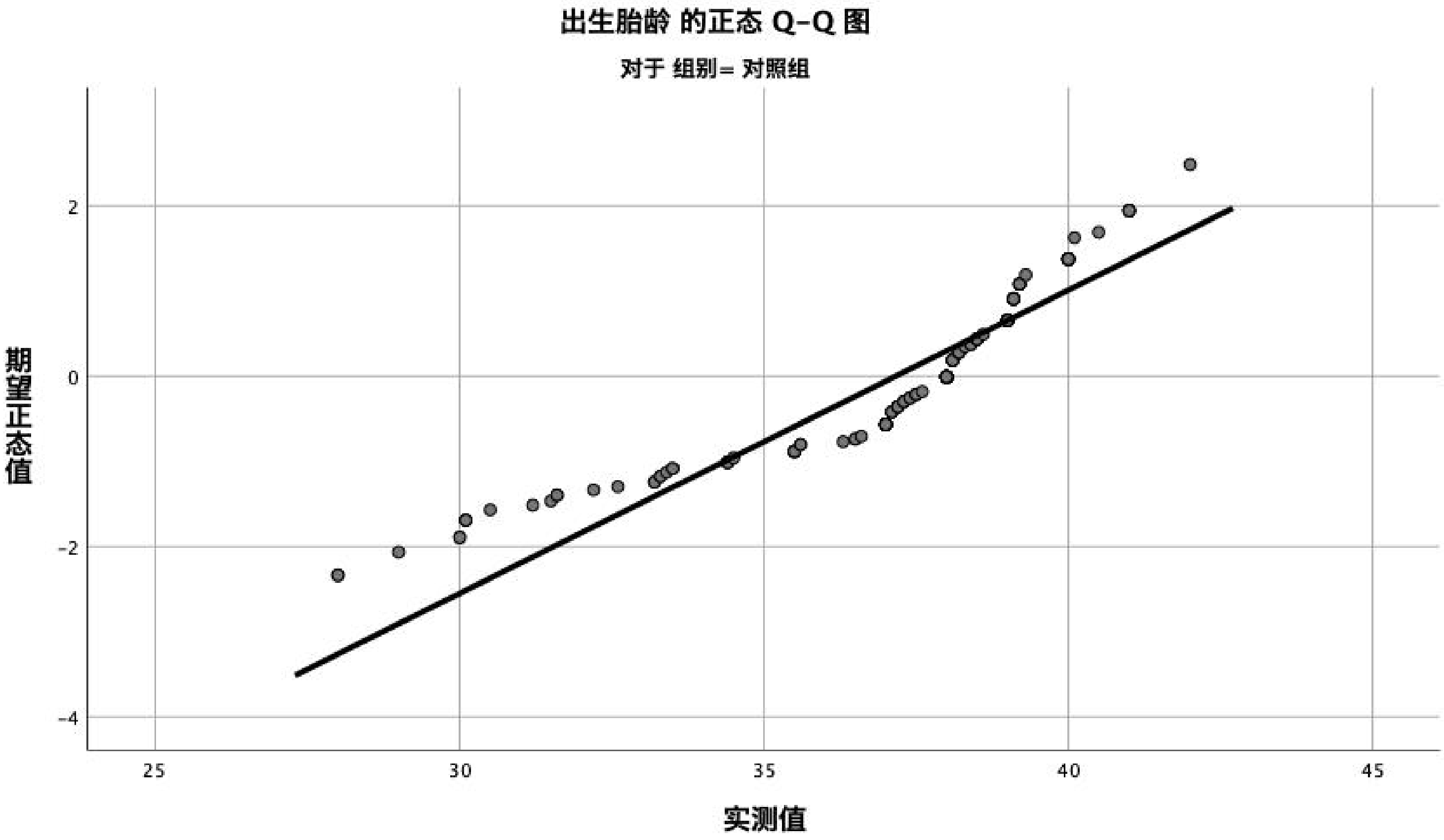

去趋势正态 Q-Q 图

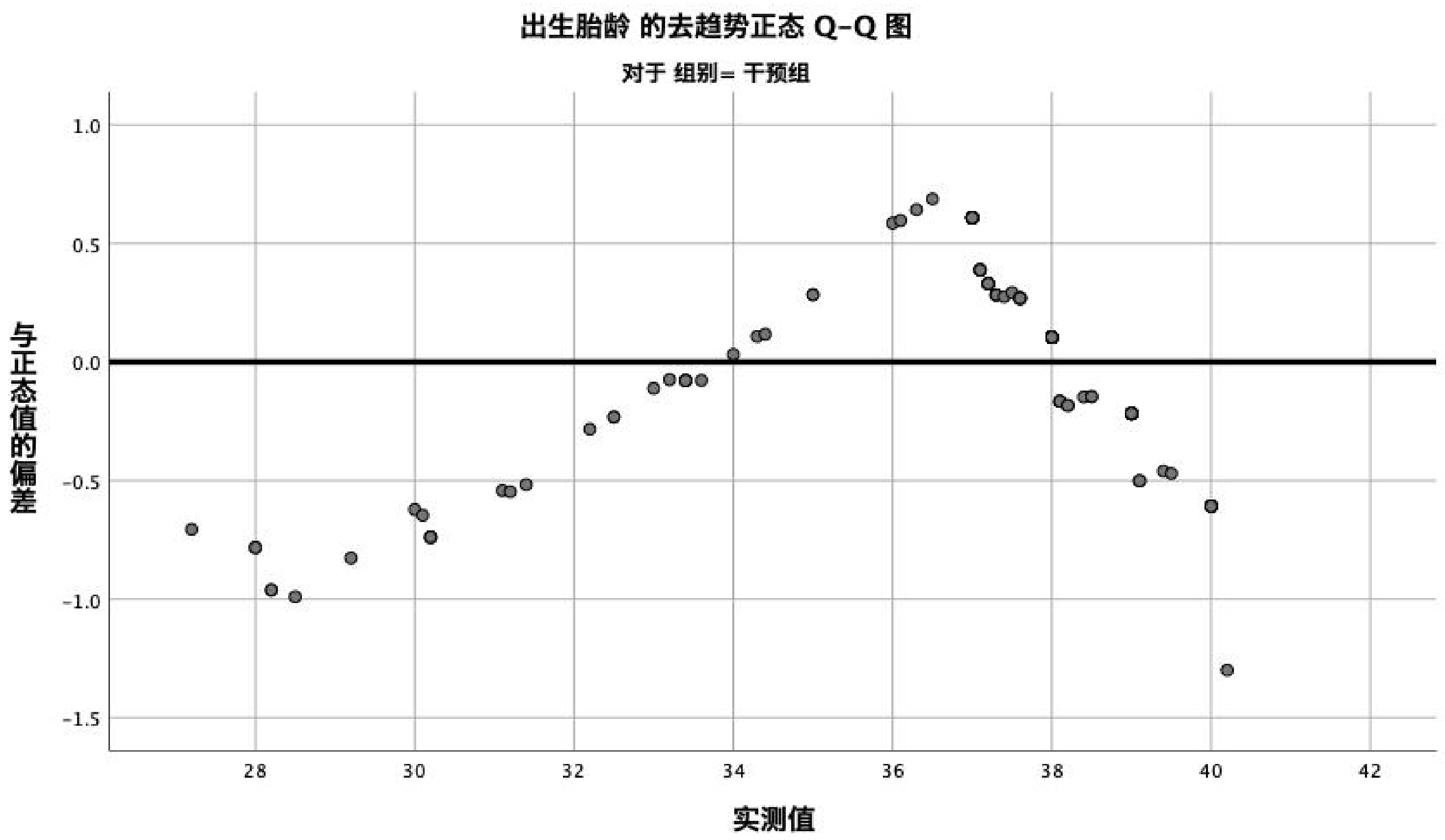

出生胎龄 的去趋势正态 Q-Q 图
对于 组别= 干预组
1.0
0.5
0.0
-0.5
-1.0
-1.5
与正态值的偏差
28
30
32
34
36
38
40
42
实测值

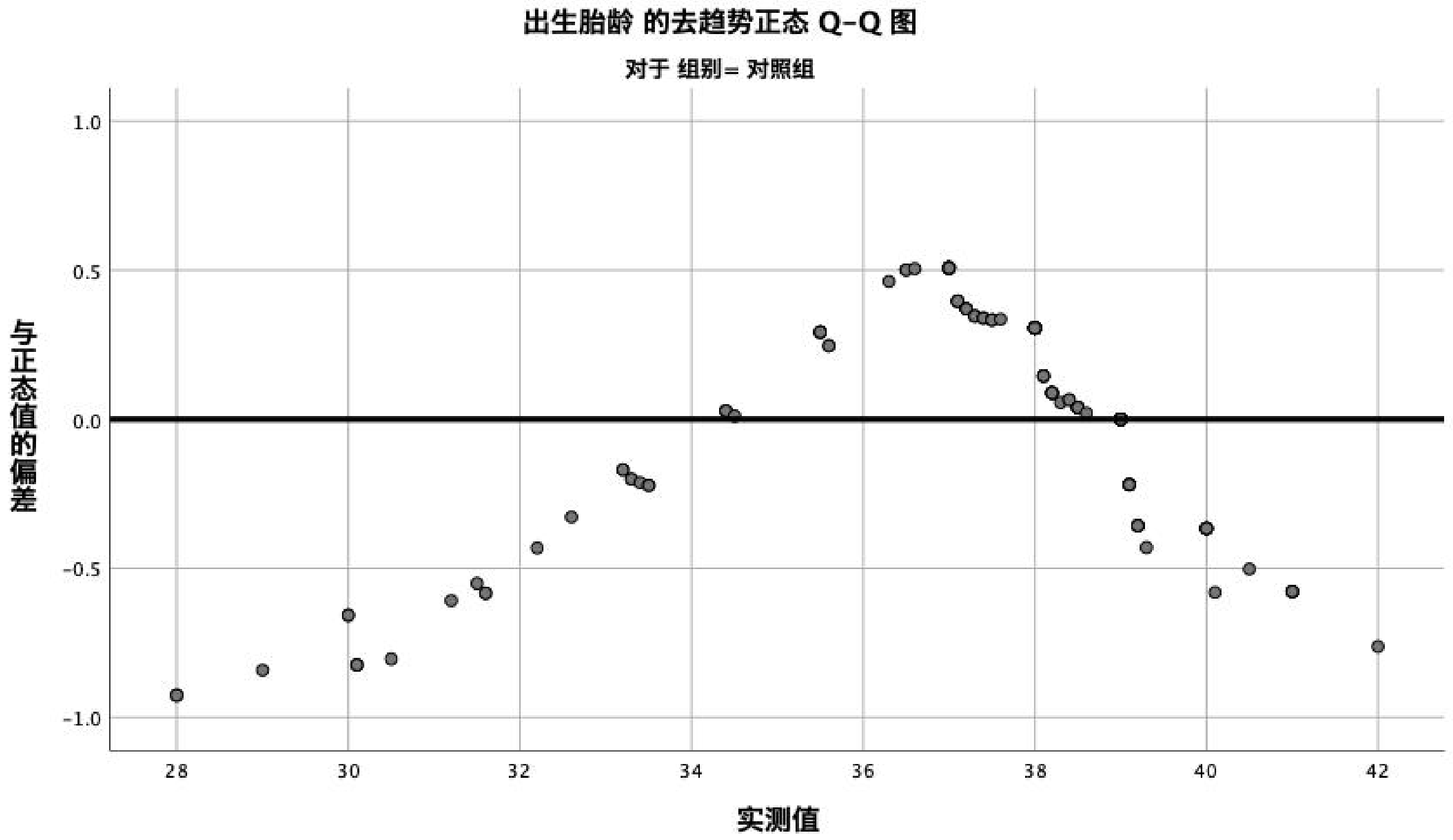

出生胎龄 的去趋势正态 Q-Q 图
对于 组别= 对照组
1.0
0.5
0.0
-0.5
-1.0
与正态值的偏差
28
30
32
34
36
38
40
42
实测值

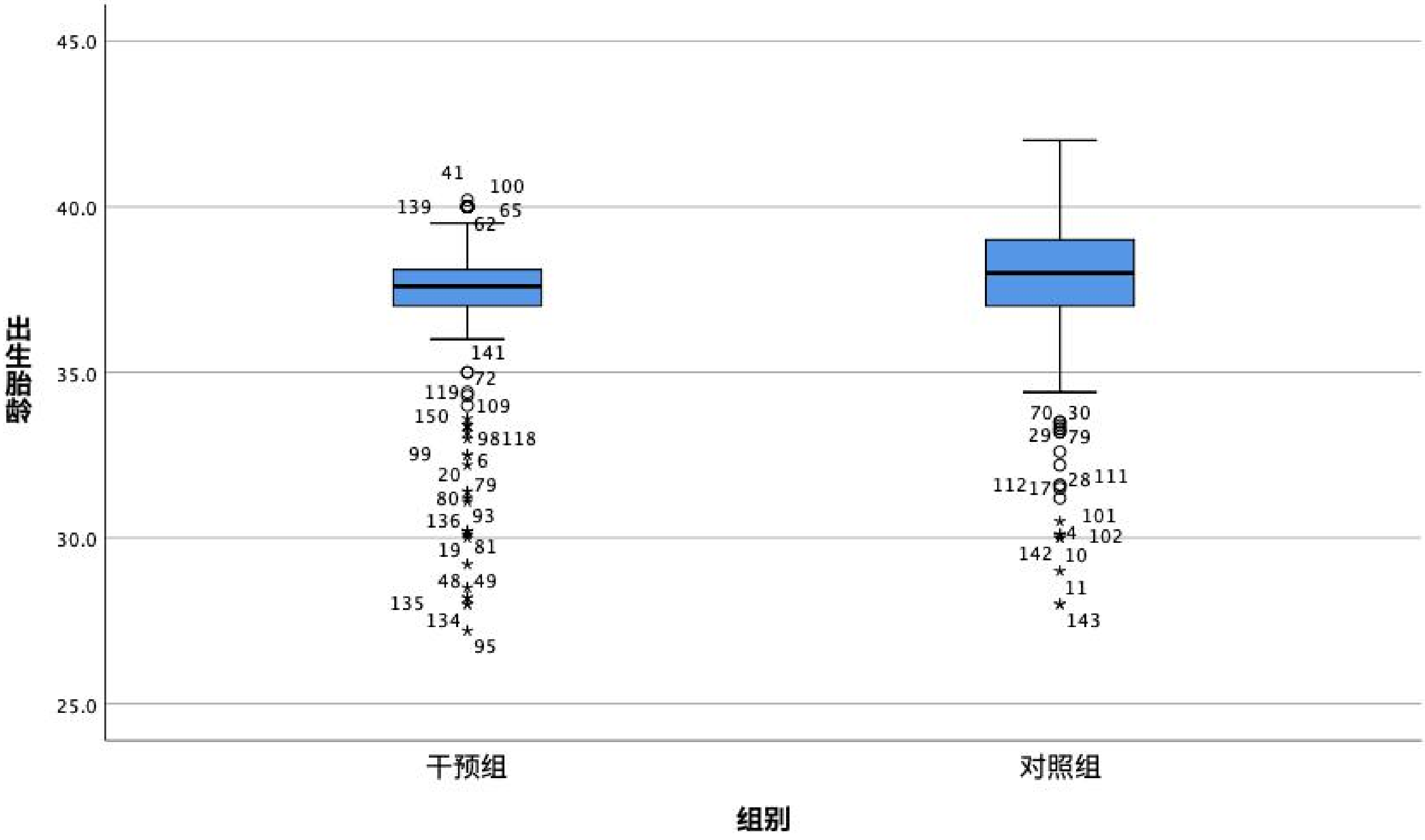

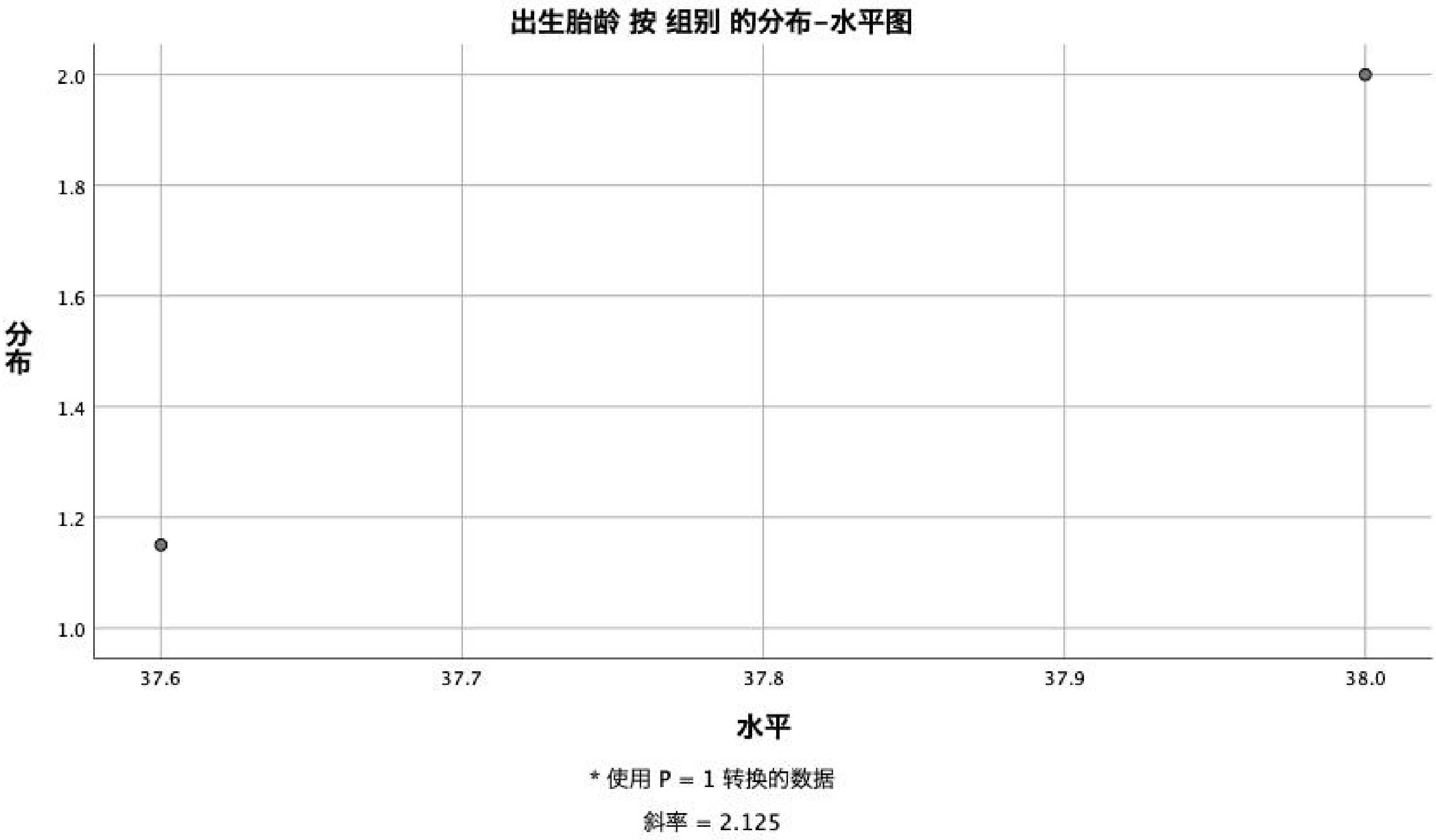


```
EXAMINE VARIABLES=出生胎龄
  /ID=患者编号
  /PLOT BOXPLOT STEMLEAF HISTOGRAM NPPLOT SPREADLEVEL(1)
```

```
/COMPARE GROUPS
/MESTIMATORS HUBER(1.339) ANDREW(1.34) HAMPEL(1.7,3.4,8.5) TUKEY(4.685)
/PERCENTILES(5,10,25,50,75,90,95) HAVERAGE
/STATISTICS DESCRIPTIVES EXTREME
/CINTERVAL 95
/MISSING LISTWISE
/NOTOTAL.
```

探索

备注

已创建输出		27-JUN-2022 10:24:38
注释		
输入	数据	/Users/jasmine/Documents/工作/我的科研课题/GDD早期家庭干预队列/GDD 数据集.sav
	活动数据集	数据集1
	过滤器	<无>
	权重	<无>
	拆分文件	<无>
	工作数据文件中的行数	306
缺失值处理	对缺失的定义	将因变量的用户定义缺失值视为缺失。
	使用的个案数	统计基于那些对任何所用因变量或因子都没有缺失值的个案。

语法		EXAMINE VARIABLES=出生胎龄<br>/ID=患者编号<br>/PLOT BOXPLOT STEMLEAF HISTOGRAM NPPLOT SPREADLEVEL(1)<br>/COMPARE GROUPS<br>/MESTIMATORS HUBER(1.339) ANDREW(1.34) HAMPEL(1.7,3.4,8.5) TUKEY(4.685)<br>/PERCENTILES(5,10,25,50,75,90,95) HAVERAGE<br>/STATISTICS DESCRIPTIVES EXTREME<br>/CINTERVAL 95<br>/MISSING LISTWISE<br>/NOTOTAL.
资源	处理程序时间	00:00:00.56
	耗用时间	00:00:00.00

警告

已请求生成分布-水平图，但未指定因子变量。将不会生成分布-水平图。

个案处理摘要

	个案					
	有效		缺失		总计	
	N	百分比	N	百分比	N	百分比
出生胎龄	306	100.0%	0	0.0%	306	100.0%

描述

			统计	标准误差
出生胎龄	平均值		36.921	.1656
	平均值的 95% 置信区间	下限	36.595	
		上限	37.247	
	5% 剪除后平均值		37.169	
	中位数		38.000	
	方差		8.391	
	标准偏差		2.8967	

	最小值	27.2	
	最大值	42.0	
	范围	14.8	
	四分位距	2.0	
	偏度	-1.501	.139
	峰度	1.751	.278

M 估计量

	休伯 M 估计量[a]	图基双权[b]	汉佩尔 M 估计量[c]	安德鲁波[d]
出生胎龄	37.716	38.037	37.912	38.038

a. 加权常量为 1.339。
b. 加权常量为 4.685。
c. 加权常量为 1.700、3.400 和 8.500
d. 加权常量为 1.340*pi。

百分位数

		百分位数						
		5	10	25	50	75	90	95
加权平均（定义 1）	出生胎龄	30.100	32.200	37.000	38.000	39.000	39.330	40.000
图基枢纽	出生胎龄			37.000	38.000	39.000		

极值

			个案号	患者编号	值
出生胎龄	最大值	1	169	16	42.0
		2	167	14	41.0
		3	198	45	41.0
		4	240	87	41.0
		5	246	93	41.0[a]
	最小值	1	95	95	27.2
		2	296	143	28.0
		3	164	11	28.0
		4	135	135	28.0
		5	134	134	28.0

a. 在较大极值的表中，仅显示了不完整的个案列表（这些个案的值为 41.0）。

正态性检验

	柯尔莫戈洛夫-斯米诺夫(V)[a]			夏皮洛-威尔克		
	统计	自由度	显著性	统计	自由度	显著性
出生胎龄	.276	306	.000	.827	306	.000

a. 里利氏显著性修正

出生胎龄

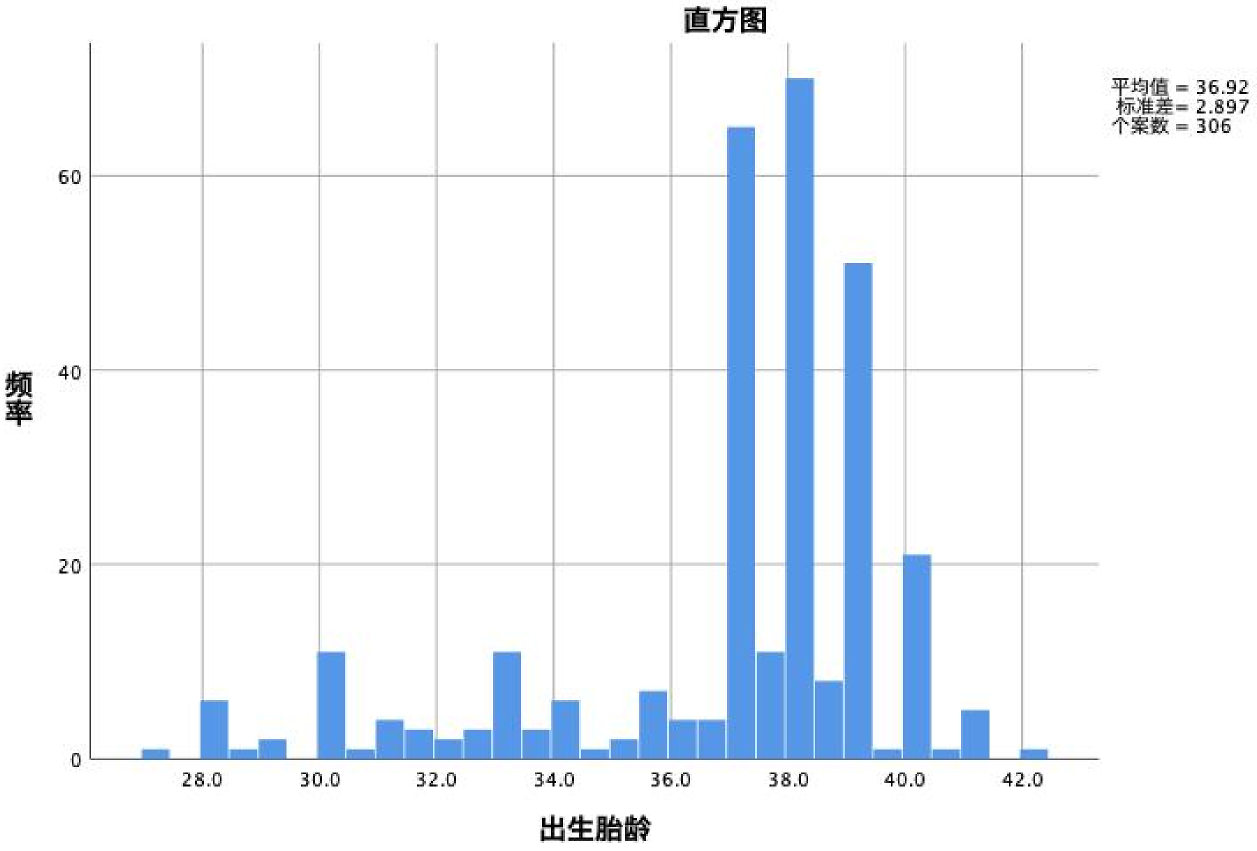

出生胎龄 茎叶图

```
 频率        Stem &   叶

   48.00 Extremes    (=<33.6)
    6.00        34 .  034444
    1.00        34 .  5
    2.00        35 .  00
    7.00        35 .  5555566
    4.00        36 .  0133
    4.00        36 .  5556
   65.00        37 .  00000000000000000000000000000000000111111112222222222223333333444
   11.00        37 .  55556666666
```

70.00	38 .	00000000000000000000000000000000000000000000000000111111122222222223444
8.00	38 .	55555556
51.00	39 .	0000000000000000000000000000000000111111111222222234
1.00	39 .	5
21.00	40 .	000000000000000000012
1.00	40 .	5
5.00	41 .	00000
.00	41 .	
1.00	42 .	0

主干宽度： 1.0
每个叶： 1 个案

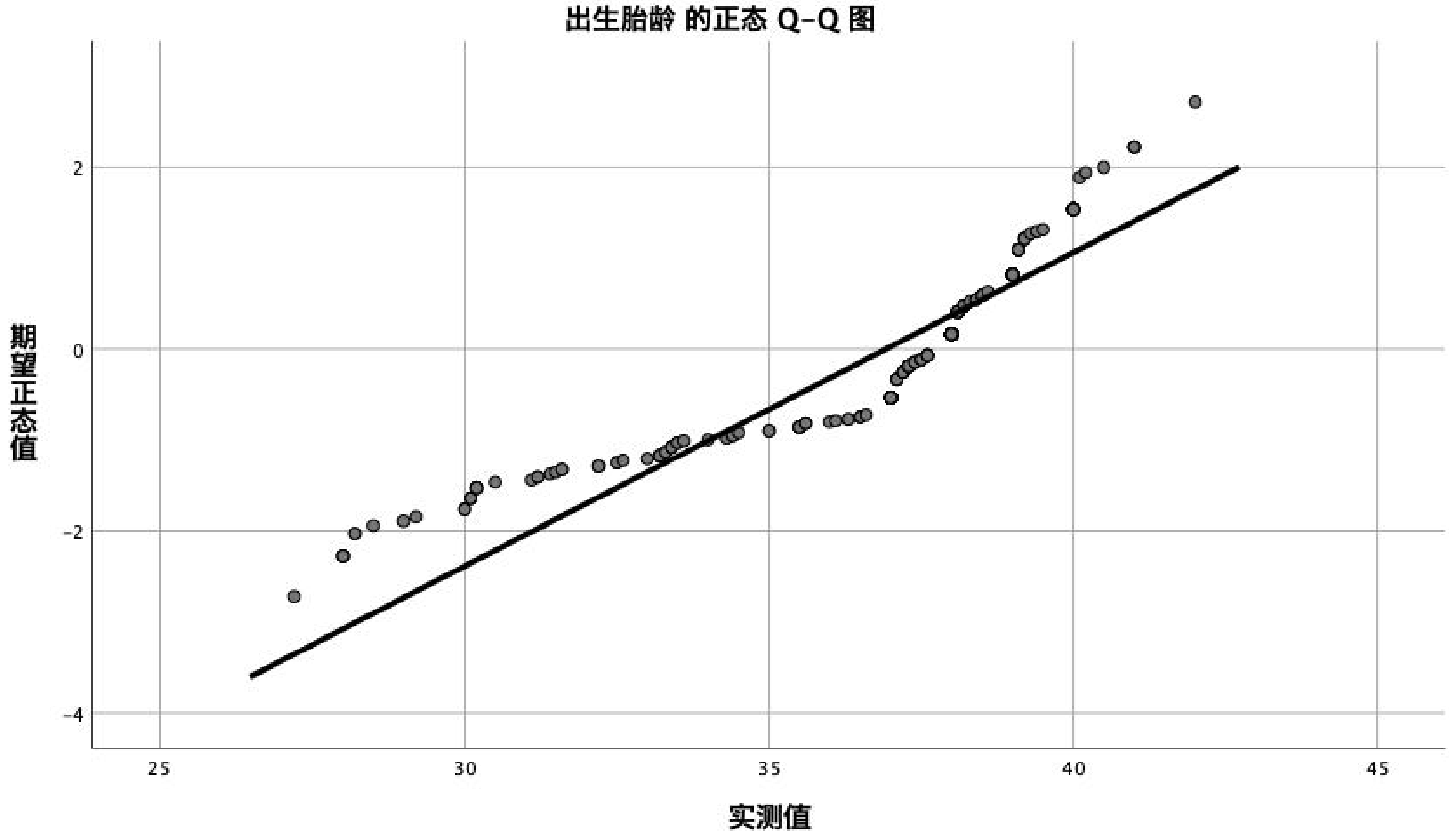

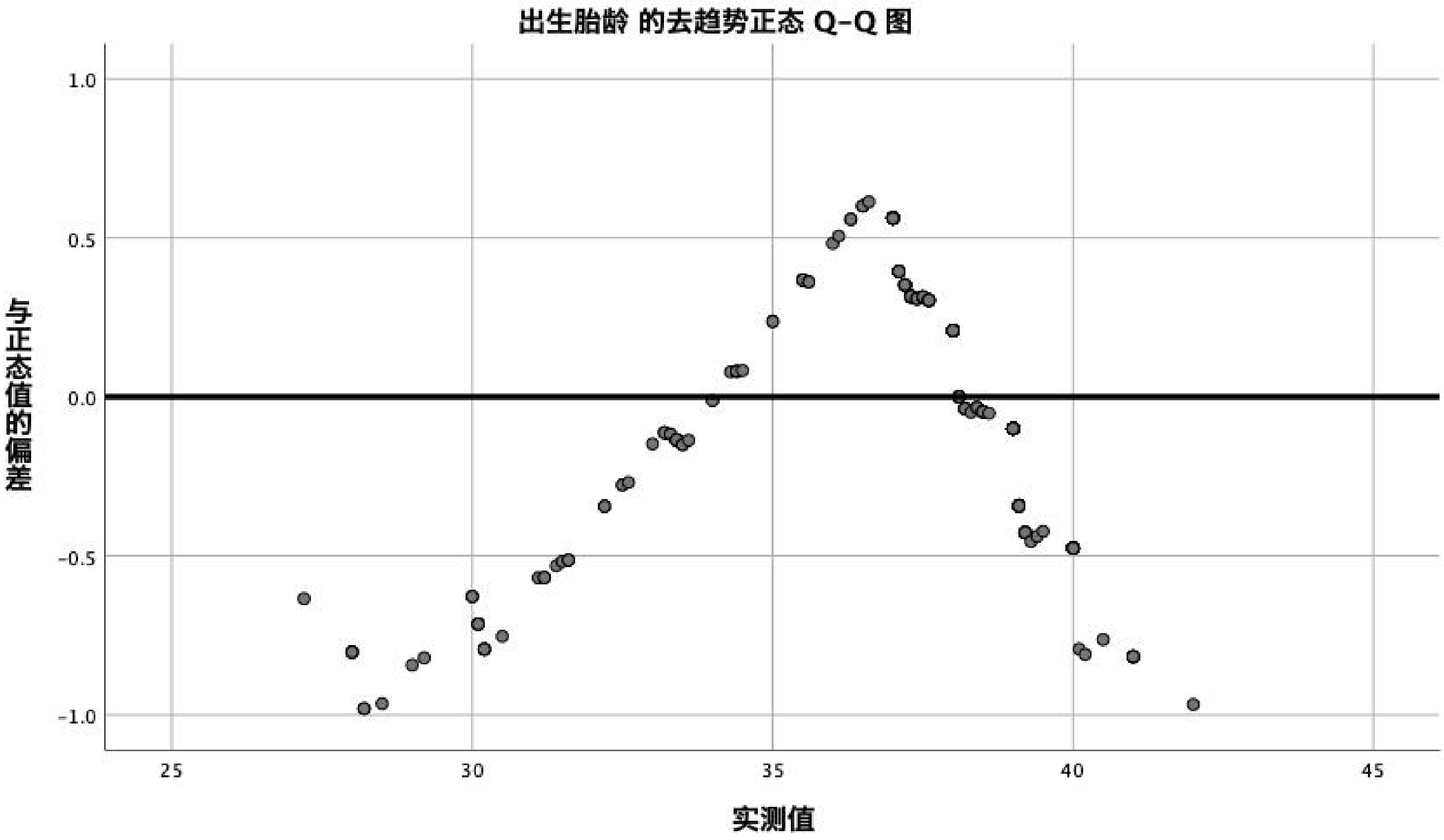

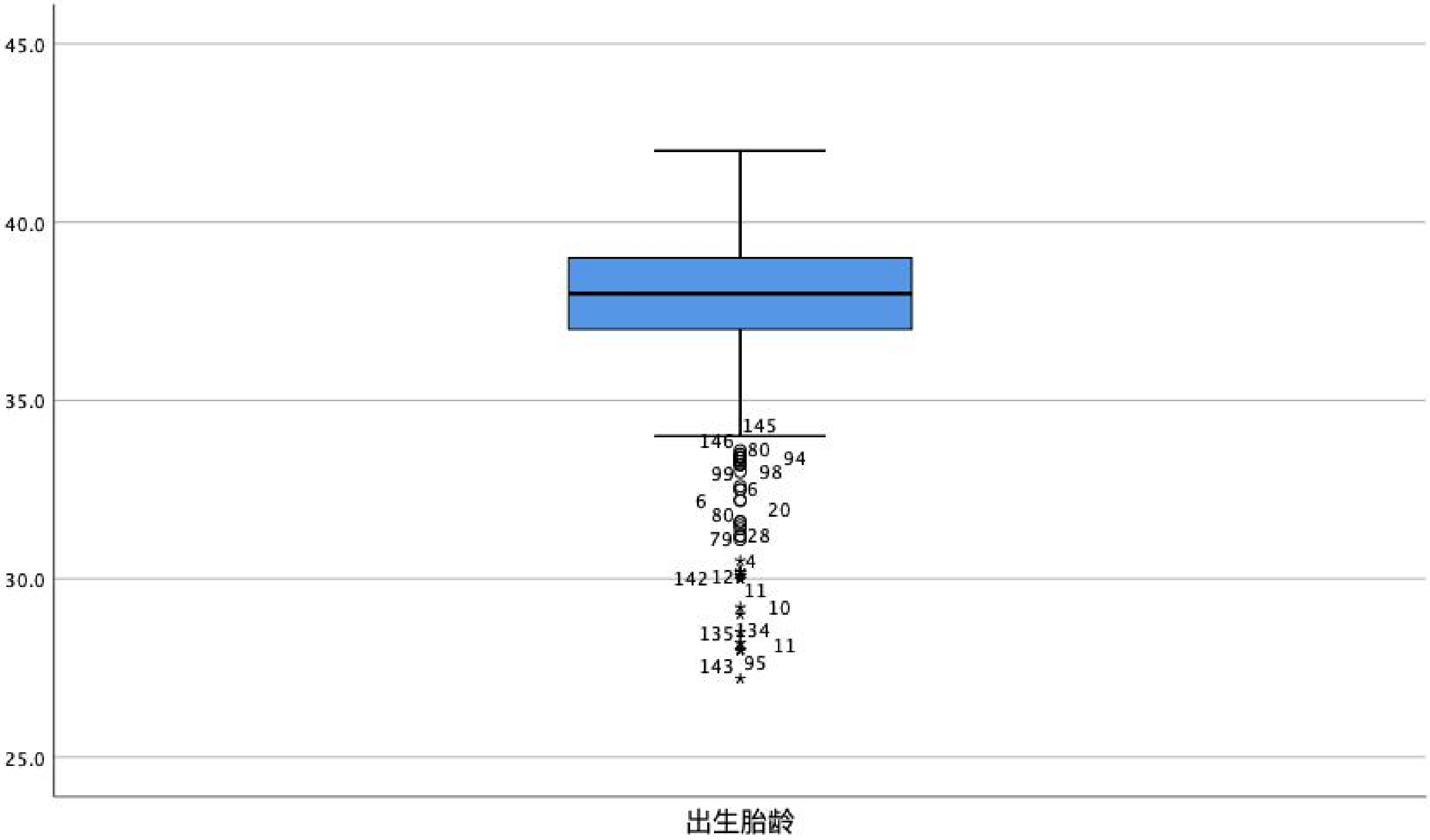

NPAR TESTS
/M-W= 出生胎龄 BY 组别(1 2)
/STATISTICS=DESCRIPTIVES QUARTILES

/MISSING ANALYSIS.

NPar 检验

备注

已创建输出		27-JUN-2022 10:51:59
注释		
输入	数据	/Users/jasmine/Documents/工作/我的科研课题/GDD早期家庭干预队列/GDD 数据集.sav
	活动数据集	数据集1
	过滤器	<无>
	权重	<无>
	拆分文件	<无>
	工作数据文件中的行数	306
缺失值处理	对缺失的定义	将用户定义的缺失值视为缺失。
	使用的个案数	每项检验的统计都基于所有对于该检验中使用的变量具有有效数据的个案。
语法		NPAR TESTS<br>/M-W= 出生胎龄 BY 组别(1 2)<br><br>/STATISTICS=DESCRIPTIVES QUARTILES<br>/MISSING ANALYSIS.
资源	处理程序时间	00:00:00.01
	耗用时间	00:00:00.00
	允许的个案数[a]	449389

a. 基于工作空间内存的可用性。

描述统计

	个案数	平均值	标准 偏差	最小值	最大值	百分位数		
						第 25 个	第 50 个(中位数)	第 75 个
出生胎龄	306	36.921	2.8967	27.2	42.0	37.000	38.000	39.000

组别	306	1.50	.501	1	2	1.00	1.50	2.00

曼-惠特尼检验

秩

	组别	个案数	秩平均值	秩的总和
出生胎龄	干预组	153	142.70	21833.50
	对照组	153	164.30	25137.50
	总计	306		

检验统计[a]

	出生胎龄
曼-惠特尼 U	10052.500
威尔科克森 W	21833.500
Z	-2.143
渐近显著性（双尾）	.032

a. 分组变量：组别

```
EXAMINE VARIABLES=出生体重 BY 组别
  /ID=患者编号
  /PLOT BOXPLOT STEMLEAF HISTOGRAM NPPLOT SPREADLEVEL(1)
  /COMPARE GROUPS
  /MESTIMATORS HUBER(1.339) ANDREW(1.34) HAMPEL(1.7,3.4,8.5) TUKEY(4.685)
  /PERCENTILES(5,10,25,50,75,90,95) HAVERAGE
  /STATISTICS DESCRIPTIVES EXTREME
  /CINTERVAL 95
  /MISSING LISTWISE
  /NOTOTAL.
```

探索

备注

已创建输出	27-JUN-2022 11:36:17

注释		
输入	数据	/Users/jasmine/Documents/工作/我的科研课题/GDD早期家庭干预队列/GDD 数据集.sav
	活动数据集	数据集1
	过滤器	<无>
	权重	<无>
	拆分文件	<无>
	工作数据文件中的行数	306
缺失值处理	对缺失的定义	将因变量的用户定义缺失值视为缺失。
	使用的个案数	统计基于那些对任何所用因变量或因子都没有缺失值的个案。
语法		EXAMINE VARIABLES=出生体重 BY 组别<br>/ID=患者编号<br>/PLOT BOXPLOT STEMLEAF HISTOGRAM NPPLOT SPREADLEVEL(1)<br>/COMPARE GROUPS<br>/MESTIMATORS HUBER(1.339) ANDREW(1.34) HAMPEL(1.7,3.4,8.5) TUKEY(4.685)<br>/PERCENTILES(5,10,25,50,75,90,95) HAVERAGE<br>/STATISTICS DESCRIPTIVES EXTREME<br>/CINTERVAL 95<br>/MISSING LISTWISE<br>/NOTOTAL.
资源	处理程序时间	00:00:01.06
	耗用时间	00:00:01.00

组别

个案处理摘要

组别	个案

		有效		缺失		总计	
		N	百分比	N	百分比	N	百分比
出生体重	干预组	153	100.0%	0	0.0%	153	100.0%
	对照组	153	100.0%	0	0.0%	153	100.0%

描述

	组别			统计	标准误差
出生体重	干预组	平均值		2681.65	64.298
		平均值的 95% 置信区间	下限	2554.61	
			上限	2808.68	
		5% 剪除后平均值		2707.89	
		中位数		2660.00	
		方差		632528.611	
		标准偏差		795.317	
		最小值		650	
		最大值		4235	
		范围		3585	
		四分位距		698	
		偏度		-.490	.196
		峰度		.326	.390
	对照组	平均值		2748.58	61.340
		平均值的 95% 置信区间	下限	2627.39	
			上限	2869.77	
		5% 剪除后平均值		2770.98	
		中位数		2680.00	
		方差		575668.113	
		标准偏差		758.728	
		最小值		795	
		最大值		4300	
		范围		3505	
		四分位距		819	
		偏度		-.298	.196
		峰度		.099	.390

M 估计量

	组别	休伯 M 估计量[a]	图基双权[b]	汉佩尔 M 估计量[c]	安德鲁波[d]
出生体重	干预组	2730.69	2770.70	2748.02	2772.33
	对照组	2768.81	2794.20	2776.94	2798.82

a. 加权常量为 1.339。

b. 加权常量为 4.685。

c. 加权常量为 1.700、3.400 和 8.500

d. 加权常量为 1.340*pi。

百分位数

		组别	百分位数 5	10	25	50	75	90	
加权平均（定义 1）	出生体重	干预组	981.50	1466.00	2430.00	2660.00	3127.50	3760.00	
		对照组	1113.50	1716.00	2445.00	2680.00	3264.00	3944.00	
图基枢组	出生体重	干预组			2430.00	2660.00	3125.00		
		对照组			2450.00	2680.00	3228.00		

极值

	组别			个案号	患者编号	值
出生体重	干预组	最大值	1	4	4	4235
			2	45	45	4200
			3	36	36	4020
			4	77	77	4020
			5	100	100	4020
		最小值	1	95	95	650
			2	134	134	702
			3	17	17	755
			4	135	135	825
			5	81	81	855
	对照组	最大值	1	279	126	4300
			2	280	127	4105
			3	252	99	4100
			4	266	113	4100
			5	208	55	4070
		最小值	1	210	57	795
			2	296	143	934
			3	164	11	995
			4	254	101	1005
			5	163	10	1020

正态性检验

		柯尔莫戈洛夫-斯米诺夫(V)[a]			夏皮洛-威尔克		
	组别	统计	自由度	显著性	统计	自由度	显著性
出生体重	干预组	.134	153	.000	.951	153	.000

	对照组	.104	153	.000	.970	153	.002

a. 里利氏显著性修正

方差齐性检验

		莱文统计	自由度 1	自由度 2	显著性
出生体重	基于平均值	.075	1	304	.784
	基于中位数	.082	1	304	.775
	基于中位数并具有调整后自由度	.082	1	302.859	.775
	基于剪除后平均值	.084	1	304	.772

出生体重

直方图(O)

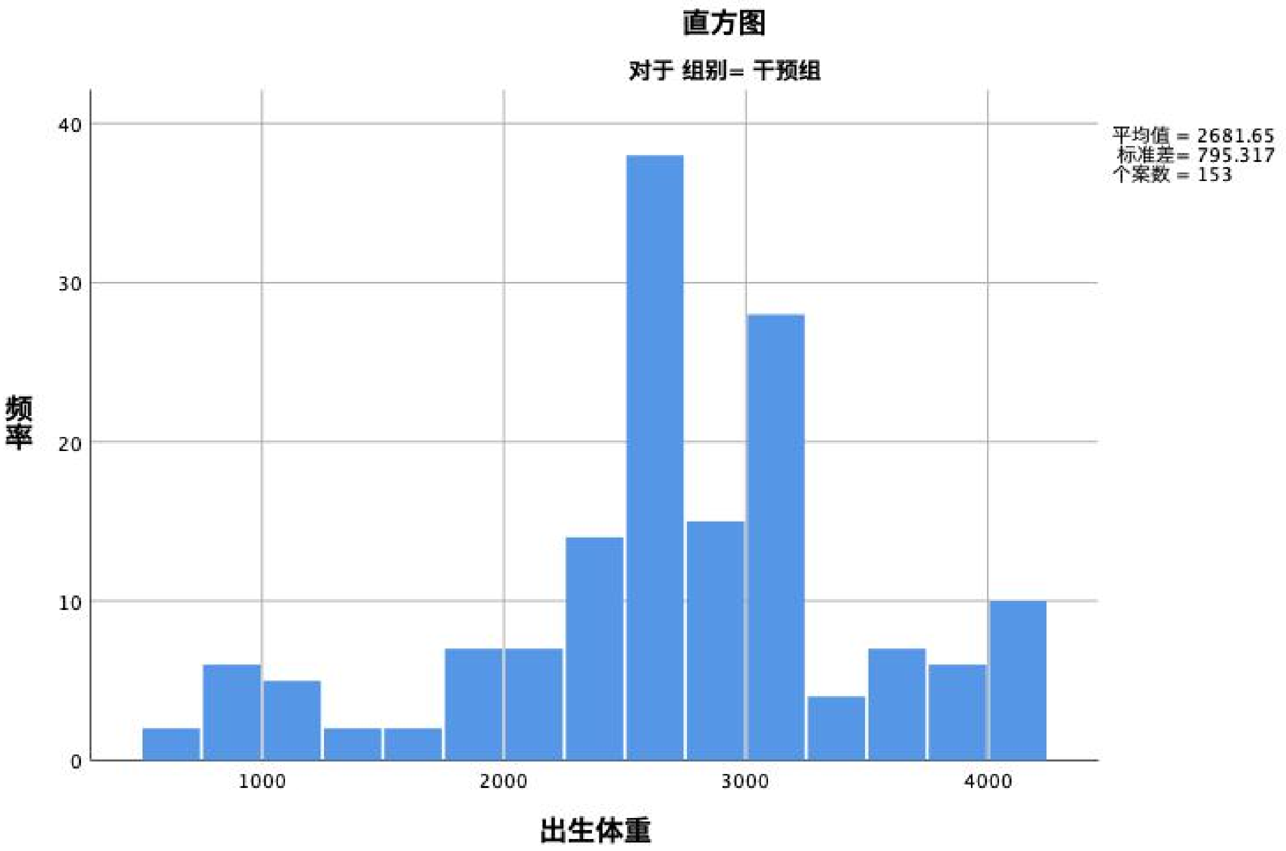

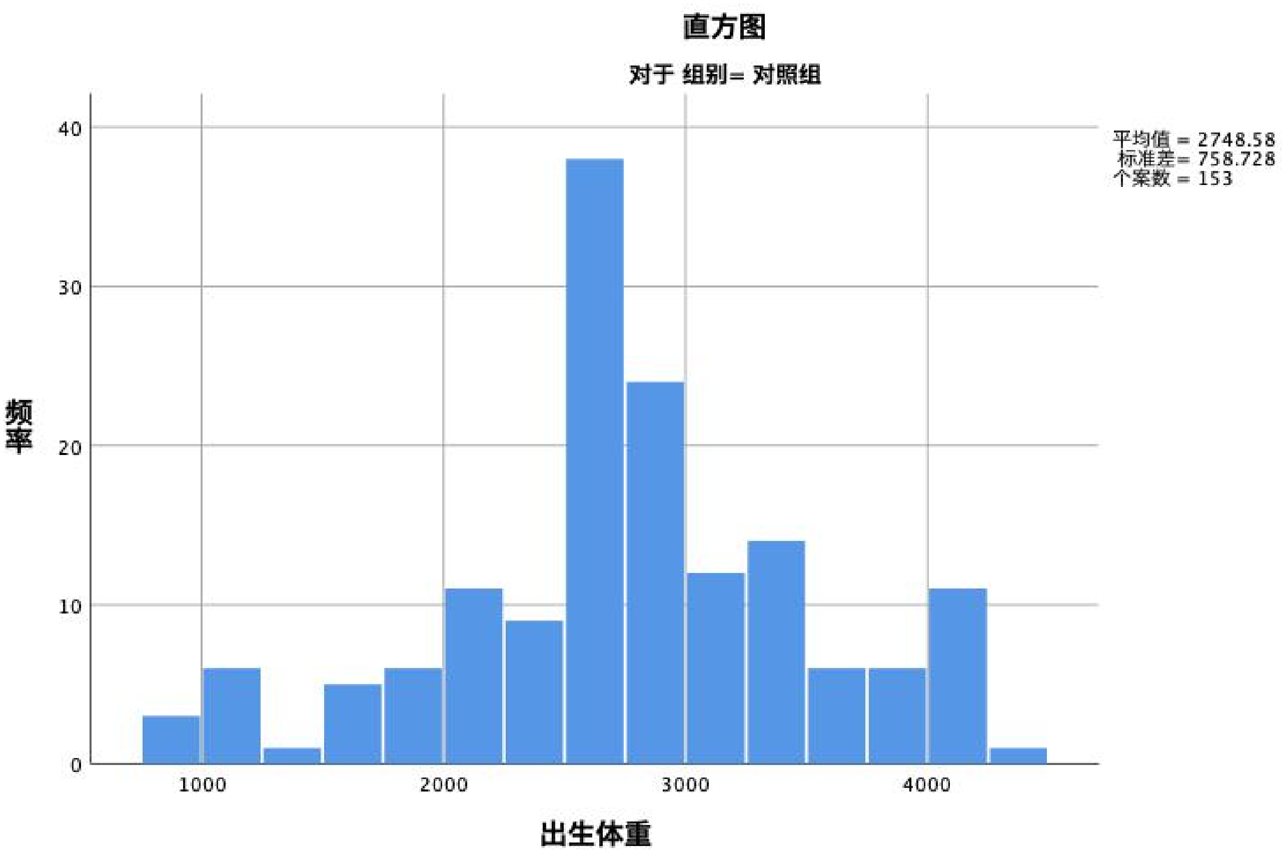

茎叶图

出生体重 茎叶图：
组别= 干预组

频率	Stem &	叶
15.00	Extremes	(=<1330)
3.00	1 .	667
6.00	1 .	888999
6.00	2 .	000000
6.00	2 .	233333
34.00	2 .	4444444445555555555555555555555555
18.00	2 .	666666666666677777
10.00	2 .	8899999999
21.00	3 .	000000000000111111111
10.00	3 .	2222222333
3.00	3 .	455
7.00	3 .	7777777
4.00	3 .	8999
8.00	4 .	00000000
2.00	极值	(>=4200)

主干宽度：	1000
每个叶：	1 个案

出生体重 茎叶图：
组别= 对照组

频率	Stem &	叶
9.00 Extremes		(=<1219)
1.00	1 .	3
2.00	1 .	55
5.00	1 .	66677
4.00	1 .	8889
9.00	2 .	000000011
4.00	2 .	2233
27.00	2 .	444444455555555555555555555
23.00	2 .	66666666666666677777777
19.00	2 .	8888889999999999999
9.00	3 .	000001111
13.00	3 .	2223333333333
6.00	3 .	444455
5.00	3 .	66677
5.00	3 .	88999
11.00	4 .	00000000111
1.00	4 .	3

主干宽度：	1000
每个叶：	1 个案

正态 Q-Q 图

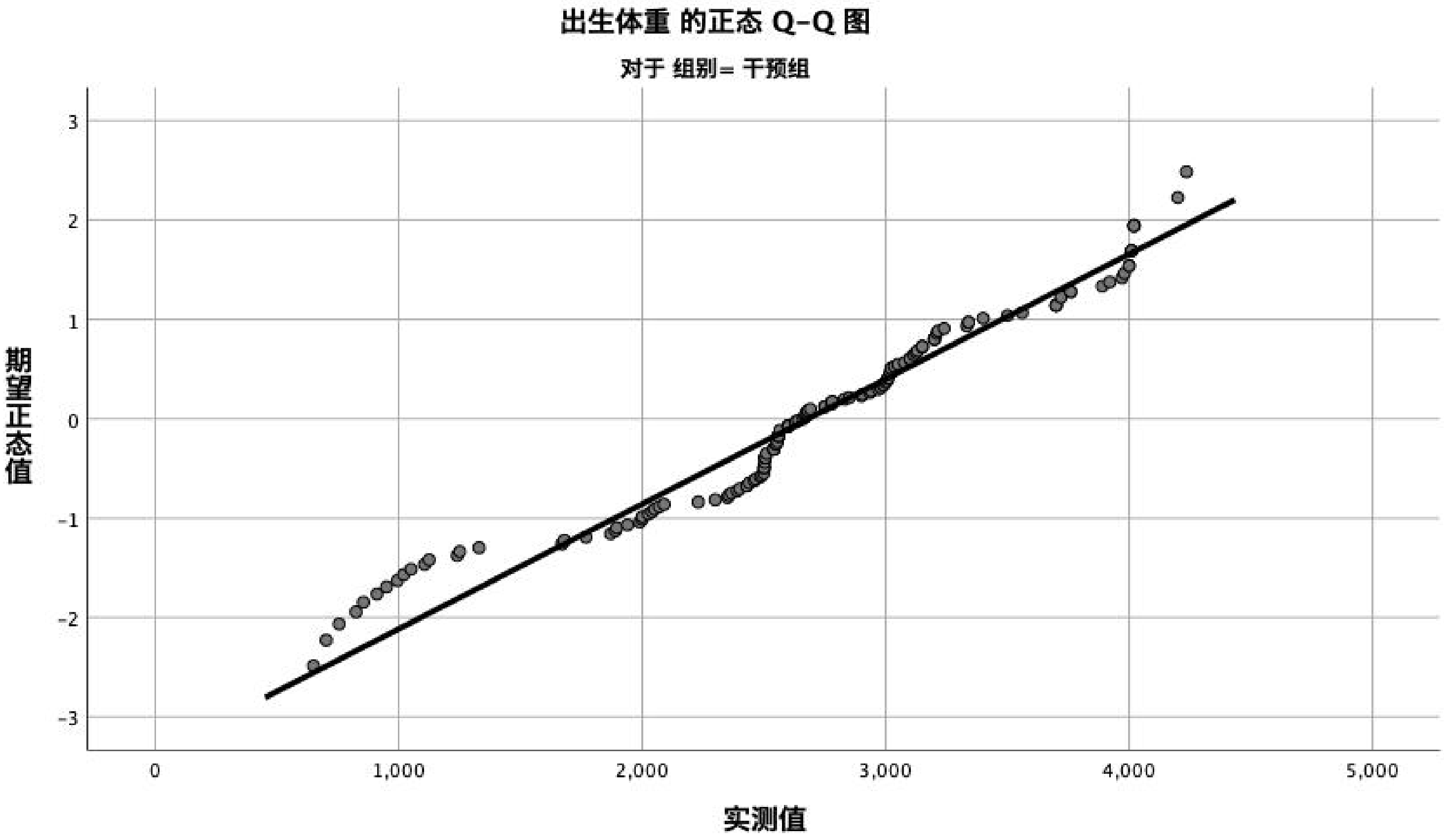
出生体重 的正态 Q-Q 图
对于 组别= 干预组
期望正态值
实测值
3
2
1
0
-1
-2
-3
0
1,000
2,000
3,000
4,000
5,000

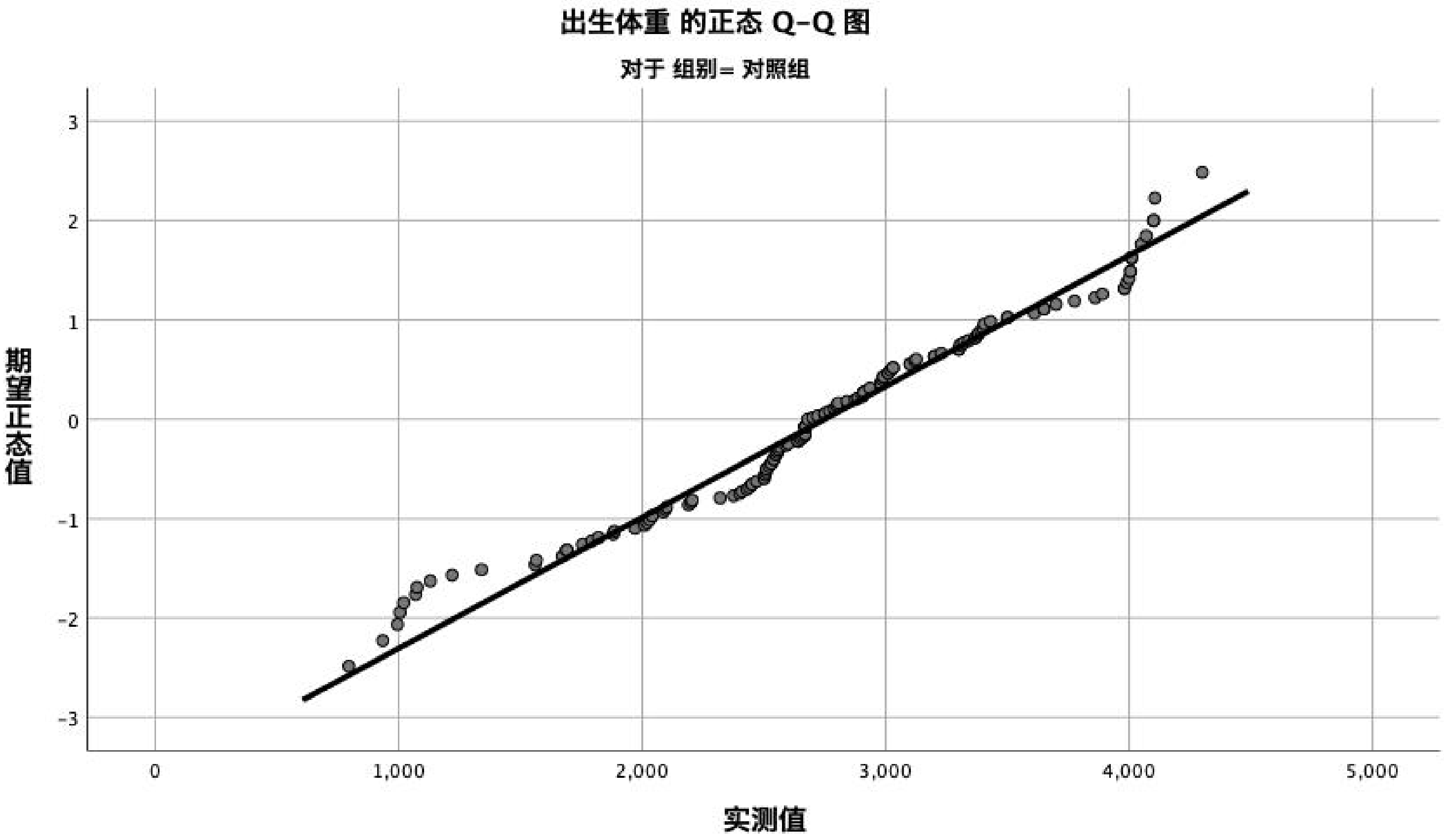
出生体重 的正态 Q-Q 图
对于 组别= 对照组
期望正态值
实测值
3
2
1
0
-1
-2
-3
0
1,000
2,000
3,000
4,000
5,000

去趋势正态 Q-Q 图

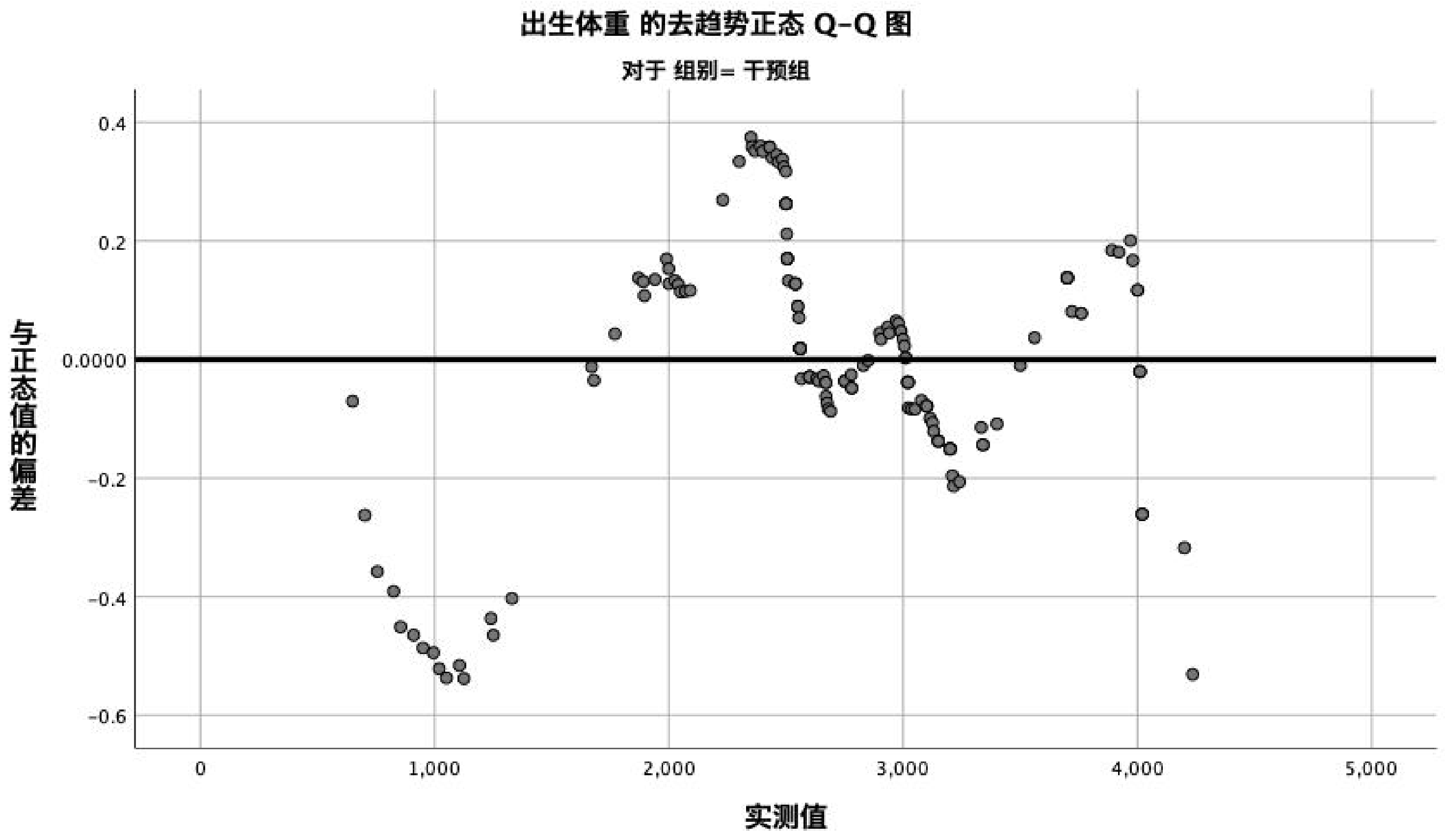
出生体重 的去趋势正态 Q-Q 图
对于 组别= 干预组
与正态值的偏差
0.4
0.2
0.0000
-0.2
-0.4
-0.6
0
1,000
2,000
3,000
4,000
5,000
实测值

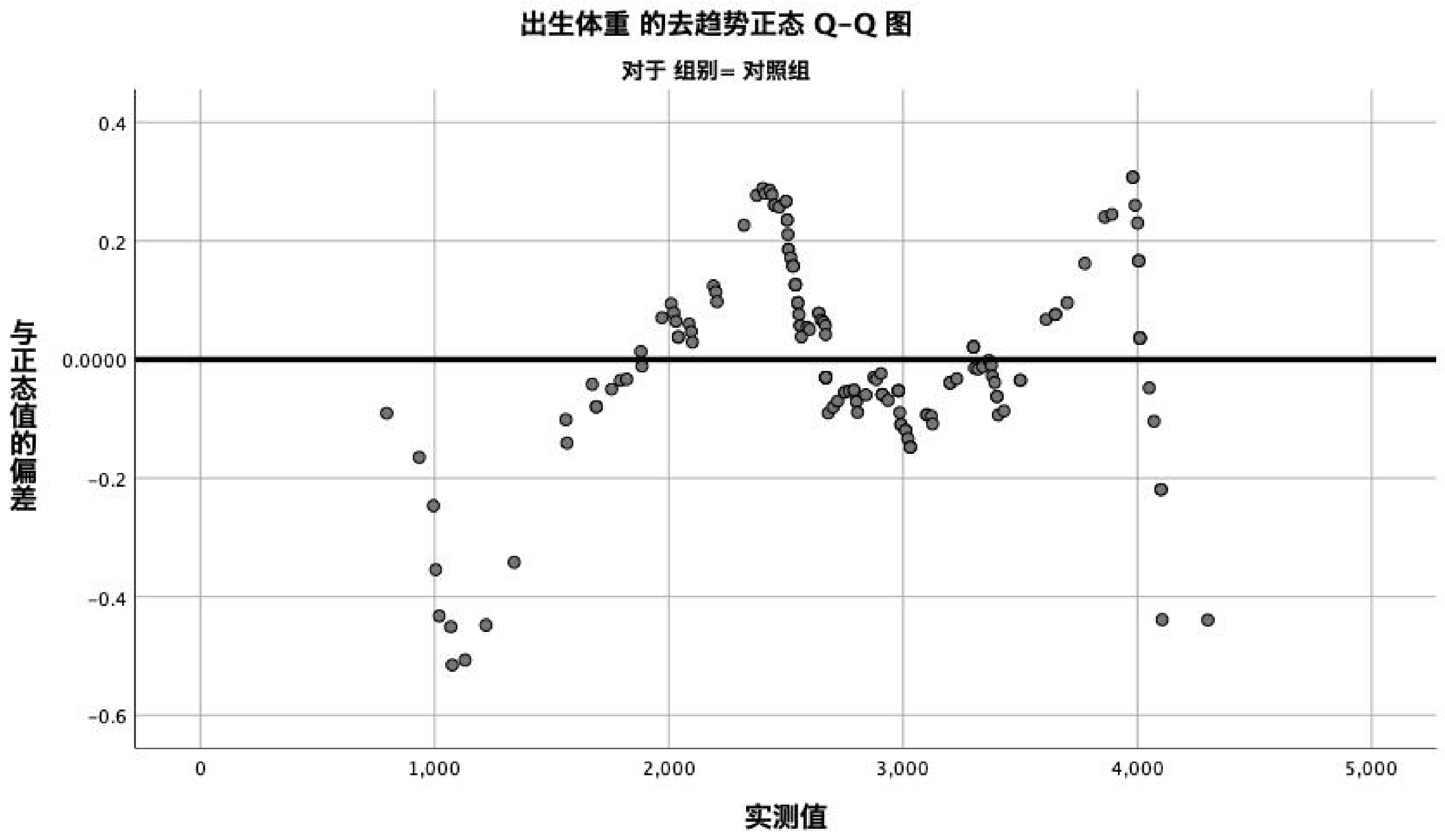
出生体重 的去趋势正态 Q-Q 图
对于 组别= 对照组
与正态值的偏差
0.4
0.2
0.0000
-0.2
-0.4
-0.6
0
1,000
2,000
3,000
4,000
5,000
实测值

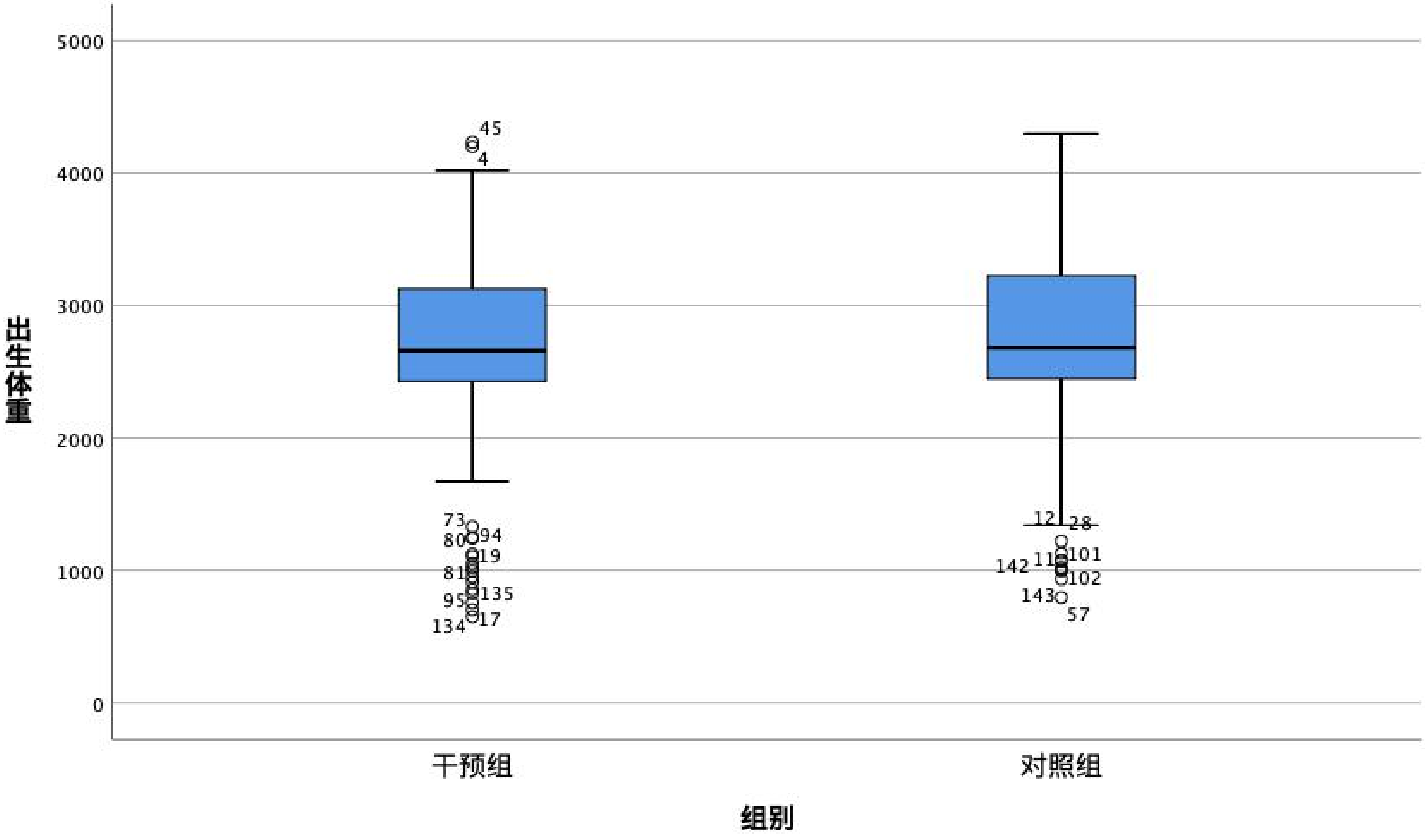

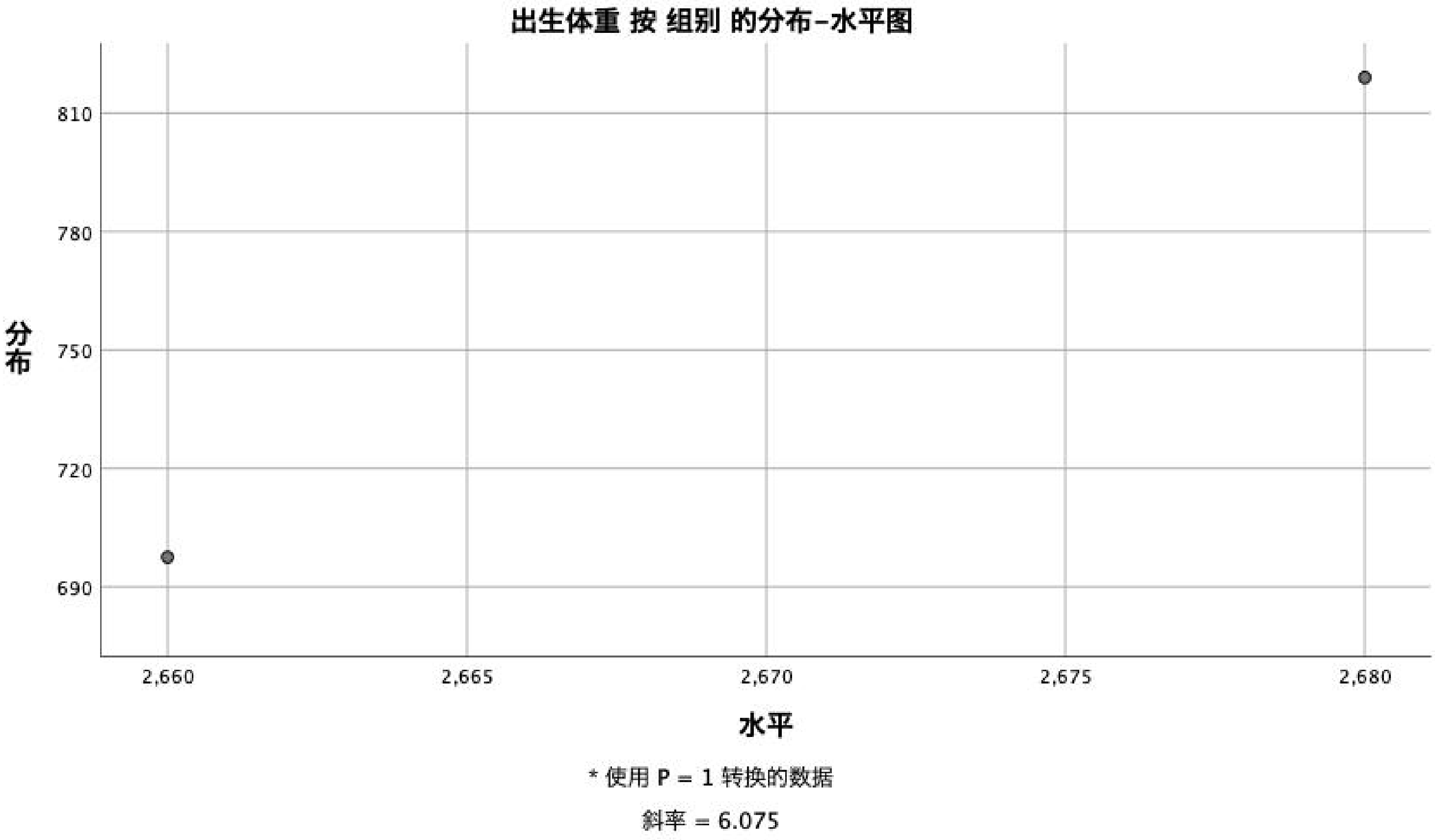


```
EXAMINE VARIABLES=出生体重
  /ID=患者编号
  /PLOT BOXPLOT STEMLEAF HISTOGRAM NPPLOT SPREADLEVEL(1)
```

```
/COMPARE GROUPS
/MESTIMATORS HUBER(1.339) ANDREW(1.34) HAMPEL(1.7,3.4,8.5) TUKEY(4.685)
/PERCENTILES(5,10,25,50,75,90,95) HAVERAGE
/STATISTICS DESCRIPTIVES EXTREME
/CINTERVAL 95
/MISSING LISTWISE
/NOTOTAL.
```

探索

备注

已创建输出		27-JUN-2022 11:43:21
注释		
输入	数据	/Users/jasmine/Documents/工作/我的科研课题/GDD早期家庭干预队列/GDD 数据集.sav
	活动数据集	数据集1
	过滤器	<无>
	权重	<无>
	拆分文件	<无>
	工作数据文件中的行数	306
缺失值处理	对缺失的定义	将因变量的用户定义缺失值视为缺失。
	使用的个案数	统计基于那些对任何所用因变量或因子都没有缺失值的个案。

语法		EXAMINE VARIABLES=出生体重<br>/ID=患者编号<br>/PLOT BOXPLOT STEMLEAF HISTOGRAM NPPLOT SPREADLEVEL(1)<br>/COMPARE GROUPS<br>/MESTIMATORS HUBER(1.339) ANDREW(1.34) HAMPEL(1.7,3.4,8.5) TUKEY(4.685)<br>/PERCENTILES(5,10,25,50,75,90,95) HAVERAGE<br>/STATISTICS DESCRIPTIVES EXTREME<br>/CINTERVAL 95<br>/MISSING LISTWISE<br>/NOTOTAL.
资源	处理程序时间	00:00:00.64
	耗用时间	00:00:01.00

警告

已请求生成分布-水平图，但未指定因子变量。将不会生成分布-水平图。

个案处理摘要

	个案					
	有效		缺失		总计	
	N	百分比	N	百分比	N	百分比
出生体重	306	100.0%	0	0.0%	306	100.0%

描述

			统计	标准误差
出生体重	平均值		2715.11	44.400
	平均值的 95% 置信区间	下限	2627.74	
		上限	2802.48	
	5% 剪除后平均值		2739.94	
	中位数		2670.00	
	方差		603241.446	
	标准偏差		776.686	

	最小值	650	
	最大值	4300	
	范围	3650	
	四分位距	713	
	偏度	-.404	.139
	峰度	.228	.278

M 估计量

	休伯 M 估计量[a]	图基双权[b]	汉佩尔 M 估计量[c]	安德鲁波[d]
出生体重	2747.26	2776.94	2765.52	2778.20

a. 加权常量为 1.339。
b. 加权常量为 4.685。
c. 加权常量为 1.700、3.400 和 8.500
d. 加权常量为 1.340*pi。

百分位数

		百分位数						
		5	10	25	50	75	90	95
加权平均(定义 1)	出生体重	1056.65	1687.00	2437.50	2670.00	3150.00	3869.00	4010.00
图基枢纽	出生体重			2440.00	2670.00	3150.00		

极值

			个案号	患者编号	值
出生体重	最大值	1	279	126	4300
		2	4	4	4235
		3	45	45	4200
		4	280	127	4105
		5	252	99	4100[a]
	最小值	1	95	95	650
		2	134	134	702
		3	17	17	755
		4	210	57	795
		5	135	135	825

a. 在较大极值的表中，仅显示了不完整的个案列表（这些个案的值为 4100）。

正态性检验

	柯尔莫戈洛夫-斯米诺夫(V)[a]			夏皮洛-威尔克		
	统计	自由度	显著性	统计	自由度	显著性
出生体重	.118	306	.000	.963	306	.000

a. 里利氏显著性修正

出生体重

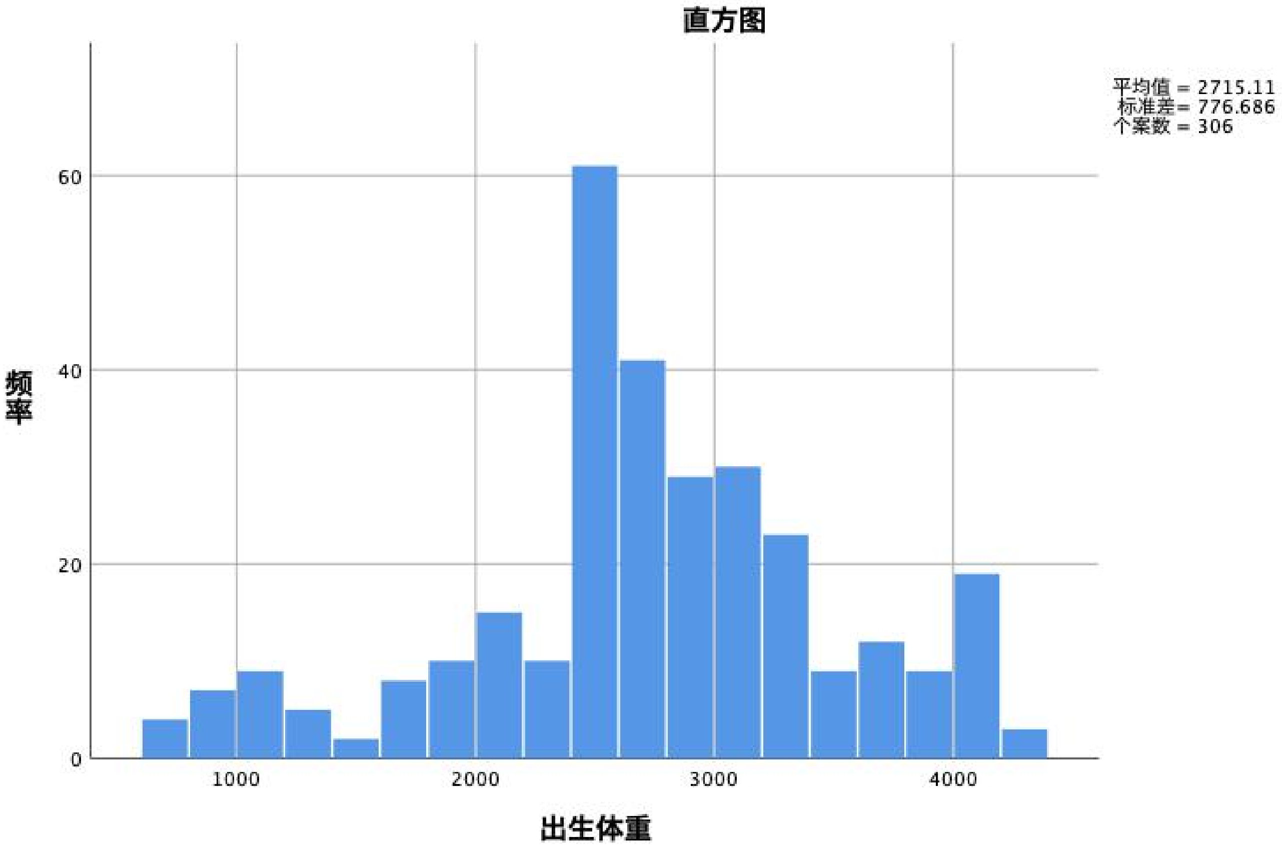

出生体重 茎叶图

频率	Stem &	叶
25.00 Extremes		(=<1340)
2.00	1 .	55
8.00	1 .	66666777
10.00	1 .	8888889999
15.00	2 .	000000000000011
10.00	2 .	2223333333
61.00	2 .	4444444444444444555555555555555555555555555555555555555555555
41.00	2 .	66666666666666666666666666666777777777777
29.00	2 .	88888888999999999999999999999

30.00	3 .	000000000000000001111111111111
23.00	3 .	22222222223333333333333
9.00	3 .	444445555
12.00	3 .	666777777777
9.00	3 .	888999999
19.00	4 .	0000000000000000111
1.00	4 .	2
2.00 极值		(>=4235)

主干宽度： 1000
每个叶： 1 个案

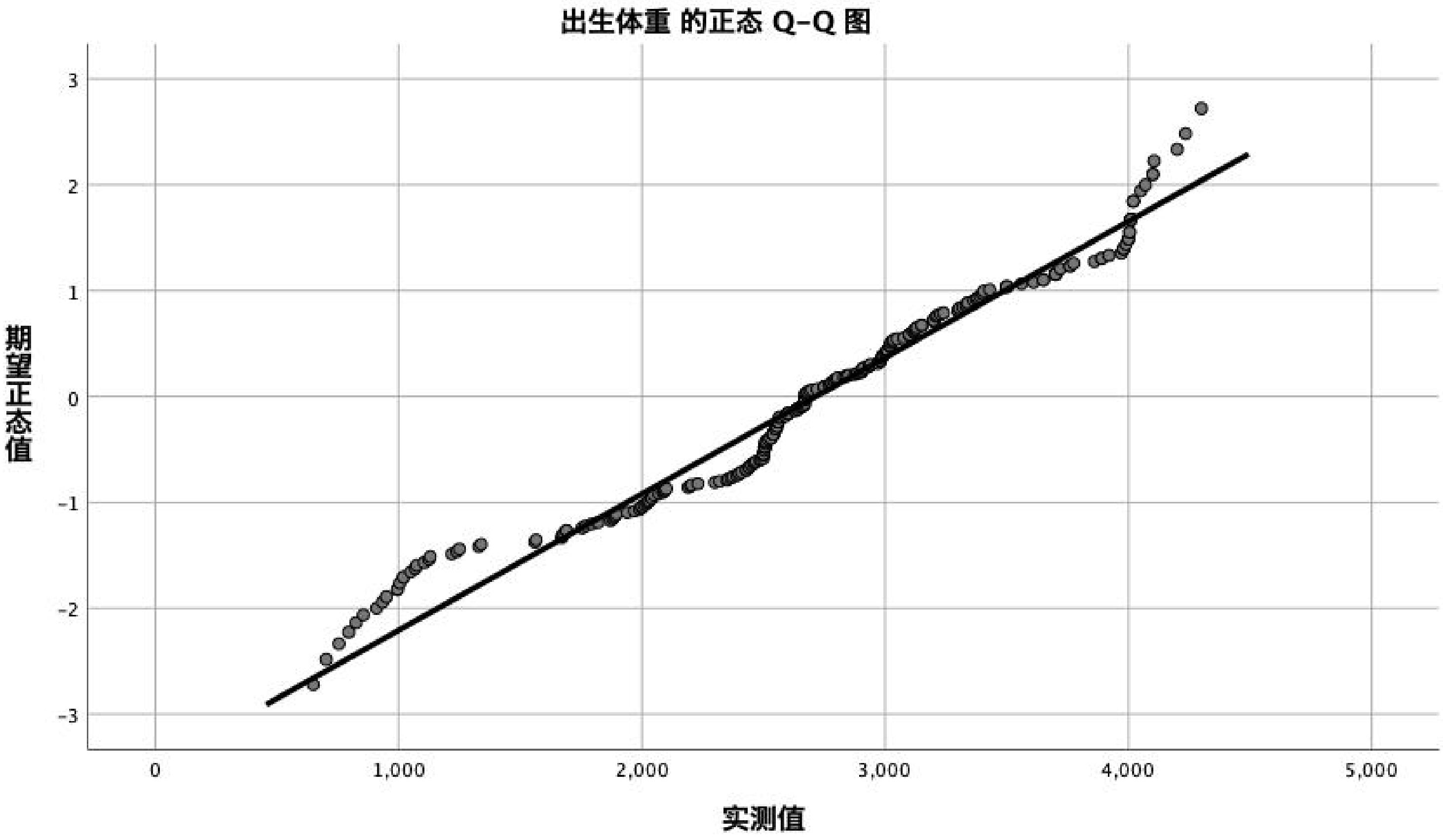

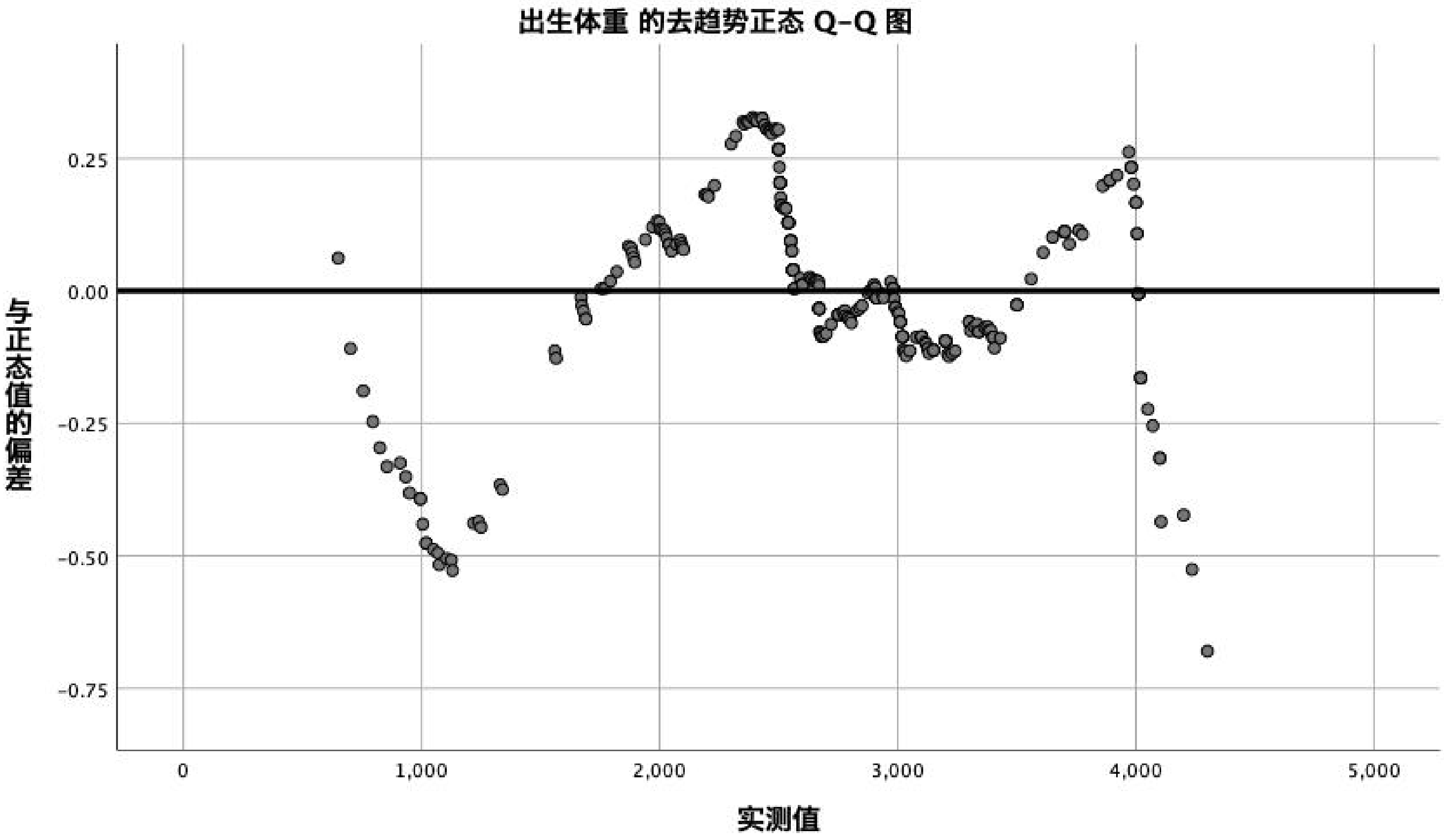

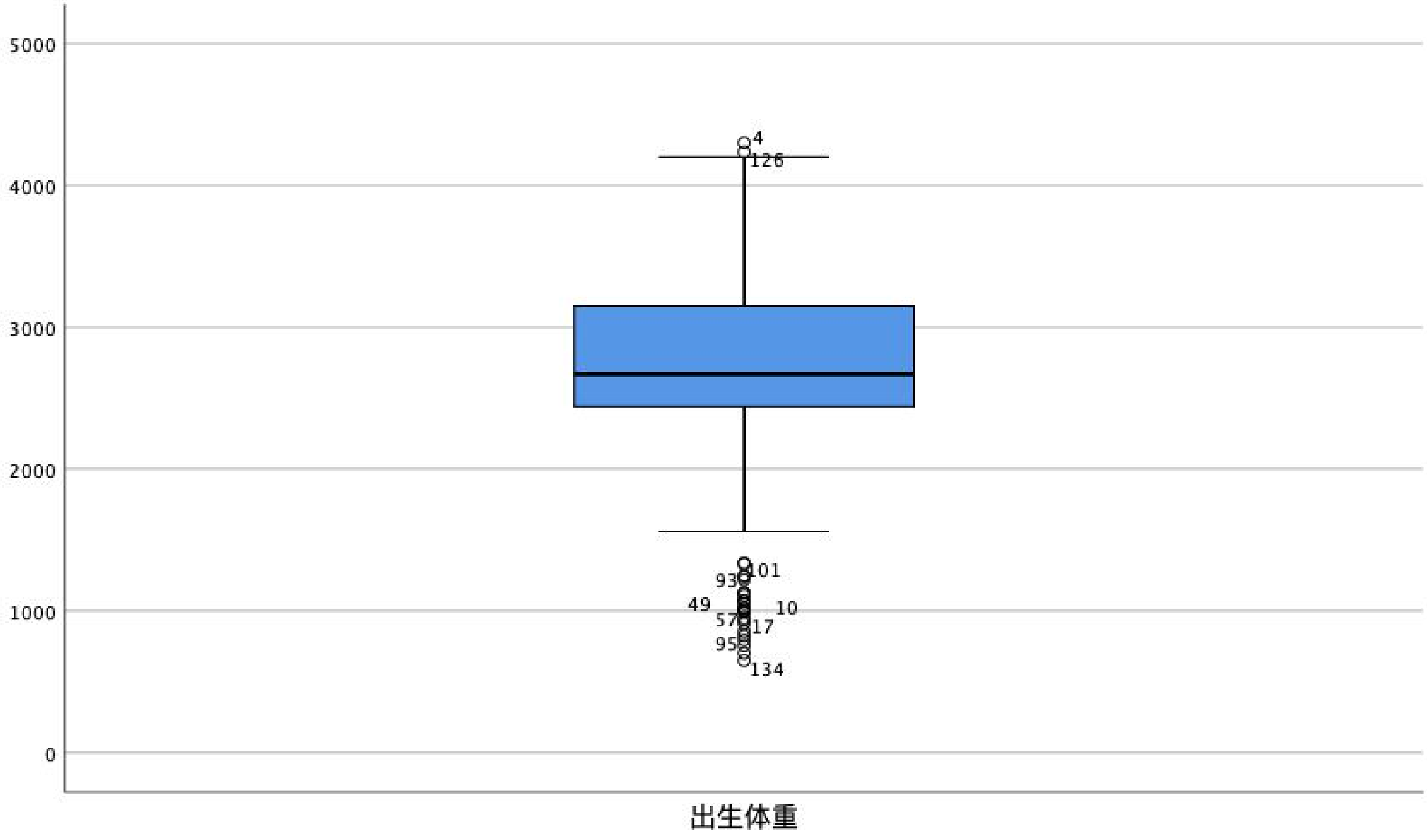


```
NPAR TESTS
  /M-W= 出生体重 BY 组别(1 2)
  /STATISTICS=DESCRIPTIVES QUARTILES
```

/MISSING ANALYSIS.

NPar 检验

备注

已创建输出		27-JUN-2022 11:50:23
注释		
输入	数据	/Users/jasmine/Documents/工作/我的科研课题/GDD早期家庭干预队列/GDD 数据集.sav
	活动数据集	数据集1
	过滤器	<无>
	权重	<无>
	拆分文件	<无>
	工作数据文件中的行数	306
缺失值处理	对缺失的定义	将用户定义的缺失值视为缺失。
	使用的个案数	每项检验的统计都基于所有对于该检验中使用的变量具有有效数据的个案。
语法		NPAR TESTS<br>/M-W= 出生体重 BY 组别(1 2)<br><br>/STATISTICS=DESCRIPTIVES QUARTILES<br>/MISSING ANALYSIS.
资源	处理程序时间	00:00:00.00
	耗用时间	00:00:00.00
	允许的个案数[a]	449389

a. 基于工作空间内存的可用性。

描述统计

	个案数	平均值	标准 偏差	最小值	最大值	百分位数 第 25 个	第 50 个（中位数）	第 75 个
出生体重	306	2715.11	776.686	650	4300	2437.50	2670.00	3150.00

组别	306	1.50	.501	1	2	1.00	1.50	2.00

曼-惠特尼检验

秩

	组别	个案数	秩平均值	秩的总和
出生体重	干预组	153	150.04	22955.50
	对照组	153	156.96	24015.50
	总计	306		

检验统计[a]

	出生体重
曼-惠特尼 U	11174.500
威尔科克森 W	22955.500
Z	-.685
渐近显著性（双尾）	.493

a. 分组变量：组别

```
NPAR TESTS
  /M-W= 出生体重 BY 组别(1 2)
  /STATISTICS=DESCRIPTIVES QUARTILES
  /MISSING ANALYSIS
  /METHOD=EXACT TIMER(5).
```

NPar 检验

备注

已创建输出		27-JUN-2022 11:52:21
注释		
输入	数据	/Users/jasmine/Documents/工作/我的科研课题/GDD早期家庭干预队列/GDD 数据集.sav

	活动数据集	数据集1
	过滤器	<无>
	权重	<无>
	拆分文件	<无>
	工作数据文件中的行数	306
缺失值处理	对缺失的定义	将用户定义的缺失值视为缺失。
	使用的个案数	每项检验的统计都基于所有对于该检验中使用的变量具有有效数据的个案。
语法		NPAR TESTS<br>/M-W= 出生体重 BY 组别(1 2)<br><br>/STATISTICS=DESCRIPTIVES QUARTILES<br>/MISSING ANALYSIS<br>/METHOD=EXACT TIMER(5).
资源	处理程序时间	00:00:05.49
	耗用时间	00:00:05.00
	允许的个案数[a]	449389
	精确统计的时间	0:00:05.44

a. 基于工作空间内存的可用性。

警告

内存不足，无法计算准确的统计。

描述统计

	个案数	平均值	标准 偏差	最小值	最大值	百分位数 第 25 个	第 50 个（中位数）	第 75 个
出生体重	306	2715.11	776.686	650	4300	2437.50	2670.00	3150.00
组别	306	1.50	.501	1	2	1.00	1.50	2.00

曼-惠特尼检验

秩

	组别	个案数	秩平均值	秩的总和
出生体重	干预组	153	150.04	22955.50
	对照组	153	156.96	24015.50
	总计	306		

检验统计[a,b]

	出生体重
曼-惠特尼 U	11174.500
威尔科克森 W	22955.500
Z	-.685
渐近显著性（双尾）	.493

a. 分组变量：组别
b. 由于内存不足，因此无法计算部分或全部精确显著性。

```
NPAR TESTS
  /M-W= 出生体重 BY 组别(1 2)
  /STATISTICS=DESCRIPTIVES QUARTILES
  /MISSING ANALYSIS
  /METHOD= MC CIN(99) SAMPLES(10000).
```

NPar 检验

备注

已创建输出		27-JUN-2022 11:52:58
注释		
输入	数据	/Users/jasmine/Documents/工作/我的科研课题/GDD早期家庭干预队列/GDD 数据集.sav
	活动数据集	数据集1
	过滤器	<无>
	权重	<无>
	拆分文件	<无>
	工作数据文件中的行数	306
缺失值处理	对缺失的定义	将用户定义的缺失值视为缺失。

	使用的个案数	每项检验的统计都基于所有对于该检验中使用的变量具有有效数据的个案。
语法		NPAR TESTS<br>/M-W= 出生体重 BY 组别(1 2)<br><br>/STATISTICS=DESCRIPTIVES QUARTILES<br>/MISSING ANALYSIS<br>/METHOD= MC CIN(99) SAMPLES(10000).
资源	处理程序时间	00:00:00.11
	耗用时间	00:00:00.00
	允许的个案数[a]	449389
	精确统计的时间	0:00:00.12

a. 基于工作空间内存的可用性。

描述统计

	个案数	平均值	标准 偏差	最小值	最大值	百分位数 第 25 个	第 50 个（中位数）	第 75 个
出生体重	306	2715.11	776.686	650	4300	2437.50	2670.00	3150.00
组别	306	1.50	.501	1	2	1.00	1.50	2.00

曼-惠特尼检验

秩

	组别	个案数	秩平均值	秩的总和
出生体重	干预组	153	150.04	22955.50
	对照组	153	156.96	24015.50
	总计	306		

检验统计[a]

	出生体重
曼-惠特尼 U	11174.500
威尔科克森 W	22955.500
Z	-.685

渐近显著性（双尾）			.493
蒙特卡洛显著性（双尾）	显著性		.489[b]
	99% 置信区间	下限	.476
		上限	.502
蒙特卡洛显著性（单尾）	显著性		.241[b]
	99% 置信区间	下限	.230
		上限	.252

a. 分组变量：组别
b. 基于 10000 个抽样表，起始种子为 2000000。

```
CROSSTABS
  /TABLES=组别 BY 性别
  /FORMAT=AVALUE TABLES
  /STATISTICS=CHISQ
  /CELLS=COUNT EXPECTED ROW COLUMN TOTAL
  /COUNT ROUND CELL
  /BARCHART.
```

交叉表

备注

已创建输出		27-JUN-2022 16:04:11
注释		
输入	数据	/Users/jasmine/Documents/工作/我的科研课题/GDD早期家庭干预队列/GDD 数据集.sav
	活动数据集	数据集1
	过滤器	<无>
	权重	<无>
	拆分文件	<无>
	工作数据文件中的行数	306
缺失值处理	对缺失的定义	将用户定义的缺失值视为缺失。
	使用的个案数	每个表的统计都基于每个表中所有符合以下条件的个案：对于所有变量，都具有指定范围内的有效数据。

语法		CROSSTABS<br>/TABLES=组别 BY 性别<br>/FORMAT=AVALUE TABLES<br>/STATISTICS=CHISQ<br>/CELLS=COUNT EXPECTED ROW COLUMN TOTAL<br>/COUNT ROUND CELL<br>/BARCHART.
资源	处理程序时间	00:00:00.16
	耗用时间	00:00:00.00
	请求的维	2
	可用单元格	524245

个案处理摘要

	个案					
	有效		缺失		总计	
	N	百分比	N	百分比	N	百分比
组别 * 性别	306	100.0%	0	0.0%	306	100.0%

组别 * 性别 交叉表

			性别		
			男性	女性	总计
组别	干预组	计数	86	67	153
		期望计数	90.5	62.5	153.0
		占 组别 的百分比	56.2%	43.8%	100.0%
		占 性别 的百分比	47.5%	53.6%	50.0%
		占总计的百分比	28.1%	21.9%	50.0%
	对照组	计数	95	58	153
		期望计数	90.5	62.5	153.0
		占 组别 的百分比	62.1%	37.9%	100.0%
		占 性别 的百分比	52.5%	46.4%	50.0%
		占总计的百分比	31.0%	19.0%	50.0%
总计		计数	181	125	306
		期望计数	181.0	125.0	306.0
		占 组别 的百分比	59.2%	40.8%	100.0%
		占 性别 的百分比	100.0%	100.0%	100.0%
		占总计的百分比	59.2%	40.8%	100.0%

卡方检验

	值	自由度	渐进显著性（双侧）	精确显著性（双侧）	精确显著性（单侧）
皮尔逊卡方	1.096[a]	1	.295		
连续性修正[b]	.866	1	.352		
似然比	1.096	1	.295		
费希尔精确检验				.352	.176
线性关联	1.092	1	.296		
有效个案数	306				

a. 0 个单元格 (.0%) 的期望计数小于 5。最小期望计数为 62.50。
b. 仅针对 2x2 表进行计算

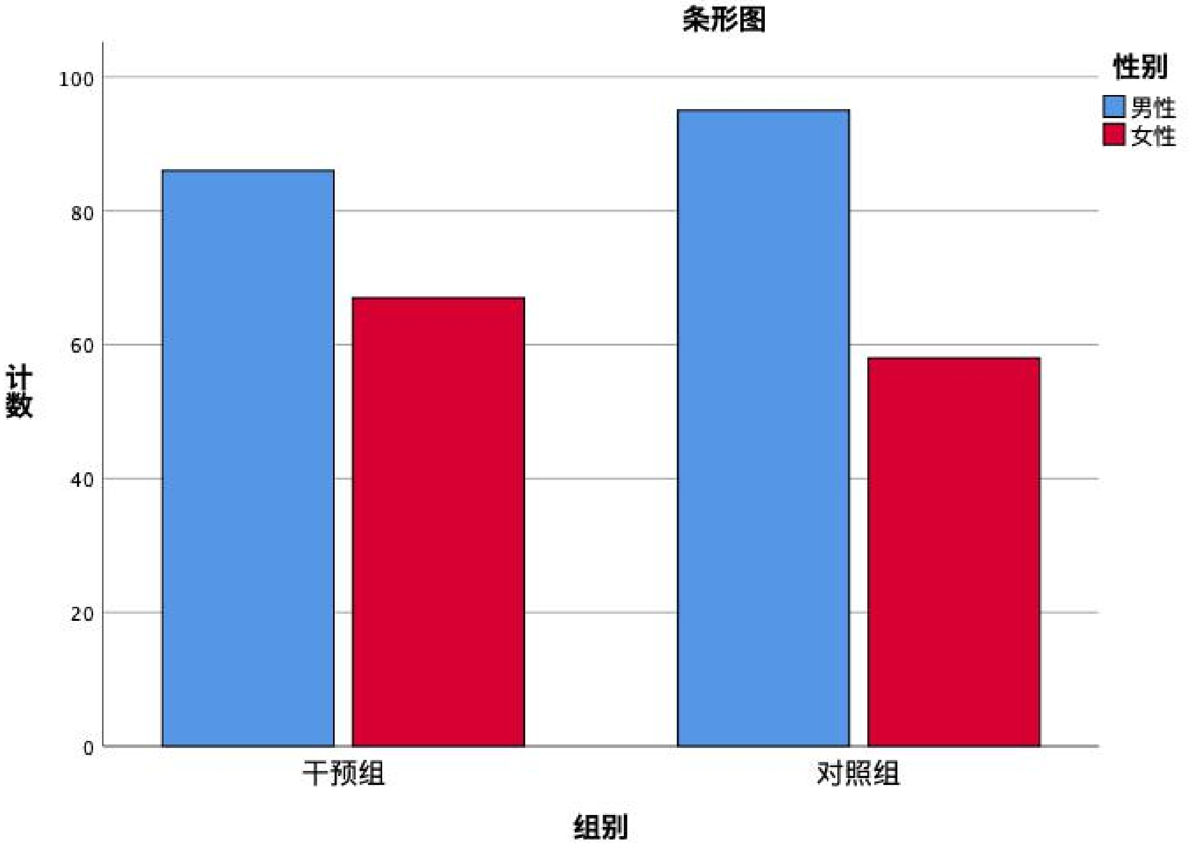


```
CROSSTABS
  /TABLES=组别 BY 分娩方式
  /FORMAT=AVALUE TABLES
  /STATISTICS=CHISQ
  /CELLS=COUNT EXPECTED ROW COLUMN TOTAL
  /COUNT ROUND CELL
  /BARCHART.
```

交叉表

备注

已创建输出		27-JUN-2022 16:27:14
注释		
输入	数据	/Users/jasmine/Documents/工作/我的科研课题/GDD早期家庭干预队列/GDD 数据集.sav
	活动数据集	数据集1
	过滤器	<无>
	权重	<无>
	拆分文件	<无>
	工作数据文件中的行数	306
缺失值处理	对缺失的定义	将用户定义的缺失值视为缺失。
	使用的个案数	每个表的统计都基于每个表中所有符合以下条件的个案：对于所有变量，都具有指定范围内的有效数据。
语法		CROSSTABS<br>/TABLES=组别 BY 分娩方式<br>/FORMAT=AVALUE TABLES<br>/STATISTICS=CHISQ<br>/CELLS=COUNT EXPECTED ROW COLUMN TOTAL<br>/COUNT ROUND CELL<br>/BARCHART.
资源	处理程序时间	00:00:00.14
	耗用时间	00:00:00.00
	请求的维	2
	可用单元格	524245

个案处理摘要

	个案					
	有效		缺失		总计	
	N	百分比	N	百分比	N	百分比
组别 * 分娩方式	306	100.0%	0	0.0%	306	100.0%

组别 * 分娩方式 交叉表

			分娩方式		
			顺产	剖腹产	总计
组别	干预组	计数	52	101	153
		期望计数	55.0	98.0	153.0
		占 组别 的百分比	34.0%	66.0%	100.0%
		占 分娩方式 的百分比	47.3%	51.5%	50.0%
		占总计的百分比	17.0%	33.0%	50.0%
	对照组	计数	58	95	153
		期望计数	55.0	98.0	153.0
		占 组别 的百分比	37.9%	62.1%	100.0%
		占 分娩方式 的百分比	52.7%	48.5%	50.0%
		占总计的百分比	19.0%	31.0%	50.0%
总计		计数	110	196	306
		期望计数	110.0	196.0	306.0
		占 组别 的百分比	35.9%	64.1%	100.0%
		占 分娩方式 的百分比	100.0%	100.0%	100.0%
		占总计的百分比	35.9%	64.1%	100.0%

卡方检验

	值	自由度	渐进显著性（双侧）	精确显著性（双侧）	精确显著性（单侧）
皮尔逊卡方	.511[a]	1	.475		
连续性修正[b]	.355	1	.551		
似然比	.511	1	.475		
费希尔精确检验				.551	.276
线性关联	.509	1	.475		
有效个案数	306				

a. 0 个单元格 (.0%) 的期望计数小于 5。最小期望计数为 55.00。
b. 仅针对 2x2 表进行计算

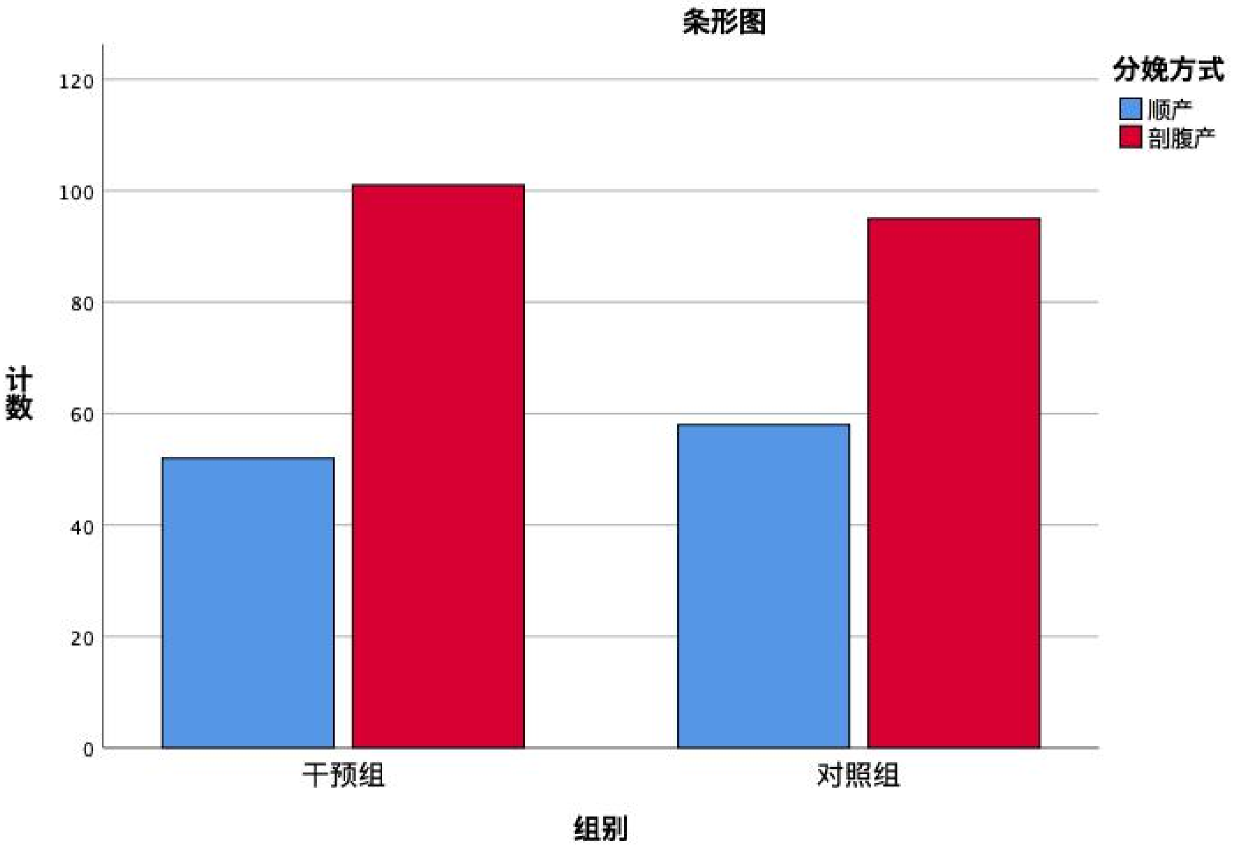


```
CROSSTABS
  /TABLES=组别 BY 父母受教育程度
  /FORMAT=AVALUE TABLES
  /STATISTICS=CHISQ
  /CELLS=COUNT EXPECTED ROW COLUMN TOTAL
  /COUNT ROUND CELL
  /BARCHART.
```

交叉表

备注

已创建输出		27-JUN-2022 16:37:37
注释		
输入	数据	/Users/jasmine/Documents/工作/我的科研课题/GDD早期家庭干预队列/GDD 数据集.sav
	活动数据集	数据集1
	过滤器	<无>

	权重	<无>
	拆分文件	<无>
	工作数据文件中的行数	306
缺失值处理	对缺失的定义	将用户定义的缺失值视为缺失。
	使用的个案数	每个表的统计都基于每个表中所有符合以下条件的个案：对于所有变量，都具有指定范围内的有效数据。
语法		CROSSTABS<br>/TABLES=组别 BY 父母受教育程度<br>/FORMAT=AVALUE TABLES<br>/STATISTICS=CHISQ<br>/CELLS=COUNT EXPECTED ROW COLUMN TOTAL<br>/COUNT ROUND CELL<br>/BARCHART.
资源	处理程序时间	00:00:00.13
	耗用时间	00:00:00.00
	请求的维	2
	可用单元格	524245

个案处理摘要

	个案					
	有效		缺失		总计	
	N	百分比	N	百分比	N	百分比
组别 * 父母受教育程度	306	100.0%	0	0.0%	306	100.0%

组别 * 父母受教育程度 交叉表

			父母受教育程度		
			<12 yr of education	>12 yr of education	总计
组别	干预组	计数	76	77	153
		期望计数	77.0	76.0	153.0
		占 组别 的百分比	49.7%	50.3%	100.0%
		占 父母受教育程度 的百分比	49.4%	50.7%	50.0%
		占总计的百分比	24.8%	25.2%	50.0%
	对照组	计数	78	75	153
		期望计数	77.0	76.0	153.0
		占 组别 的百分比	51.0%	49.0%	100.0%

	占 父母受教育程度 的百分比	50.6%	49.3%	50.0%
	占总计的百分比	25.5%	24.5%	50.0%
总计	计数	154	152	306
	期望计数	154.0	152.0	306.0
	占 组别 的百分比	50.3%	49.7%	100.0%
	占 父母受教育程度 的百分比	100.0%	100.0%	100.0%
	占总计的百分比	50.3%	49.7%	100.0%

卡方检验

	值	自由度	渐进显著性（双侧）	精确显著性（双侧）	精确显著性（单侧）
皮尔逊卡方	.052[a]	1	.819		
连续性修正[b]	.013	1	.909		
似然比	.052	1	.819		
费希尔精确检验				.909	.454
线性关联	.052	1	.819		
有效个案数	306				

a. 0 个单元格 (.0%) 的期望计数小于 5。最小期望计数为 76.00。
b. 仅针对 2x2 表进行计算

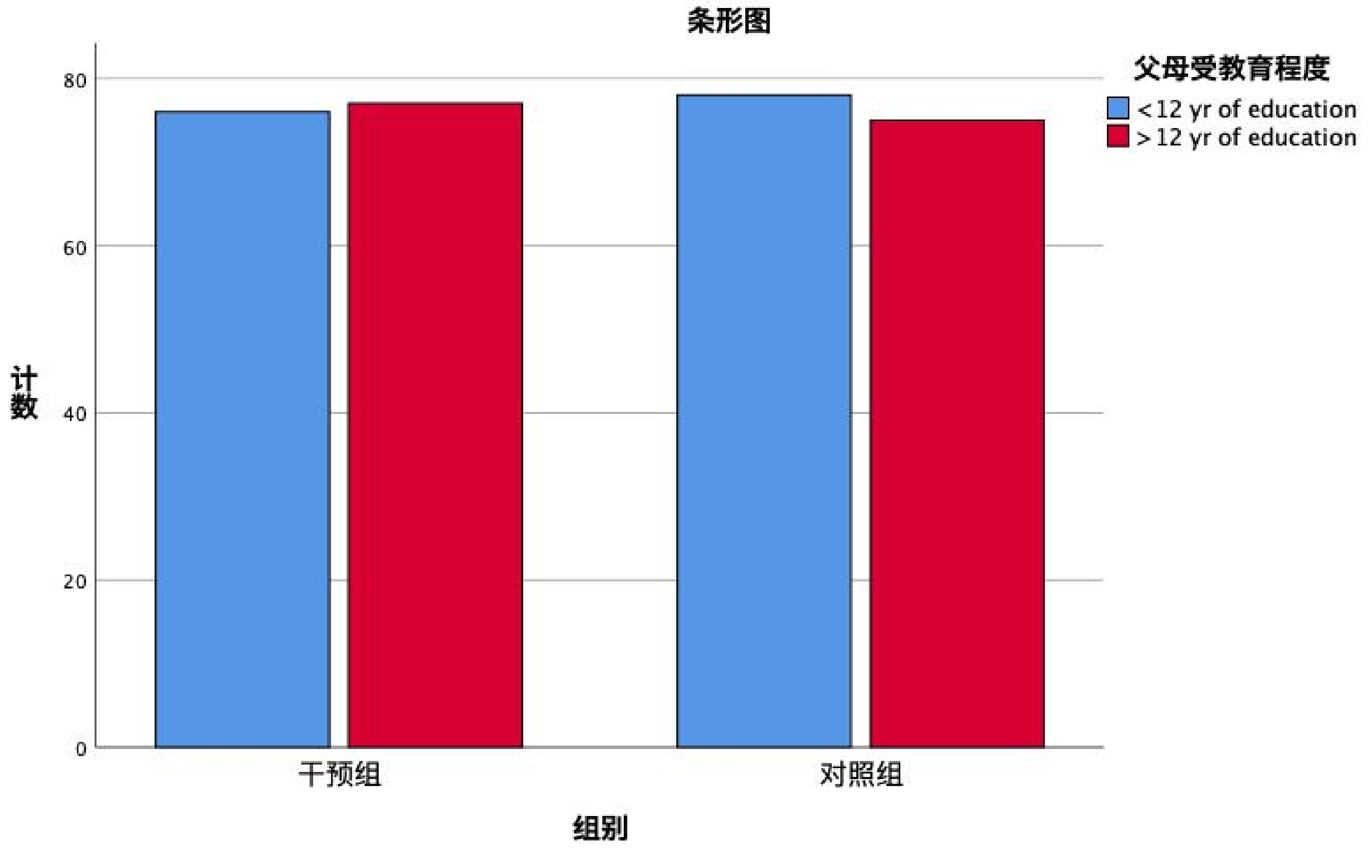

```
CROSSTABS
  /TABLES=组别 BY 家庭年收入
  /FORMAT=AVALUE TABLES
  /STATISTICS=CHISQ
  /CELLS=COUNT EXPECTED ROW COLUMN TOTAL
  /COUNT ROUND CELL
  /BARCHART.
```

交叉表

备注

已创建输出		27-JUN-2022 16:48:48
注释		
输入	数据	/Users/jasmine/Documents/工作/我的科研课题/GDD早期家庭干预队列/GDD 数据集.sav
	活动数据集	数据集1
	过滤器	<无>
	权重	<无>
	拆分文件	<无>
	工作数据文件中的行数	306
缺失值处理	对缺失的定义	将用户定义的缺失值视为缺失。
	使用的个案数	每个表的统计都基于每个表中所有符合以下条件的个案：对于所有变量，都具有指定范围内的有效数据。
语法		CROSSTABS<br>/TABLES=组别 BY 家庭年收入<br>/FORMAT=AVALUE TABLES<br>/STATISTICS=CHISQ<br>/CELLS=COUNT EXPECTED ROW COLUMN TOTAL<br>/COUNT ROUND CELL<br>/BARCHART.
资源	处理程序时间	00:00:00.15
	耗用时间	00:00:00.00

请求的维	2
可用单元格	524245

个案处理摘要

	个案					
	有效		缺失		总计	
	N	百分比	N	百分比	N	百分比
组别 * 家庭年收入	306	100.0%	0	0.0%	306	100.0%

组别 * 家庭年收入 交叉表

			家庭年收入		
			<20,000 USD	>20,000 USD	总计
组别	干预组	计数	76	77	153
		期望计数	73.5	79.5	153.0
		占 组别 的百分比	49.7%	50.3%	100.0%
		占 家庭年收入 的百分比	51.7%	48.4%	50.0%
		占总计的百分比	24.8%	25.2%	50.0%
	对照组	计数	71	82	153
		期望计数	73.5	79.5	153.0
		占 组别 的百分比	46.4%	53.6%	100.0%
		占 家庭年收入 的百分比	48.3%	51.6%	50.0%
		占总计的百分比	23.2%	26.8%	50.0%
总计		计数	147	159	306
		期望计数	147.0	159.0	306.0
		占 组别 的百分比	48.0%	52.0%	100.0%
		占 家庭年收入 的百分比	100.0%	100.0%	100.0%
		占总计的百分比	48.0%	52.0%	100.0%

卡方检验

	值	自由度	渐进显著性（双侧）	精确显著性（双侧）	精确显著性（单侧）
皮尔逊卡方	.327[a]	1	.567		
连续性修正[b]	.209	1	.647		
似然比	.327	1	.567		
费希尔精确检验				.647	.324
线性关联	.326	1	.568		
有效个案数	306				

a. 0 个单元格（.0%）的期望计数小于 5。最小期望计数为 73.50。

b. 仅针对 2x2 表进行计算

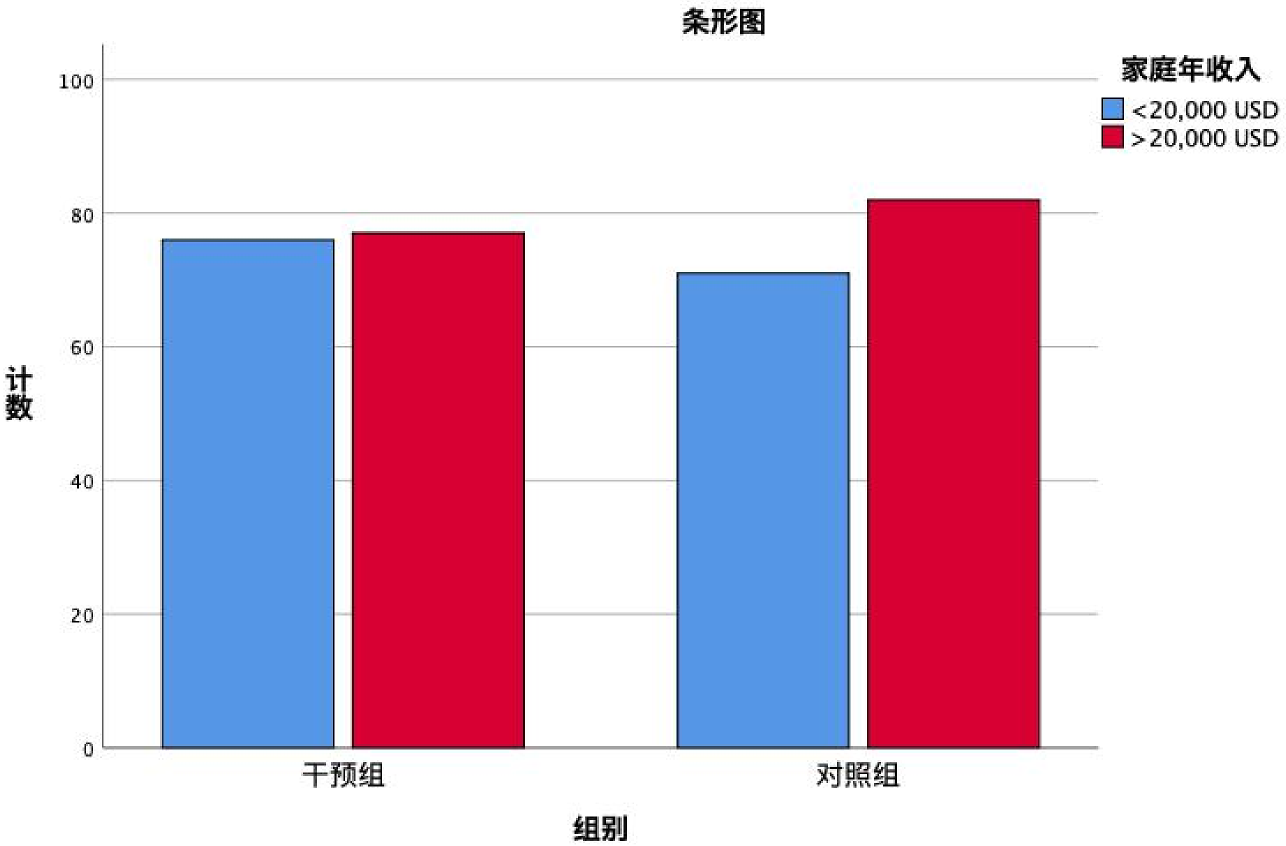


```
DATASET ACTIVATE 数据集1.

SAVE OUTFILE='/Users/jasmine/Documents/工作/我的科研课题/GDD早期家庭干预队列/GDD
数据集.sav'
  /COMPRESSED.
DATASET ACTIVATE 数据集1.

SAVE OUTFILE='/Users/jasmine/Documents/工作/我的科研课题/GDD早期家庭干预队列/GDD
数据集.sav'
  /COMPRESSED.
CROSSTABS
  /TABLES=组别 BY 父母平均年龄
  /FORMAT=AVALUE TABLES
  /STATISTICS=CHISQ
  /CELLS=COUNT EXPECTED ROW COLUMN TOTAL
  /COUNT ROUND CELL
  /BARCHART.
```

交叉表

备注

已创建输出		27-JUN-2022 17:03:43
注释		
输入	数据	/Users/jasmine/Documents/工作/我的科研课题/GDD早期家庭干预队列/GDD 数据集.sav
	活动数据集	数据集1
	过滤器	<无>
	权重	<无>
	拆分文件	<无>
	工作数据文件中的行数	306
缺失值处理	对缺失的定义	将用户定义的缺失值视为缺失。
	使用的个案数	每个表的统计都基于每个表中所有符合以下条件的个案：对于所有变量，都具有指定范围内的有效数据。
语法		CROSSTABS<br>/TABLES=组别 BY 父母平均年龄<br>/FORMAT=AVALUE TABLES<br>/STATISTICS=CHISQ<br>/CELLS=COUNT EXPECTED ROW COLUMN TOTAL<br>/COUNT ROUND CELL<br>/BARCHART.
资源	处理程序时间	00:00:00.19
	耗用时间	00:00:01.00
	请求的维	2
	可用单元格	524245

个案处理摘要

	个案					
	有效		缺失		总计	
	N	百分比	N	百分比	N	百分比
组别 * 父母平均年龄	306	100.0%	0	0.0%	306	100.0%

组别 * 父母平均年龄 交叉表

	父母平均年龄	总计

			30 and under	31 and over	
组别	干预组	计数	98	55	153
		期望计数	94.5	58.5	153.0
		占 组别 的百分比	64.1%	35.9%	100.0%
		占 父母平均年龄 的百分比	51.9%	47.0%	50.0%
		占总计的百分比	32.0%	18.0%	50.0%
	对照组	计数	91	62	153
		期望计数	94.5	58.5	153.0
		占 组别 的百分比	59.5%	40.5%	100.0%
		占 父母平均年龄 的百分比	48.1%	53.0%	50.0%
		占总计的百分比	29.7%	20.3%	50.0%
总计		计数	189	117	306
		期望计数	189.0	117.0	306.0
		占 组别 的百分比	61.8%	38.2%	100.0%
		占 父母平均年龄 的百分比	100.0%	100.0%	100.0%
		占总计的百分比	61.8%	38.2%	100.0%

卡方检验

	值	自由度	渐进显著性（双侧）	精确显著性（双侧）	精确显著性（单侧）
皮尔逊卡方	.678[a]	1	.410		
连续性修正[b]	.498	1	.480		
似然比	.678	1	.410		
费希尔精确检验				.480	.240
线性关联	.676	1	.411		
有效个案数	306				

a. 0 个单元格 (.0%) 的期望计数小于 5。最小期望计数为 58.50。
b. 仅针对 2x2 表进行计算

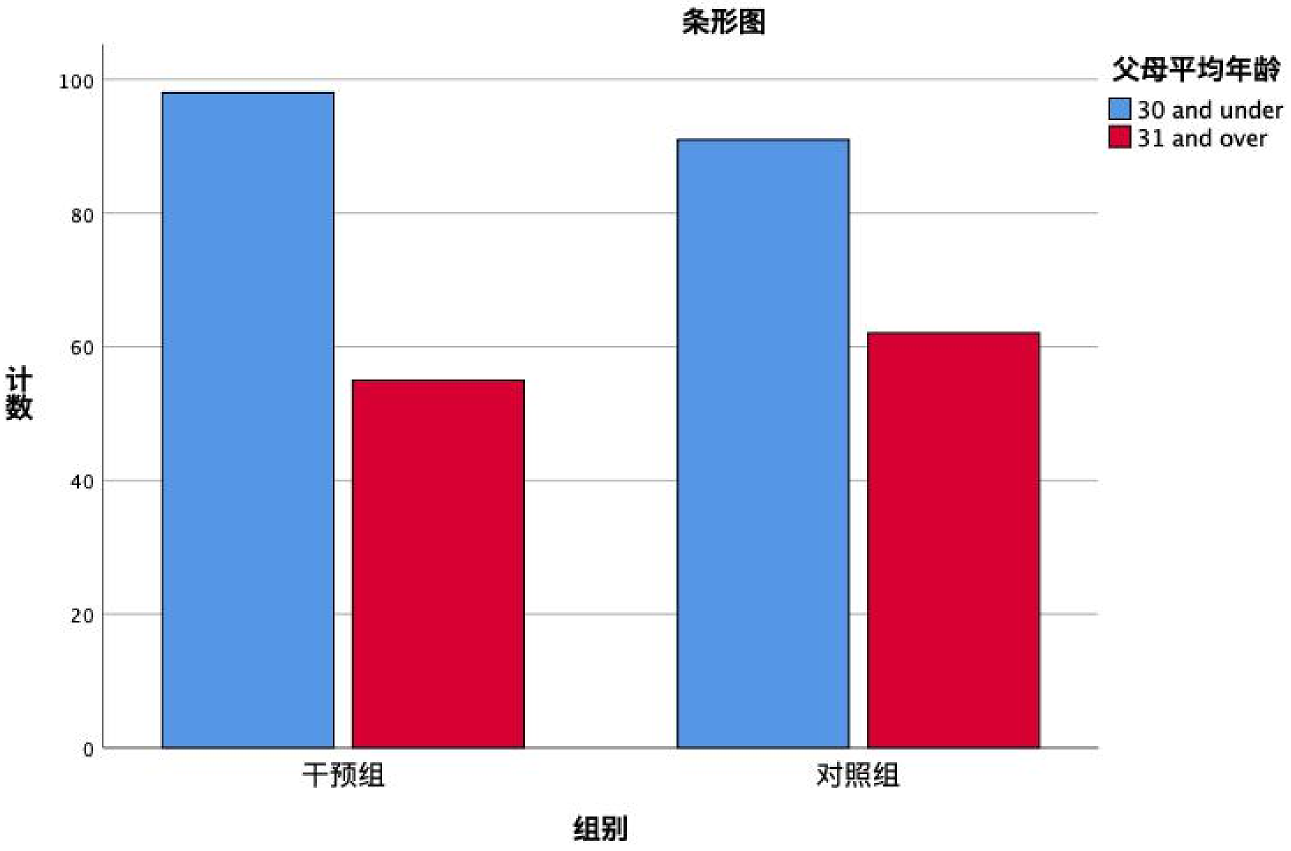

GET
FILE='/Users/jasmine/Documents/工作/我的科研课题/GDD早期家庭干预队列/GDD 总数据集.sav'.
DATASET NAME 数据集1 WINDOW=FRONT.

GET
FILE='/Users/jasmine/Documents/工作/我的科研课题/GDD早期家庭干预队列/GDD 数据集.sav'.
DATASET NAME 数据集1 WINDOW=FRONT.
EXAMINE VARIABLES=DA_A_T1 BY 组别
/PLOT BOXPLOT STEMLEAF HISTOGRAM NPPLOT SPREADLEVEL(1)
/COMPARE GROUPS
/MESTIMATORS HUBER(1.339) ANDREW(1.34) HAMPEL(1.7,3.4,8.5) TUKEY(4.685)
/PERCENTILES(5,10,25,50,75,90,95) HAVERAGE
/STATISTICS DESCRIPTIVES EXTREME
/CINTERVAL 95
/MISSING LISTWISE
/NOTOTAL.

探索

备注

已创建输出		27-JUN-2022 20:23:11
注释		
输入	数据	/Users/jasmine/Documents/工作/我的科研课题/GDD早期家庭干预队列/GDD 数据集.sav
	活动数据集	数据集1
	过滤器	<无>
	权重	<无>
	拆分文件	<无>
	工作数据文件中的行数	306
缺失值处理	对缺失的定义	将因变量的用户定义缺失值视为缺失。
	使用的个案数	统计基于那些对任何所用因变量或因子都没有缺失值的个案。
语法		EXAMINE VARIABLES=DA_A_T1 BY 组别<br>/PLOT BOXPLOT STEMLEAF HISTOGRAM NPPLOT SPREADLEVEL(1)<br>/COMPARE GROUPS<br>/MESTIMATORS HUBER(1.339) ANDREW(1.34) HAMPEL(1.7,3.4,8.5) TUKEY(4.685)<br>/PERCENTILES(5,10,25,50,75,90,95) HAVERAGE<br>/STATISTICS DESCRIPTIVES EXTREME<br>/CINTERVAL 95<br>/MISSING LISTWISE<br>/NOTOTAL.
资源	处理程序时间	00:00:02.50
	耗用时间	00:00:02.00

[数据集1] /Users/jasmine/Documents/工作/我的科研课题/GDD早期家庭干预队列/GDD 数据集.sav

警告

由于没有足够的唯一分布/水平对，因此无法为 基线大运动发育龄*组别 计算分布-水平图的回归斜率。

组别

个案处理摘要

	组别	有效 N	有效 百分比	缺失 N	缺失 百分比	总计 N	总计 百分比
基线大运动发育龄	干预组	153	100.0%	0	0.0%	153	100.0%
	对照组	153	100.0%	0	0.0%	153	100.0%

描述

	组别			统计	标准误差
基线大运动发育龄	干预组	平均值		2.0425	.08435
		平均值的 95% 置信区间	下限	1.8758	
			上限	2.2091	
		5% 剪除后平均值		1.9880	
		中位数		2.0000	
		方差		1.089	
		标准偏差		1.04338	
		最小值		.50	
		最大值		5.00	
		范围		4.50	
		四分位距		2.00	
		偏度		.643	.196
		峰度		-.505	.390
	对照组	平均值		2.1340	.08436
		平均值的 95% 置信区间	下限	1.9673	
			上限	2.3007	
		5% 剪除后平均值		2.0606	
		中位数		2.0000	
		方差		1.089	
		标准偏差		1.04347	
		最小值		.50	
		最大值		5.00	
		范围		4.50	

	四分位距	2.00	
	偏度	.742	.196
	峰度	.044	.390

M 估计量

	组别	休伯 M 估计量[a]	图基双权[b]	汉佩尔 M 估计量[c]	安德鲁波[d]
基线大运动发育龄	干预组	1.9655	1.9687	2.0040	1.9688
	对照组	2.0509	2.0503	2.0841	2.0506

a. 加权常量为 1.339。
b. 加权常量为 4.685。
c. 加权常量为 1.700、3.400 和 8.500
d. 加权常量为 1.340*pi。

百分位数

		组别	百分位数 5	10	25	50	75	90	
加权平均（定义 1）	基线大运动发育龄	干预组	1.0000	1.0000	1.0000	2.0000	3.0000	3.5000	
		对照组	1.0000	1.0000	1.0000	2.0000	3.0000	3.5000	
图基枢纽	基线大运动发育龄	干预组			1.0000	2.0000	3.0000		
		对照组			1.0000	2.0000	3.0000		

极值

	组别			个案号	值
基线大运动发育龄	干预组	最大值	1	22	5.00
			2	40	5.00
			3	35	4.00
			4	37	4.00
			5	45	4.00[a]
		最小值	1	91	.50
			2	85	.50
			3	72	.50
			4	151	1.00
			5	150	1.00[b]
	对照组	最大值	1	177	5.00
			2	206	5.00
			3	209	5.00

		4	250	5.00
		5	297	5.00
	最小值	1	218	.50
		2	306	1.00
		3	304	1.00
		4	294	1.00
		5	293	1.00[b]

a. 在较大极值的表中，仅显示了不完整的个案列表（这些个案的值为 4.00）。

b. 在较小极值的表中，仅显示了不完整的个案列表（这些个案的值为 1.00）。

正态性检验

		柯尔莫戈洛夫-斯米诺夫(V)[a]			夏皮洛-威尔克		
	组别	统计	自由度	显著性	统计	自由度	显著性
基线大运动发育龄	干预组	.181	153	.000	.890	153	.000
	对照组	.159	153	.000	.896	153	.000

a. 里利氏显著性修正

方差齐性检验

		莱文统计	自由度 1	自由度 2	显著性
基线大运动发育龄	基于平均值	.003	1	304	.955
	基于中位数	.077	1	304	.782
	基于中位数并具有调整后自由度	.077	1	302.724	.782
	基于剪除后平均值	.012	1	304	.911

基线大运动发育龄

直方图(O)

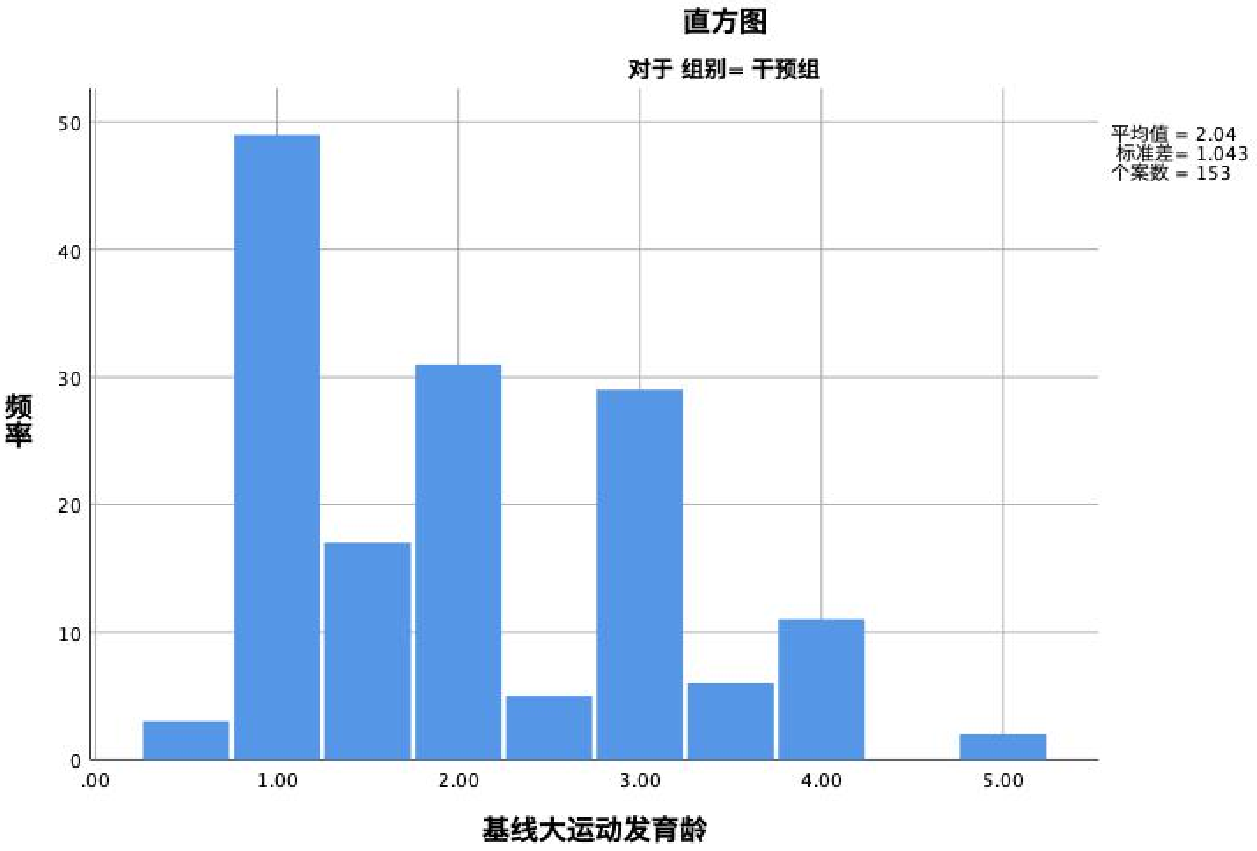
直方图
对于 组别= 干预组
平均值 = 2.04
标准差= 1.043
个案数 = 153
频率
基线大运动发育龄
.00
1.00
2.00
3.00
4.00
5.00
0
10
20
30
40
50

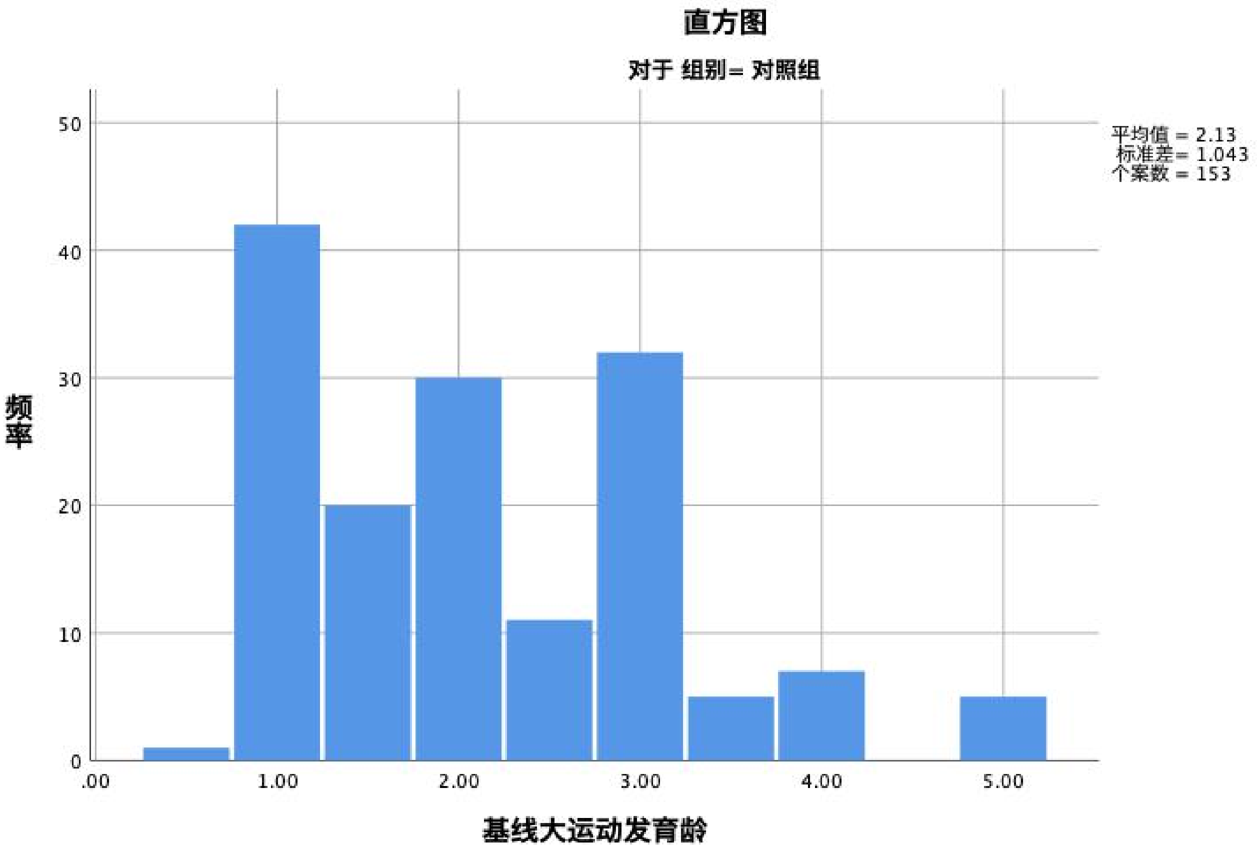
直方图
对于 组别= 对照组
平均值 = 2.13
标准差= 1.043
个案数 = 153
频率
基线大运动发育龄
.00
1.00
2.00
3.00
4.00
5.00
0
10
20
30
40
50

茎叶图

基线大运动发育龄 茎叶图：
组别= 干预组

频率	Stem &	叶
.00	0 .	
3.00	0 .	555
49.00	1 .	0000000000000000000000000000000000000000000000000
17.00	1 .	55555555555555555
31.00	2 .	0000000000000000000000000000000
5.00	2 .	55555
29.00	3 .	00000000000000000000000000000
6.00	3 .	555555
11.00	4 .	00000000000
.00	4 .	
2.00	5 .	00

主干宽度： 1.00
每个叶： 1 个案

基线大运动发育龄 茎叶图：
组别= 对照组

频率	Stem &	叶
.00	0 .	
1.00	0 .	5
42.00	1 .	000000000000000000000000000000000000000000
20.00	1 .	55555555555555555555
30.00	2 .	000000000000000000000000000000
11.00	2 .	55555555555
32.00	3 .	00000000000000000000000000000000
5.00	3 .	55555
7.00	4 .	0000000
.00	4 .	
5.00	5 .	00000

主干宽度： 1.00
每个叶： 1 个案

正态 Q-Q 图

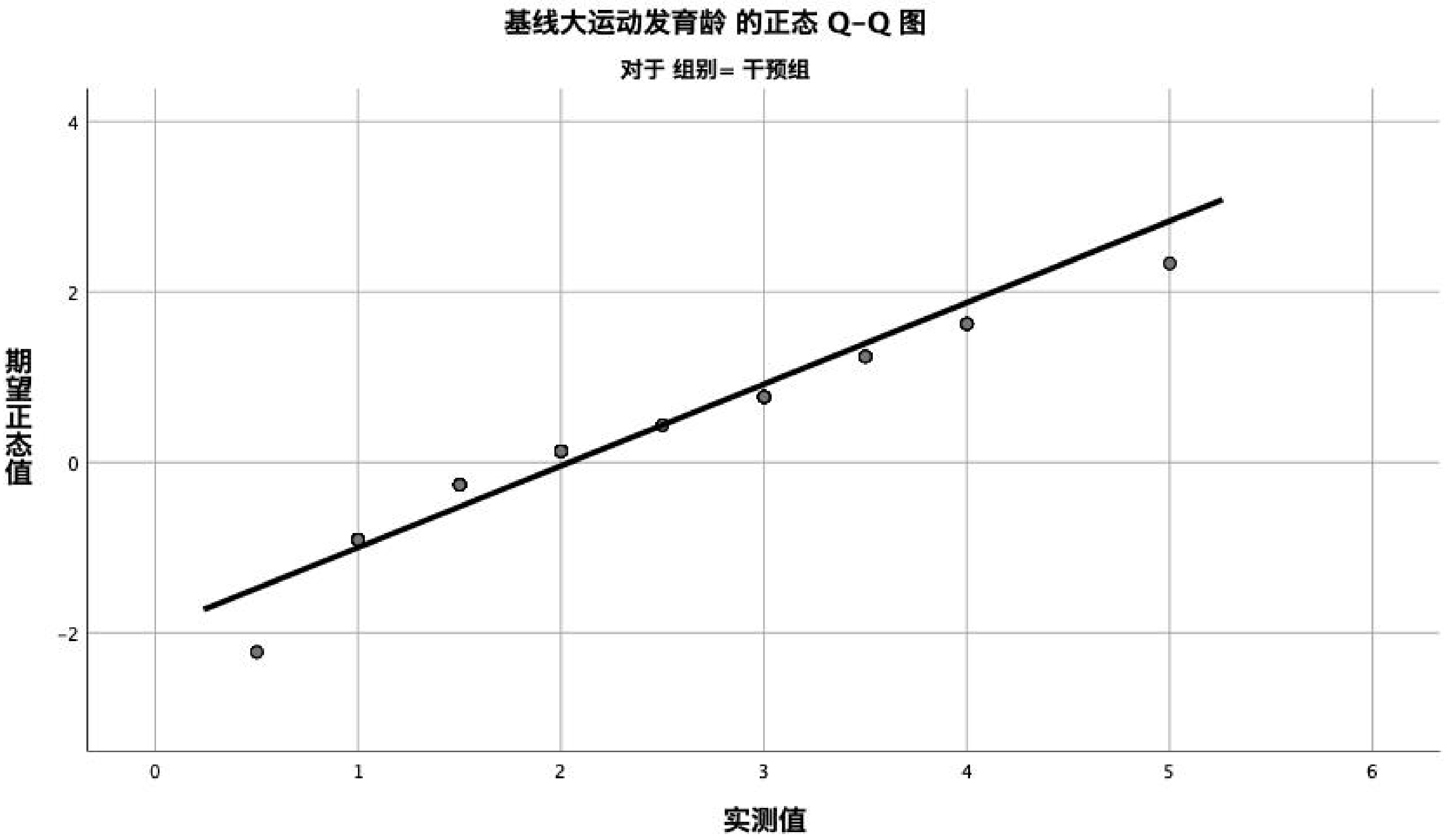

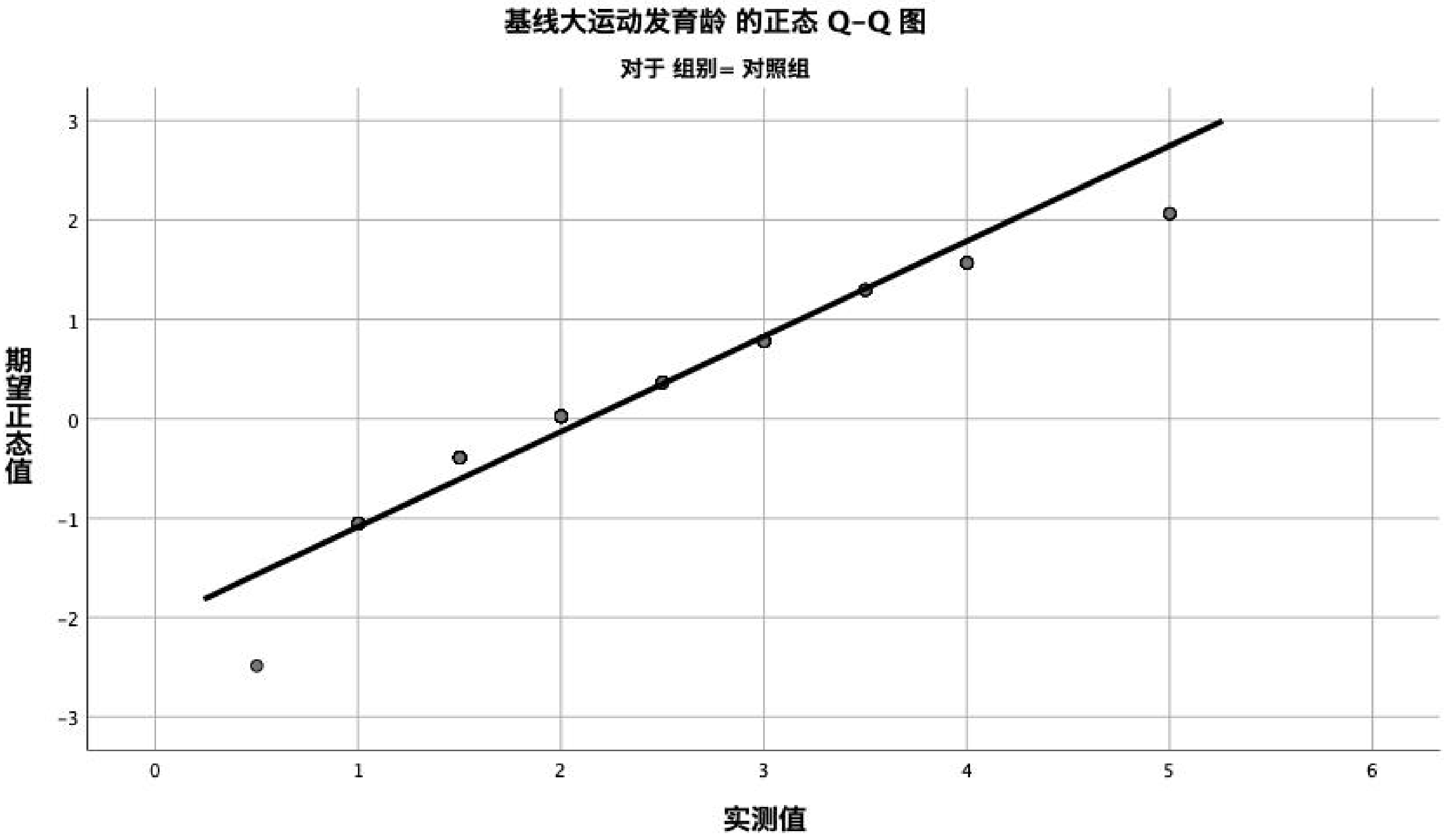

去趋势正态 Q-Q 图

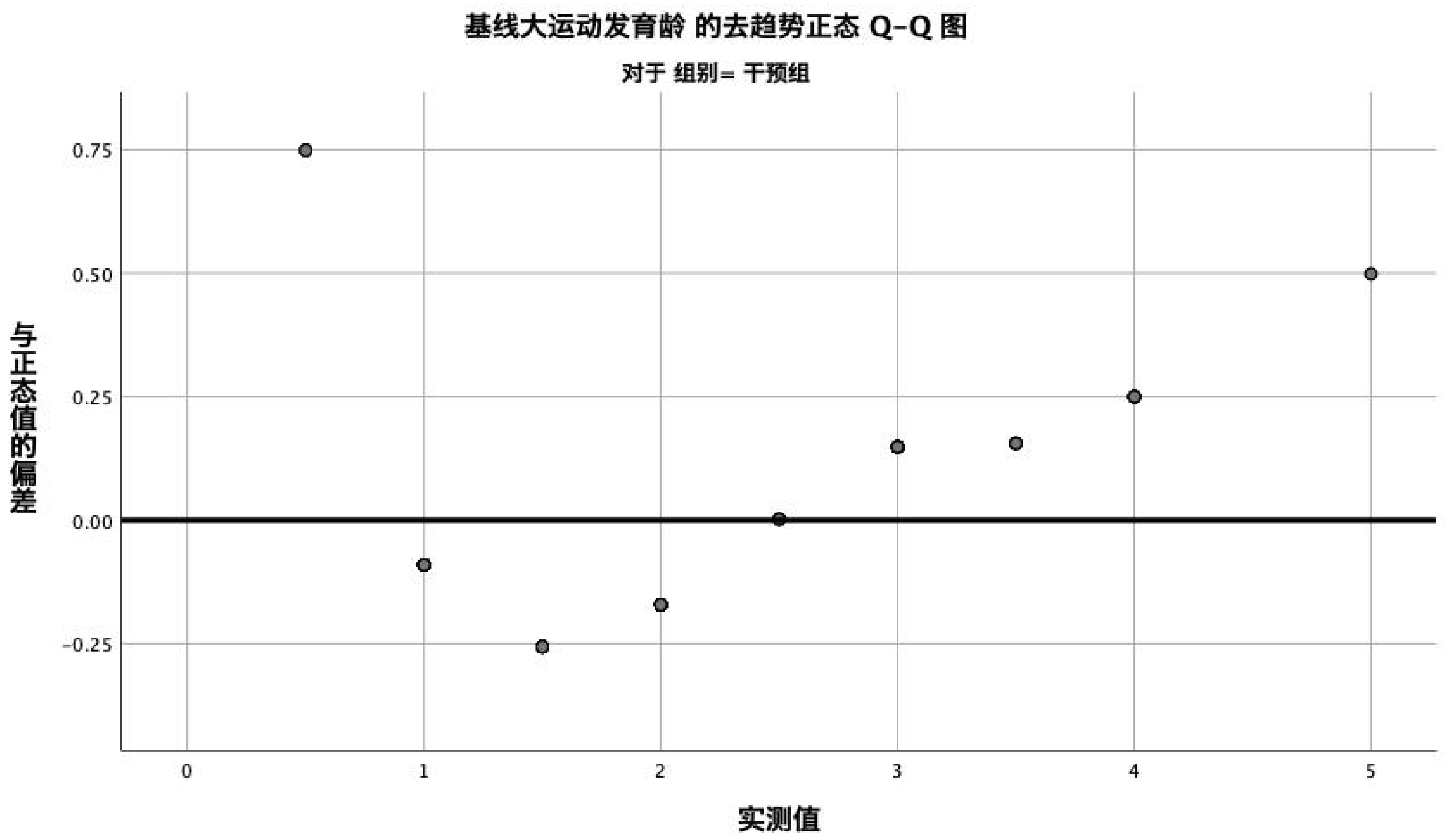

基线大运动发育龄 的去趋势正态 Q-Q 图
对于 组别= 干预组
与正态值的偏差
0.75
0.50
0.25
0.00
-0.25
0
1
2
3
4
5
实测值

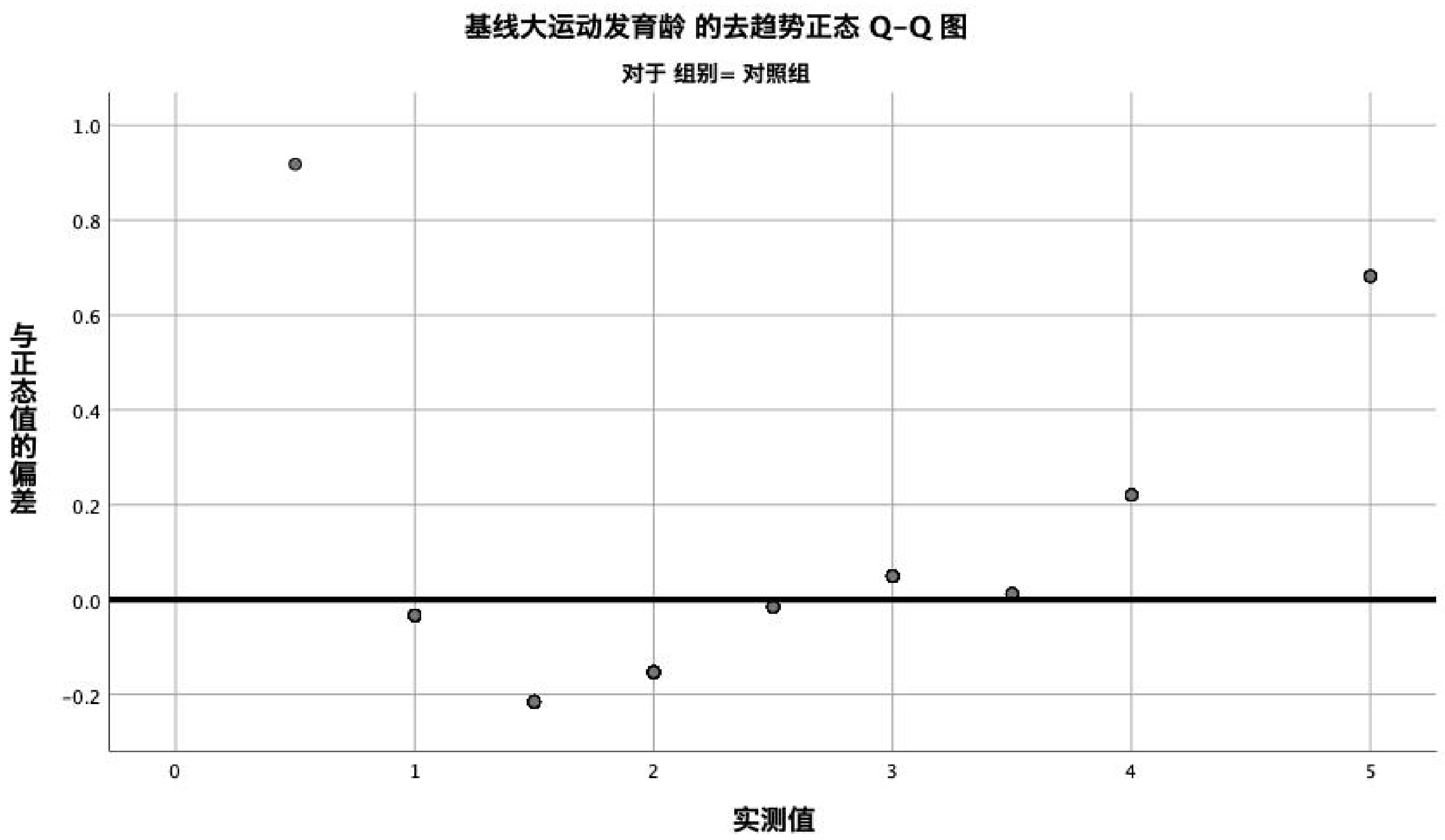

基线大运动发育龄 的去趋势正态 Q-Q 图
对于 组别= 对照组
与正态值的偏差
1.0
0.8
0.6
0.4
0.2
0.0
-0.2
0
1
2
3
4
5
实测值

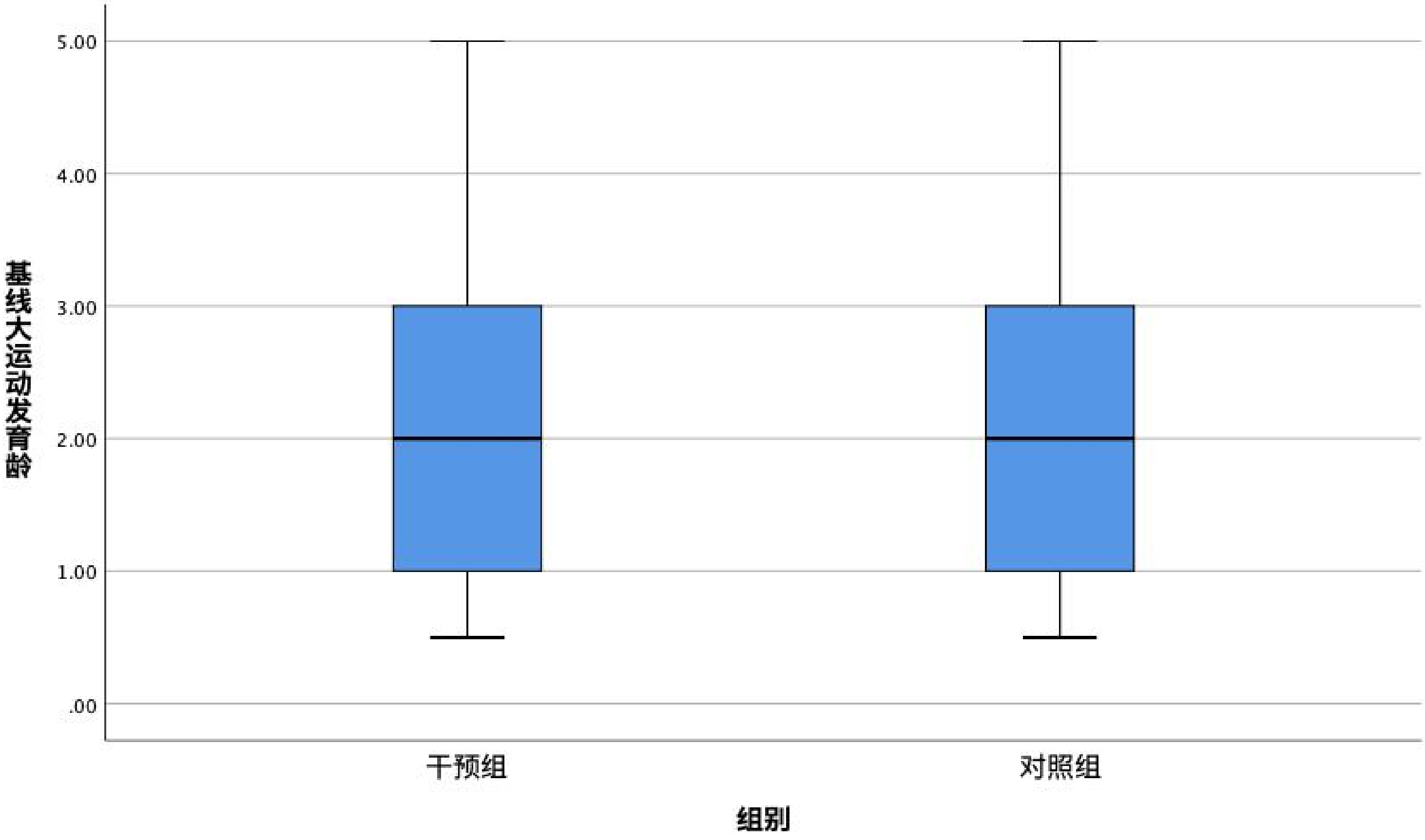

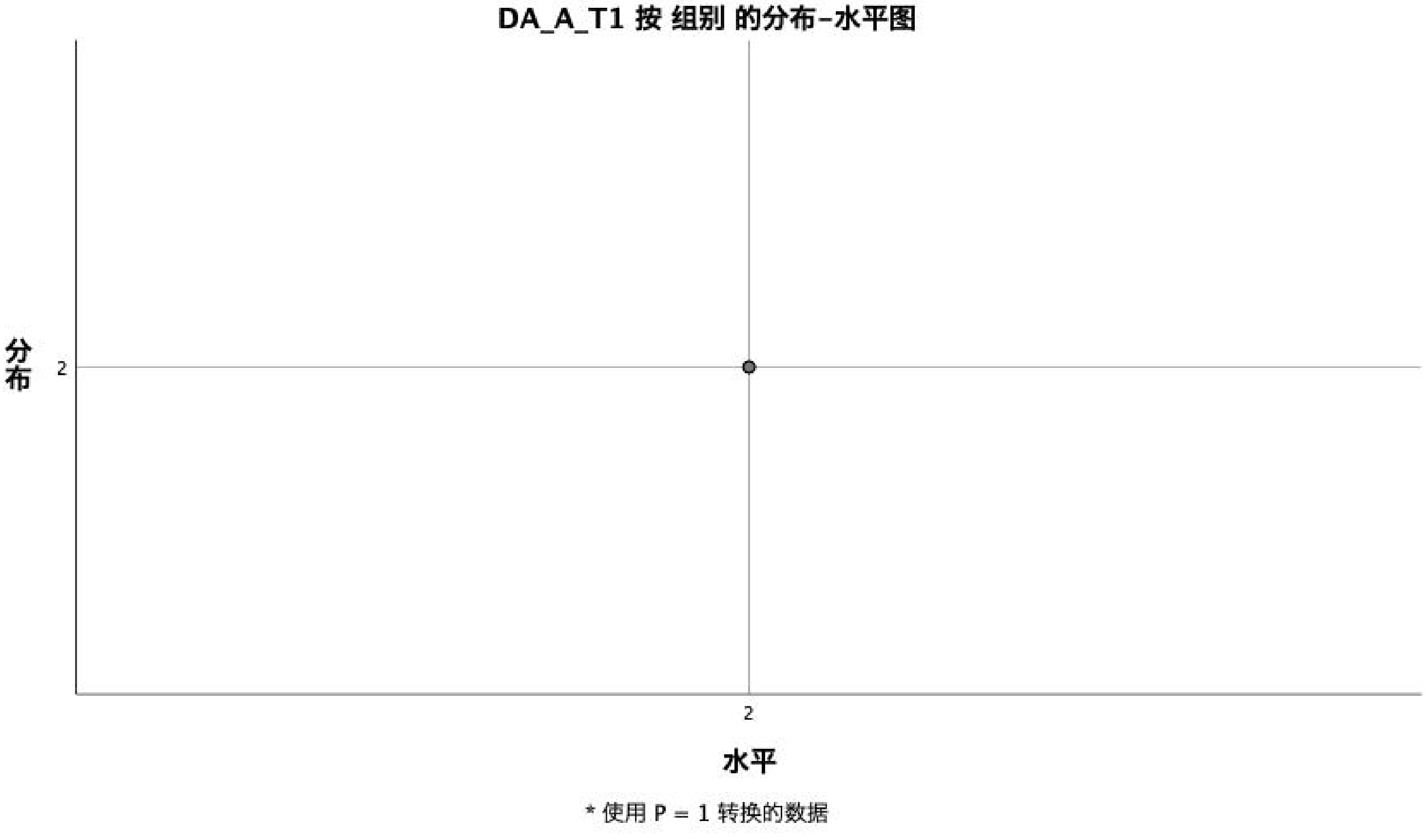


```
EXAMINE VARIABLES=DA_A_T1 BY 组别
  /PLOT BOXPLOT STEMLEAF HISTOGRAM NPPLOT SPREADLEVEL(1)
  /COMPARE GROUPS
```

```
/MESTIMATORS HUBER(1.339) ANDREW(1.34) HAMPEL(1.7,3.4,8.5) TUKEY(4.685)
/PERCENTILES(5,10,25,50,75,90,95) HAVERAGE
/STATISTICS DESCRIPTIVES EXTREME
/CINTERVAL 95
/MISSING LISTWISE
/NOTOTAL.
```

探索

备注

已创建输出		27-JUN-2022 20:24:24
注释		
输入	数据	/Users/jasmine/Documents/工作/我的科研课题/GDD早期家庭干预队列/GDD 数据集.sav
	活动数据集	数据集1
	过滤器	<无>
	权重	<无>
	拆分文件	<无>
	工作数据文件中的行数	306
缺失值处理	对缺失的定义	将因变量的用户定义缺失值视为缺失。
	使用的个案数	统计基于那些对任何所用因变量或因子都没有缺失值的个案。

语法		EXAMINE VARIABLES=DA_A_T1 BY 组别<br>/PLOT BOXPLOT STEMLEAF HISTOGRAM NPPLOT SPREADLEVEL(1)<br>/COMPARE GROUPS<br>/MESTIMATORS HUBER(1.339) ANDREW(1.34) HAMPEL(1.7,3.4,8.5) TUKEY(4.685)<br>/PERCENTILES(5,10,25,50,75,90,95) HAVERAGE<br>/STATISTICS DESCRIPTIVES EXTREME<br>/CINTERVAL 95<br>/MISSING LISTWISE<br>/NOTOTAL.
资源	处理程序时间	00:00:01.03
	耗用时间	00:00:01.00

警告

由于没有足够的唯一分布/水平对，因此无法为 基线大运动发育龄*组别 计算分布-水平图的回归斜率。

组别

个案处理摘要

		个案					
		有效		缺失		总计	
	组别	N	百分比	N	百分比	N	百分比
基线大运动发育龄	干预组	153	100.0%	0	0.0%	153	100.0%
	对照组	153	100.0%	0	0.0%	153	100.0%

描述

	组别		统计	标准误差
基线大运动发育龄	干预组	平均值	2.0425	.08435

		平均值的 95% 置信区间	下限	1.8758	
			上限	2.2091	
		5% 剪除后平均值		1.9880	
		中位数		2.0000	
		方差		1.089	
		标准偏差		1.04338	
		最小值		.50	
		最大值		5.00	
		范围		4.50	
		四分位距		2.00	
		偏度		.643	.196
		峰度		-.505	.390
	对照组	平均值		2.1340	.08436
		平均值的 95% 置信区间	下限	1.9673	
			上限	2.3007	
		5% 剪除后平均值		2.0606	
		中位数		2.0000	
		方差		1.089	
		标准偏差		1.04347	
		最小值		.50	
		最大值		5.00	
		范围		4.50	
		四分位距		2.00	
		偏度		.742	.196
		峰度		.044	.390

M 估计量

	组别	休伯 M 估计量[a]	图基双权[b]	汉佩尔 M 估计量[c]	安德鲁波[d]
基线大运动发育龄	干预组	1.9655	1.9687	2.0040	1.9688
	对照组	2.0509	2.0503	2.0841	2.0506

a. 加权常量为 1.339。
b. 加权常量为 4.685。
c. 加权常量为 1.700、3.400 和 8.500
d. 加权常量为 1.340*pi。

百分位数

			百分位数						
		组别	5	10	25	50	75	90	
加权平均（定义 1）	基线大运动发育龄	干预组	1.0000	1.0000	1.0000	2.0000	3.0000	3.5000	

		对照组	1.0000	1.0000	1.0000	2.0000	3.0000	3.5000	
图基枢纽	基线大运动发育龄	干预组			1.0000	2.0000	3.0000		
		对照组			1.0000	2.0000	3.0000		

极值

	组别			个案号	值
基线大运动发育龄	干预组	最大值	1	22	5.00
			2	40	5.00
			3	35	4.00
			4	37	4.00
			5	45	4.00[a]
		最小值	1	91	.50
			2	85	.50
			3	72	.50
			4	151	1.00
			5	150	1.00[b]
	对照组	最大值	1	177	5.00
			2	206	5.00
			3	209	5.00
			4	250	5.00
			5	297	5.00
		最小值	1	218	.50
			2	306	1.00
			3	304	1.00
			4	294	1.00
			5	293	1.00[b]

a. 在较大极值的表中，仅显示了不完整的个案列表（这些个案的值为 4.00）。

b. 在较小极值的表中，仅显示了不完整的个案列表（这些个案的值为 1.00）。

正态性检验

		柯尔莫戈洛夫-斯米诺夫(V)[a]			夏皮洛-威尔克		
	组别	统计	自由度	显著性	统计	自由度	显著性
基线大运动发育龄	干预组	.181	153	.000	.890	153	.000
	对照组	.159	153	.000	.896	153	.000

a. 里利氏显著性修正

方差齐性检验

		莱文统计	自由度 1	自由度 2	显著性
基线大运动发育龄	基于平均值	.003	1	304	.955
	基于中位数	.077	1	304	.782
	基于中位数并具有调整后自由度	.077	1	302.724	.782
	基于剪除后平均值	.012	1	304	.911

基线大运动发育龄

直方图(O)

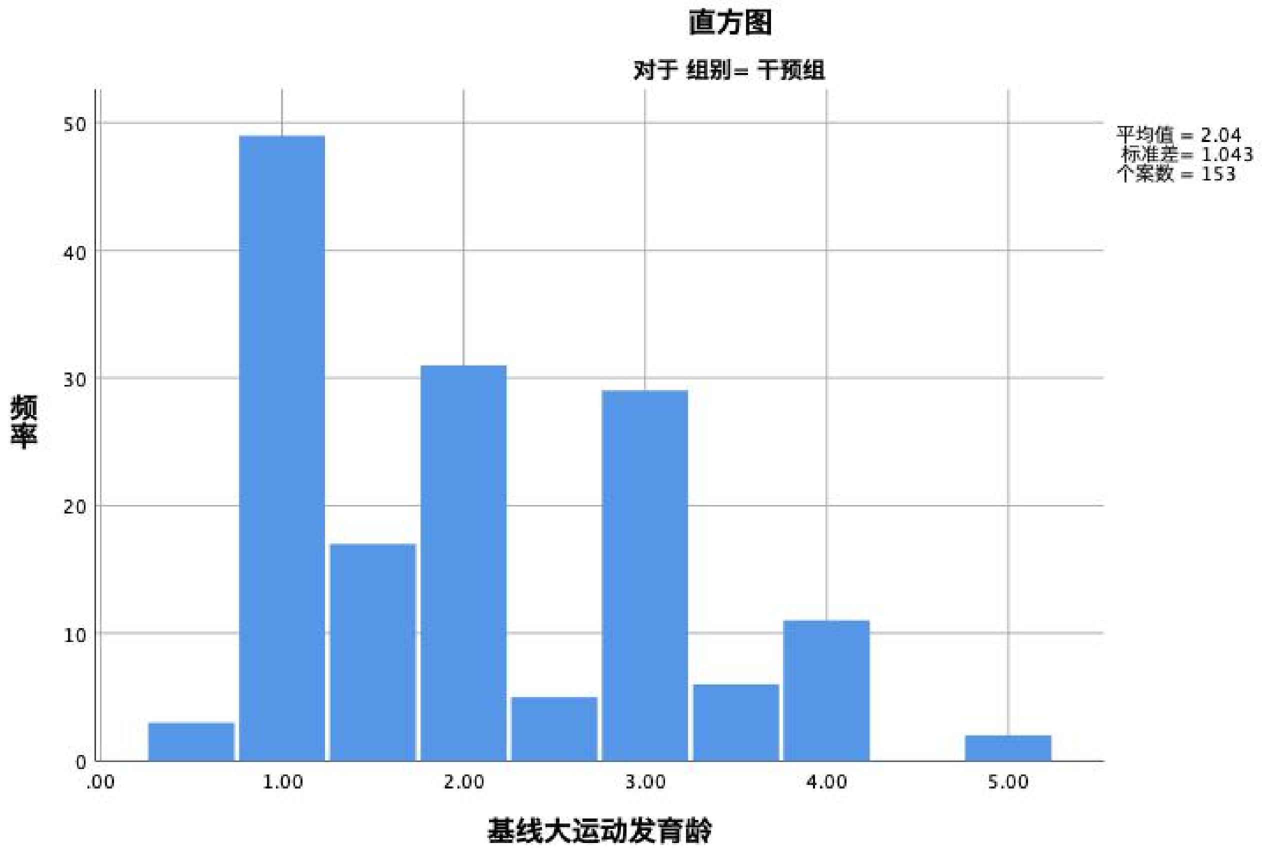

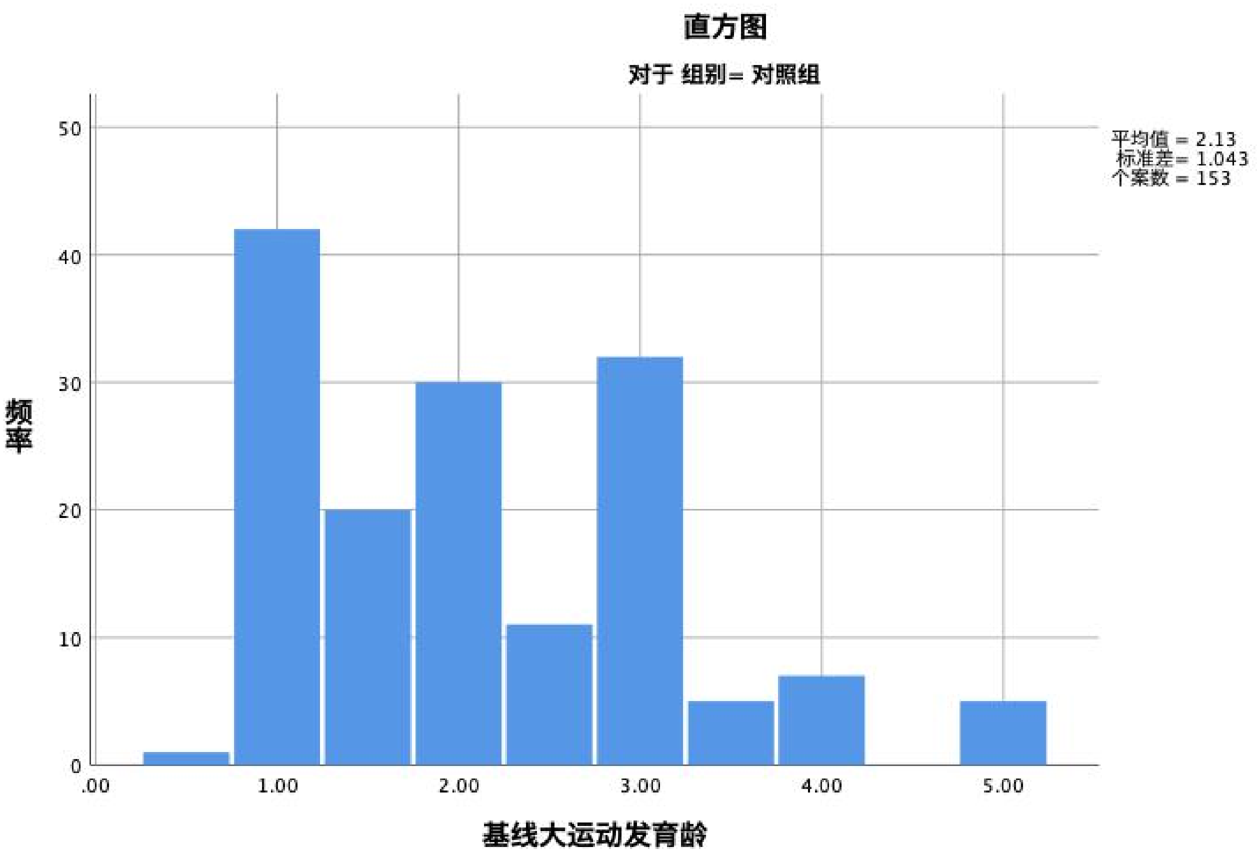

茎叶图

基线大运动发育龄 茎叶图：
组别= 干预组

频率	Stem &	叶
.00	0 .	
3.00	0 .	555
49.00	1 .	0000000000000000000000000000000000000000000000000
17.00	1 .	55555555555555555
31.00	2 .	0000000000000000000000000000000
5.00	2 .	55555
29.00	3 .	00000000000000000000000000000
6.00	3 .	555555
11.00	4 .	00000000000
.00	4 .	
2.00	5 .	00

主干宽度： 1.00
每个叶： 1 个案

基线大运动发育龄 茎叶图：
组别= 对照组

频率	Stem &	叶
.00	0 .	
1.00	0 .	5
42.00	1 .	000000000000000000000000000000000000000000
20.00	1 .	55555555555555555555
30.00	2 .	000000000000000000000000000000
11.00	2 .	55555555555
32.00	3 .	00000000000000000000000000000000
5.00	3 .	55555
7.00	4 .	0000000
.00	4 .	
5.00	5 .	00000

主干宽度： 1.00
每个叶： 1 个案

正态 Q-Q 图

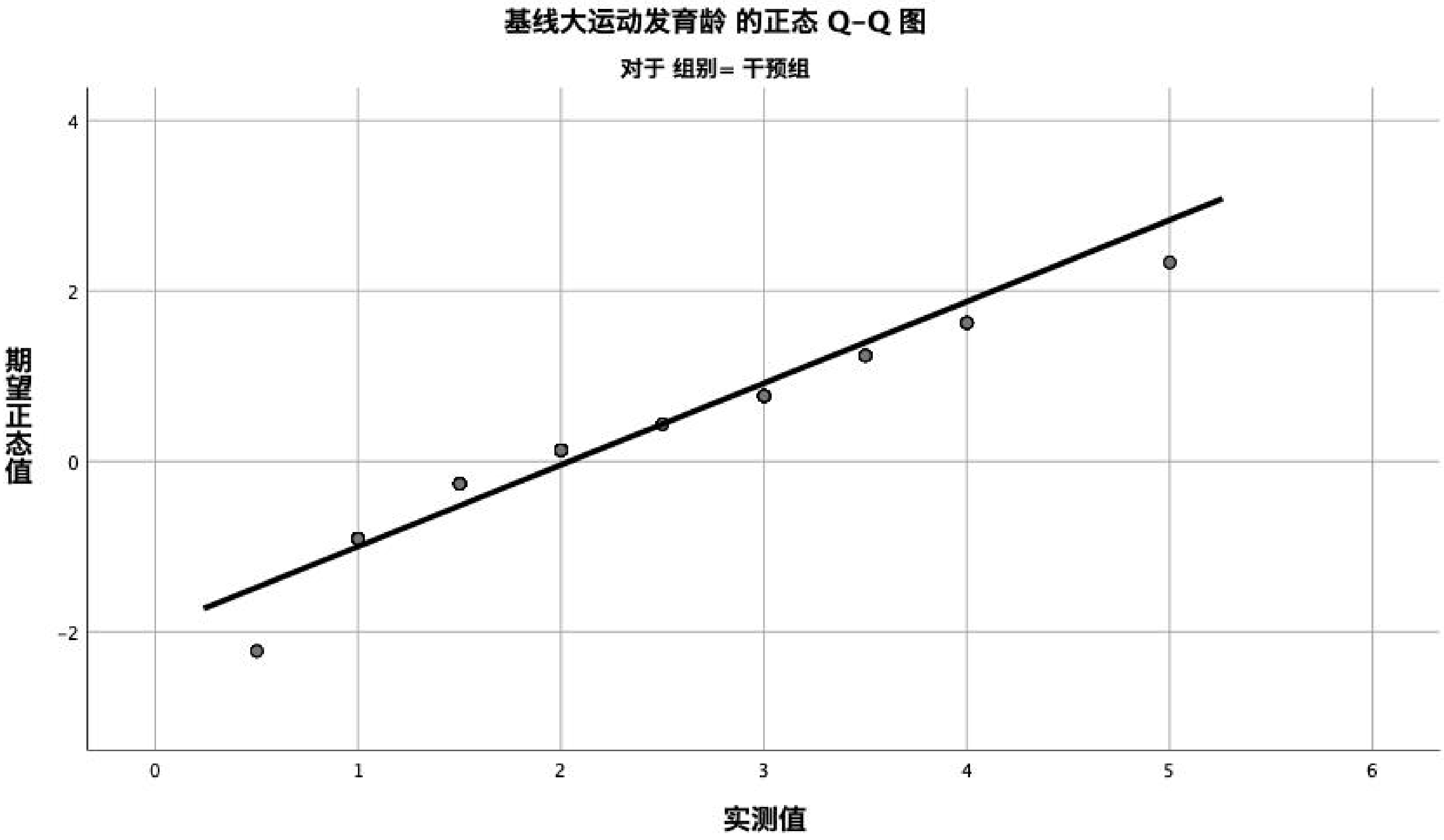
基线大运动发育龄 的正态 Q-Q 图
对于 组别= 干预组
期望正态值
实测值
4
2
0
-2
0
1
2
3
4
5
6

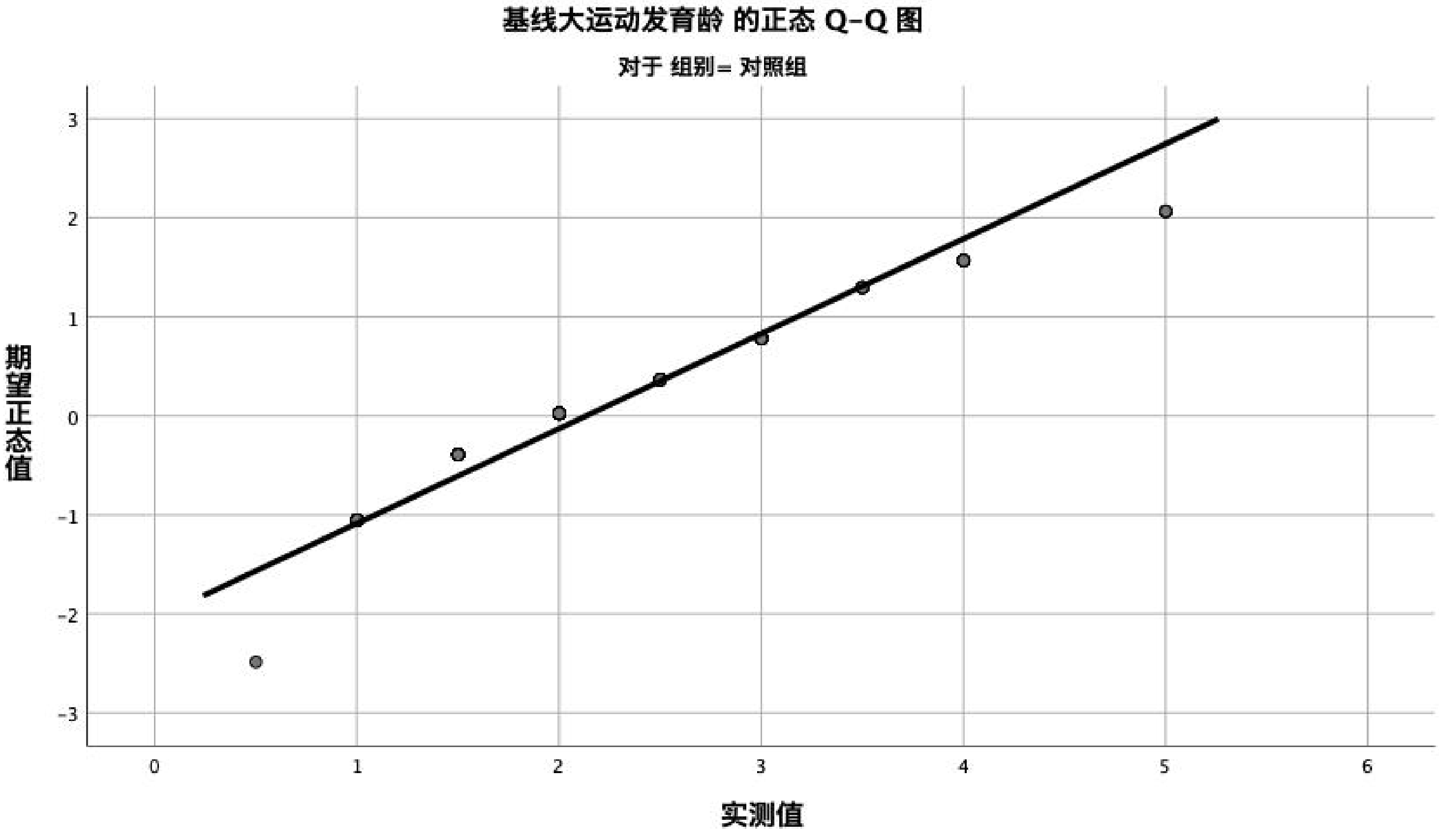
基线大运动发育龄 的正态 Q-Q 图
对于 组别= 对照组
期望正态值
实测值
3
2
1
0
-1
-2
-3
0
1
2
3
4
5
6

去趋势正态 Q-Q 图

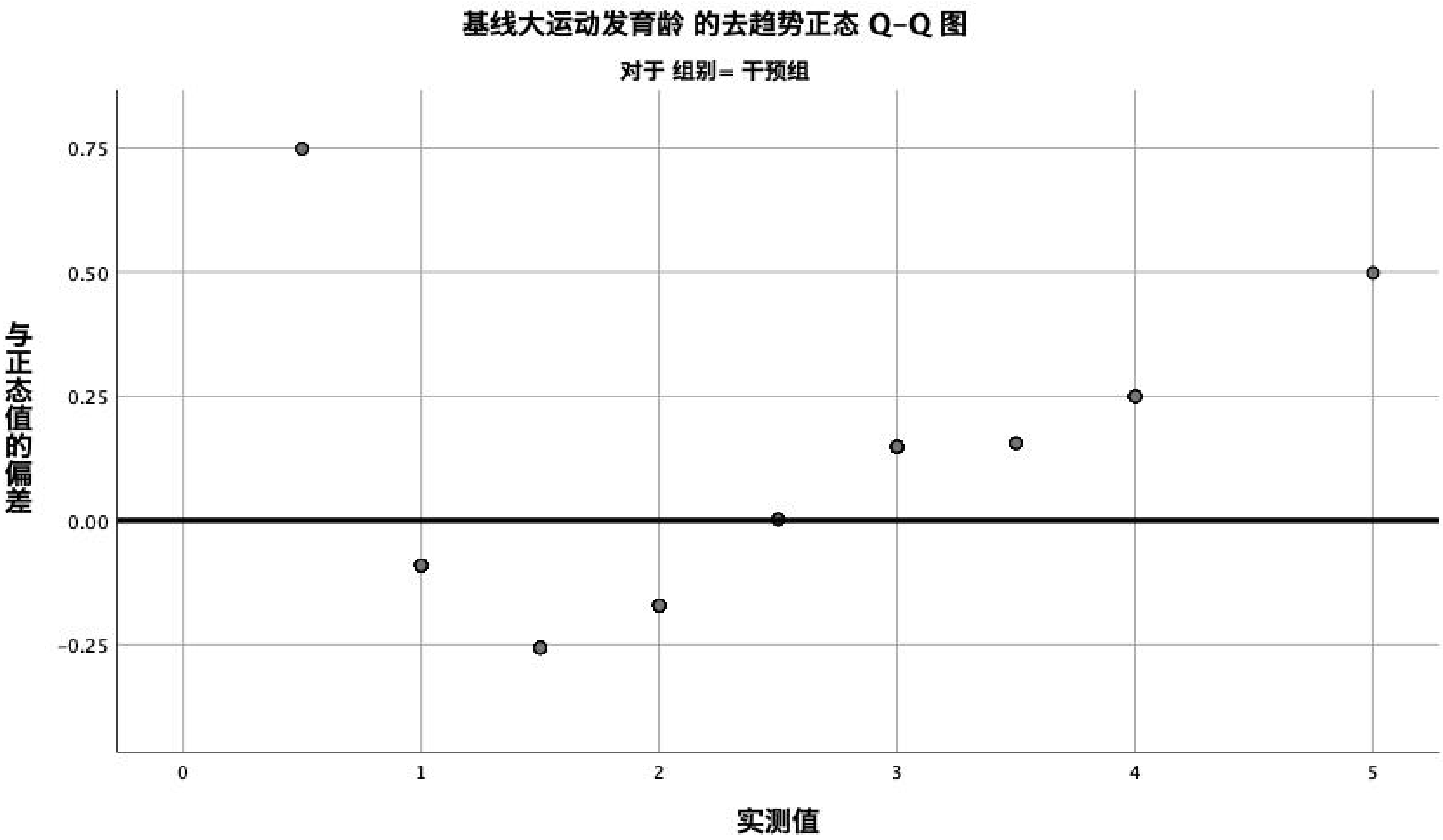
基线大运动发育龄 的去趋势正态 Q-Q 图
对于 组别= 干预组
与正态值的偏差
0.75
0.50
0.25
0.00
-0.25
0
1
2
3
4
5
实测值

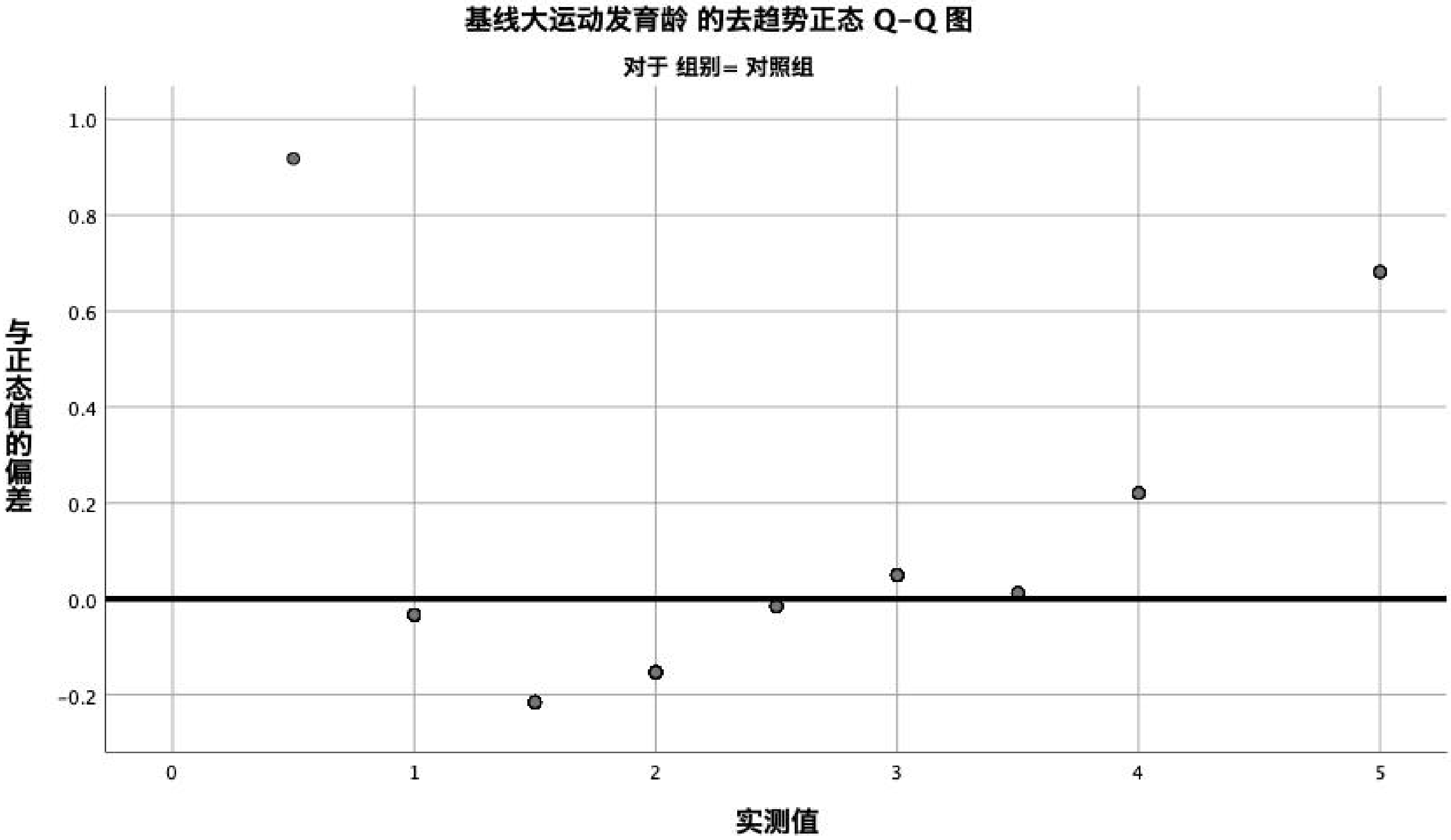
基线大运动发育龄 的去趋势正态 Q-Q 图
对于 组别= 对照组
与正态值的偏差
1.0
0.8
0.6
0.4
0.2
0.0
-0.2
0
1
2
3
4
5
实测值

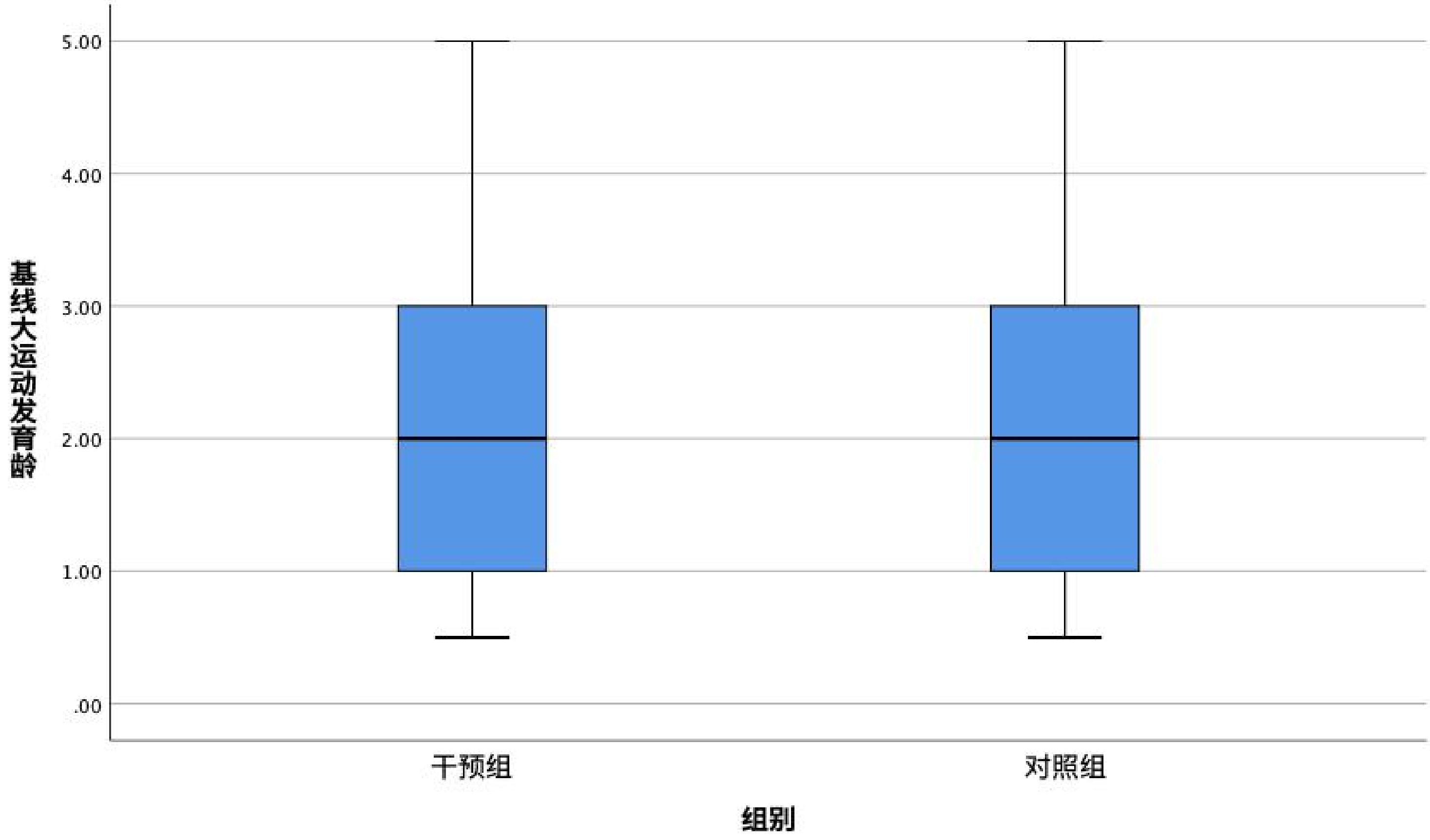

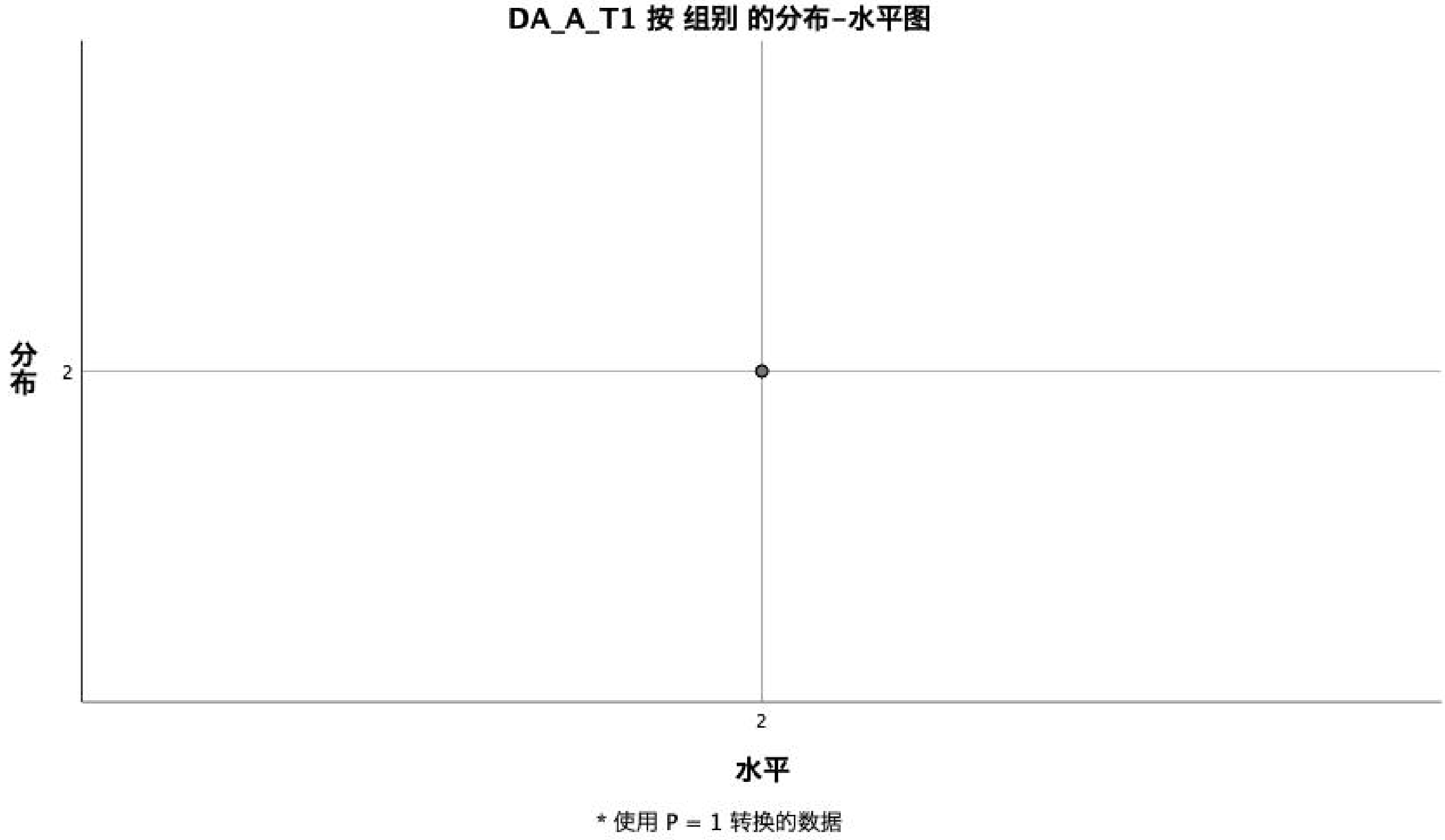


```
T-TEST GROUPS=组别(1 2)
  /MISSING=ANALYSIS
  /VARIABLES=DA_A_T1
```

/CRITERIA=CI(.95).

T-检验

备注

已创建输出		27-JUN-2022 20:27:59
注释		
输入	数据	/Users/jasmine/Documents/工作/我的科研课题/GDD早期家庭干预队列/GDD 数据集.sav
	活动数据集	数据集1
	过滤器	<无>
	权重	<无>
	拆分文件	<无>
	工作数据文件中的行数	306
缺失值处理	对缺失的定义	将用户定义的缺失值视为缺失。
	使用的个案数	每项分析的统计都基于符合以下条件的个案：对于该分析中的任何变量，都不具有缺失数据或超范围数据。
语法		T-TEST GROUPS=组别(1 2)<br>/MISSING=ANALYSIS<br>/VARIABLES=DA_A_T1<br>/CRITERIA=CI(.95).
资源	处理程序时间	00:00:00.00
	耗用时间	00:00:00.00

组统计

	组别	个案数	平均值	标准 偏差	标准 误差平均值
基线大运动发育龄	干预组	153	2.0425	1.04338	.08435
	对照组	153	2.1340	1.04347	.08436

独立样本检验

	莱文方差等同性检验	平均值等同性 t 检验		

		F	显著性	t	自由度	Sig.（双尾）	平均值差值	标准误差差值		
基线大运动发育龄	假定等方差	.003	.955	-.767	304	.444	-.09150	.11930		
	不假定等方差			-.767	304.000	.444	-.09150	.11930		

```
T-TEST GROUPS=组别(1 2)
  /MISSING=ANALYSIS
  /VARIABLES=DA_B_T1
  /CRITERIA=CI(.95).
```

T-检验

备注

已创建输出		27-JUN-2022 20:38:41
注释		
输入	数据	/Users/jasmine/Documents/工作/我的科研课题/GDD早期家庭干预队列/GDD 数据集.sav
	活动数据集	数据集1
	过滤器	<无>
	权重	<无>
	拆分文件	<无>
	工作数据文件中的行数	306
缺失值处理	对缺失的定义	将用户定义的缺失值视为缺失。
	使用的个案数	每项分析的统计都基于符合以下条件的个案：对于该分析中的任何变量，都不具有缺失数据或超范围数据。
语法		T-TEST GROUPS=组别(1 2) /MISSING=ANALYSIS /VARIABLES=DA_B_T1 /CRITERIA=CI(.95).
资源	处理程序时间	00:00:00.00
	耗用时间	00:00:00.00

组统计

	组别	个案数	平均值	标准 偏差	标准 误差平均值
基线个人社会发育龄	干预组	153	2.2124	1.13075	.09142
	对照组	153	2.3399	1.11605	.09023

独立样本检验

		莱文方差等同性检验		平均值等同性 t 检验						
		F	显著性	t	自由度	Sig.（双尾）	平均值差值			
基线个人社会发育龄	假定等方差	.040	.841	-.992	304	.322	-.12745			
	不假定等方差			-.992	303.948	.322	-.12745			

```
T-TEST GROUPS=组别(1 2)
  /MISSING=ANALYSIS
  /VARIABLES=DA_C_T1
  /CRITERIA=CI(.95).
```

T-检验

备注

已创建输出		27-JUN-2022 20:40:58
注释		
输入	数据	/Users/jasmine/Documents/工作/我的科研课题/GDD早期家庭干预队列/GDD 数据集.sav
	活动数据集	数据集1
	过滤器	<无>
	权重	<无>
	拆分文件	<无>
	工作数据文件中的行数	306
缺失值处理	对缺失的定义	将用户定义的缺失值视为缺失。

	使用的个案数	每项分析的统计都基于符合以下条件的个案：对于该分析中的任何变量，都不具有缺失数据或超范围数据。
语法		T-TEST GROUPS=组别(1 2)<br>/MISSING=ANALYSIS<br>/VARIABLES=DA_C_T1<br>/CRITERIA=CI(.95).
资源	处理程序时间	00:00:00.00
	耗用时间	00:00:00.00

组统计

	组别	个案数	平均值	标准 偏差	标准 误差平均值
基线语言发育龄	干预组	153	2.1895	1.12537	.09098
	对照组	153	2.4020	1.13128	.09146

独立样本检验

		莱文方差等同性检验		平均值等同性 t 检验						
		F	显著性	t	自由度	Sig.（双尾）	平均值差值	标准误差差值		
基线语言发育龄	假定等方差	.001	.977	-1.647	304	.101	-.21242	.12900		
	不假定等方差			-1.647	303.992	.101	-.21242	.12900		

```
T-TEST GROUPS=组别(1 2)
  /MISSING=ANALYSIS
  /VARIABLES=DA_D_T1
  /CRITERIA=CI(.95).
```

T-检验

备注

已创建输出	27-JUN-2022 20:42:51
注释	

输入	数据	/Users/jasmine/Documents/工作/我的科研课题/GDD早期家庭干预队列/GDD 数据集.sav
	活动数据集	数据集1
	过滤器	<无>
	权重	<无>
	拆分文件	<无>
	工作数据文件中的行数	306
缺失值处理	对缺失的定义	将用户定义的缺失值视为缺失。
	使用的个案数	每项分析的统计都基于符合以下条件的个案：对于该分析中的任何变量，都不具有缺失数据或超范围数据。
语法		T-TEST GROUPS=组别(1 2)<br>/MISSING=ANALYSIS<br>/VARIABLES=DA_D_T1<br>/CRITERIA=CI(.95).
资源	处理程序时间	00:00:00.00
	耗用时间	00:00:00.00

组统计

	组别	个案数	平均值	标准 偏差	标准 误差平均值
基线手眼协调发育龄	干预组	153	2.4510	1.30127	.10520
	对照组	153	2.5850	1.28349	.10376

独立样本检验

		莱文方差等同性检验		平均值等同性 t 检验						
		F	显著性	t	自由度	Sig.（双尾）	平均值差值			
基线手眼协调发育龄	假定等方差	.035	.852	-.907	304	.365	-.13399			
	不假定等方差			-.907	303.942	.365	-.13399			

```
T-TEST GROUPS=组别(1 2)
  /MISSING=ANALYSIS
  /VARIABLES=DA_E_T1
  /CRITERIA=CI(.95).
```

T-检验

备注

已创建输出		27-JUN-2022 20:44:43
注释		
输入	数据	/Users/jasmine/Documents/工作/我的科研课题/GDD早期家庭干预队列/GDD 数据集.sav
	活动数据集	数据集1
	过滤器	<无>
	权重	<无>
	拆分文件	<无>
	工作数据文件中的行数	306
缺失值处理	对缺失的定义	将用户定义的缺失值视为缺失。
	使用的个案数	每项分析的统计都基于符合以下条件的个案：对于该分析中的任何变量，都不具有缺失数据或超范围数据。
语法		T-TEST GROUPS=组别(1 2)<br>/MISSING=ANALYSIS<br>/VARIABLES=DA_E_T1<br>/CRITERIA=CI(.95).
资源	处理程序时间	00:00:00.00
	耗用时间	00:00:00.00

组统计

	组别	个案数	平均值	标准 偏差	标准 误差平均值
基线表现发育龄	干预组	153	2.6503	1.28642	.10400
	对照组	153	2.7974	1.31093	.10598

独立样本检验

	莱文方差等同性检验		平均值等同性 t 检验				
	F	显著性	t	自由度	Sig.（双尾）	平均值差值	标准误差差值

基线表现发育龄	假定等方差	.283	.595	-.990	304	.323	-.14706	.14849		
	不假定等方差			-.990	303.892	.323	-.14706	.14849		

```
GET DATA
  /TYPE=XLSX
  /FILE='/Users/jasmine/Documents/工作/我的科研课题/GDD早期家庭干预队列/GDD 数据集(t检验用）.xlsx'
  /SHEET=name '干预组-GDS-C'
  /CELLRANGE=FULL
  /READNAMES=ON
  /DATATYPEMIN PERCENTAGE=95.0
  /HIDDEN IGNORE=YES.
EXECUTE.
DATASET NAME 数据集1 WINDOW=FRONT.
T-TEST PAIRS=DQ_A_T2 WITH DQ_A_T1 (PAIRED)
  /CRITERIA=CI(.9500)
  /MISSING=ANALYSIS.
```

T-检验

备注

已创建输出		04-OCT-2022 20:06:56
注释		
输入	活动数据集	数据集1
	过滤器	<无>
	权重	<无>
	拆分文件	<无>
	工作数据文件中的行数	77
缺失值处理	对缺失的定义	将用户定义的缺失值视为缺失。
	使用的个案数	每项分析的统计都基于符合以下条件的个案：对于该分析中的任何变量，都不具有缺失数据或超范围数据。

语法		T-TEST PAIRS=DQ_A_T2 WITH DQ_A_T1 (PAIRED)<br>/CRITERIA=CI(.9500)<br>/MISSING=ANALYSIS.
资源	处理程序时间	00:00:00.00
	耗用时间	00:00:00.00

[数据集1]

配对样本统计

		平均值	个案数	标准 偏差	标准 误差平均值
配对 1	DQ_A_T2	85.037878787878780	77	14.922922983878700	1.700624905705597
	DQ_A_T1	45.433373758947845	77	18.289011838782564	2.084226332024804

配对样本相关性

		个案数	相关性	显著性
配对 1	DQ_A_T2 & DQ_A_T1	77	-.011	.923

配对样本检验

	配对差值							
	平均值	标准 偏差	标准 误差平均值	差值 95% 置信区间 下限	上限			
配对 1 DQ_A_T2 - DQ_A_T1	39.604505028930930	23.733743335958565	2.704711072110929	34.217606069606000	44.991403988255860			

```
T-TEST PAIRS=DQ_B_T2 WITH DQ_B_T1 (PAIRED)
  /CRITERIA=CI(.9500)
  /MISSING=ANALYSIS.
```

T-检验

备注

已创建输出		04-OCT-2022 20:09:08
注释		
输入	活动数据集	数据集1
	过滤器	<无>
	权重	<无>
	拆分文件	<无>
	工作数据文件中的行数	77
缺失值处理	对缺失的定义	将用户定义的缺失值视为缺失。
	使用的个案数	每项分析的统计都基于符合以下条件的个案：对于该分析中的任何变量，都不具有缺失数据或超范围数据。
语法		T-TEST PAIRS=DQ_B_T2 WITH DQ_B_T1 (PAIRED)<br>/CRITERIA=CI(.9500)<br>/MISSING=ANALYSIS.
资源	处理程序时间	00:00:00.00
	耗用时间	00:00:00.00

配对样本统计

		平均值	个案数	标准 偏差	标准 误差平均值
配对 1	DQ_B_T2	86.715367965367930	77	13.267031910645388	1.511918604445560
	DQ_B_T1	50.286576229018080	77	22.793244617074503	2.597531295947350

配对样本相关性

		个案数	相关性	显著性
配对 1	DQ_B_T2 & DQ_B_T1	77	-.105	.362

配对样本检验

		配对差值							
					差值 95% 置信区间				
		平均值	标准 偏差	标准 误差平均值	下限	上限			
配对 1	DQ_B_T2 - DQ_B_T1	36.428791736349850	27.554059668432320	3.140076523617808	30.174787332073260	42.682796140626440			

T-TEST PAIRS=DQ_C_T2 WITH DQ_C_T1 (PAIRED)

```
/CRITERIA=CI(.9500)
/MISSING=ANALYSIS.
```

T-检验

备注

已创建输出		04-OCT-2022 20:10:01
注释		
输入	活动数据集	数据集1
	过滤器	<无>
	权重	<无>
	拆分文件	<无>
	工作数据文件中的行数	77
缺失值处理	对缺失的定义	将用户定义的缺失值视为缺失。
	使用的个案数	每项分析的统计都基于符合以下条件的个案：对于该分析中的任何变量，都不具有缺失数据或超范围数据。
语法		T-TEST PAIRS=DQ_C_T2 WITH DQ_C_T1 (PAIRED) /CRITERIA=CI(.9500) /MISSING=ANALYSIS.
资源	处理程序时间	00:00:00.00
	耗用时间	00:00:00.00

配对样本统计

		平均值	个案数	标准 偏差	标准 误差平均值
配对 1	DQ_C_T2	89.258658008658000	77	15.826785259644325	1.803629571691968
	DQ_C_T1	48.814789939427090	77	22.408183510714572	2.553649510294387

配对样本相关性

		个案数	相关性	显著性
配对 1	DQ_C_T2 & DQ_C_T1	77	.092	.427

配对样本检验

		配对差值							
		平均值	标准 偏差	标准 误差平均值	差值 95% 置信区间 下限	上限			
配对 1	DQ_C_T2 - DQ_C_T1	40.443868069230920	26.220938431227943	2.988153258935411	34.492445069989990	46.395291068471840			

```
T-TEST PAIRS=DQ_D_T2 WITH DQ_D_T1 (PAIRED)
  /CRITERIA=CI(.9500)
  /MISSING=ANALYSIS.
```

T-检验

备注

已创建输出		04-OCT-2022 20:11:04
注释		
输入	活动数据集	数据集1
	过滤器	<无>
	权重	<无>
	拆分文件	<无>
	工作数据文件中的行数	77
缺失值处理	对缺失的定义	将用户定义的缺失值视为缺失。
	使用的个案数	每项分析的统计都基于符合以下条件的个案：对于该分析中的任何变量，都不具有缺失数据或超范围数据。
语法		T-TEST PAIRS=DQ_D_T2 WITH DQ_D_T1 (PAIRED) /CRITERIA=CI(.9500) /MISSING=ANALYSIS.
资源	处理程序时间	00:00:00.00
	耗用时间	00:00:00.00

配对样本统计

		平均值	个案数	标准 偏差	标准 误差平均值
配对 1	DQ_D_T2	84.199134199134180	77	14.343861875712944	1.634634768013671
	DQ_D_T1	54.527021085549160	77	24.088863453371722	2.745180765403747

配对样本相关性

		个案数	相关性	显著性
配对 1	DQ_D_T2 & DQ_D_T1	77	.012	.914

配对样本检验

		配对差值							
		平均值	标准 偏差	标准 误差平均值	差值 95% 置信区间 下限	上限			
配对 1	DQ_D_T2 - DQ_D_T1	29.672113113585020	27.881905118359384	3.177437980081166	23.343696919813940	36.000529307356100			

```
T-TEST PAIRS=DQ_E_T2 WITH DQ_E_T1 (PAIRED)
  /CRITERIA=CI(.9500)
  /MISSING=ANALYSIS.
```

T-检验

备注

已创建输出		04-OCT-2022 20:11:51
注释		
输入	活动数据集	数据集1
	过滤器	<无>
	权重	<无>
	拆分文件	<无>
	工作数据文件中的行数	77
缺失值处理	对缺失的定义	将用户定义的缺失值视为缺失。

	使用的个案数	每项分析的统计都基于符合以下条件的个案：对于该分析中的任何变量，都不具有缺失数据或超范围数据。
语法		T-TEST PAIRS=DQ_E_T2 WITH DQ_E_T1 (PAIRED)<br>/CRITERIA=CI(.9500)<br>/MISSING=ANALYSIS.
资源	处理程序时间	00:00:00.00
	耗用时间	00:00:00.00

配对样本统计

		平均值	个案数	标准 偏差	标准 误差平均值
配对 1	DQ_E_T2	85.037878787878810	77	16.228221716823363	1.849377501764006
	DQ_E_T1	57.617912301608136	77	23.397971253381330	2.666446292021374

配对样本相关性

		个案数	相关性	显著性
配对 1	DQ_E_T2 & DQ_E_T1	77	-.131	.255

配对样本检验

	配对差值							
	平均值	标准 偏差	标准 误差平均值	差值 95% 置信区间 下限	上限			
配对 1 DQ_E_T2 - DQ_E_T1	27.419966486270678	30.175817234481837	3.438853527182228	20.570896107603236	34.269036864938116			

```
T-TEST PAIRS=GQ_T2 WITH GQ_T1 (PAIRED)
  /CRITERIA=CI(.9500)
  /MISSING=ANALYSIS.
```

T-检验

备注

已创建输出		04-OCT-2022 20:12:48
注释		
输入	活动数据集	数据集1
	过滤器	<无>
	权重	<无>
	拆分文件	<无>
	工作数据文件中的行数	77
缺失值处理	对缺失的定义	将用户定义的缺失值视为缺失。
	使用的个案数	每项分析的统计都基于符合以下条件的个案：对于该分析中的任何变量，都不具有缺失数据或超范围数据。
语法		T-TEST PAIRS=GQ_T2 WITH GQ_T1 (PAIRED)<br>/CRITERIA=CI(.9500)<br>/MISSING=ANALYSIS.
资源	处理程序时间	00:00:00.00
	耗用时间	00:00:00.00

配对样本统计

		平均值	个案数	标准 偏差	标准 误差平均值
配对 1	GQ_T2	86.049783549783530	77	11.810015787701094	1.345876207163842
	GQ_T1	51.335934662910034	77	12.526848480166692	1.427566874022336

配对样本相关性

		个案数	相关性	显著性
配对 1	GQ_T2 & GQ_T1	77	-.153	.185

配对样本检验

		配对差值							
					差值 95% 置信区间				
		平均值	标准 偏差	标准 误差平均值	下限	上限	t		
配对 1	GQ_T2 - GQ_T1	34.713848886873480	18.481430516278390	2.106154475433830	30.519078706472214	38.908619067274740	16.482		

```
GET DATA
  /TYPE=XLSX
  /FILE='/Users/jasmine/Documents/工作/我的科研课题/GDD早期家庭干预队列/GDD 数据集(t检验用）.xlsx'
  /SHEET=name '对照组-GDS-C'
  /CELLRANGE=FULL
  /READNAMES=ON
  /DATATYPEMIN PERCENTAGE=95.0
  /HIDDEN IGNORE=YES.
EXECUTE.
DATASET NAME 数据集2 WINDOW=FRONT.
DATASET ACTIVATE 数据集2.
DATASET CLOSE 数据集1.
T-TEST PAIRS=DQ_A_T2 WITH DQ_A_T1 (PAIRED)
  /CRITERIA=CI(.9500)
  /MISSING=ANALYSIS.
```

T-检验

备注

已创建输出		04-OCT-2022 20:16:43
注释		
输入	活动数据集	数据集2
	过滤器	<无>
	权重	<无>
	拆分文件	<无>
	工作数据文件中的行数	69
缺失值处理	对缺失的定义	将用户定义的缺失值视为缺失。
	使用的个案数	每项分析的统计都基于符合以下条件的个案：对于该分析中的任何变量，都不具有缺失数据或超范围数据。
语法		T-TEST PAIRS=DQ_A_T2 WITH DQ_A_T1 (PAIRED) /CRITERIA=CI(.9500) /MISSING=ANALYSIS.
资源	处理程序时间	00:00:00.00
	耗用时间	00:00:00.00

配对样本统计

		平均值	个案数	标准 偏差	标准 误差平均值
配对 1	DQ_A_T2	79.196859903381650	69	17.088312109346090	2.057191031079509
	DQ_A_T1	49.229093360248040	69	24.935912823798233	3.001931137765323

配对样本相关性

		个案数	相关性	显著性
配对 1	DQ_A_T2 & DQ_A_T1	69	-.153	.210

配对样本检验

		配对差值							
					差值 95% 置信区间				
		平均值	标准 偏差	标准 误差平均值	下限	上限			
配对 1	DQ_A_T2 - DQ_A_T1	29.967766543133607	32.310383109466585	3.889713034161163	22.205965031288745	37.729568054978470			

```
T-TEST PAIRS=DQ_B_T2 WITH DQ_B_T1 (PAIRED)
  /CRITERIA=CI(.9500)
  /MISSING=ANALYSIS.
```

T-检验

备注

已创建输出		04-OCT-2022 20:17:31
注释		
输入	活动数据集	数据集2
	过滤器	<无>
	权重	<无>
	拆分文件	<无>
	工作数据文件中的行数	69
缺失值处理	对缺失的定义	将用户定义的缺失值视为缺失。

	使用的个案数	每项分析的统计都基于符合以下条件的个案：对于该分析中的任何变量，都不具有缺失数据或超范围数据。
语法		T-TEST PAIRS=DQ_B_T2 WITH DQ_B_T1 (PAIRED)<br>/CRITERIA=CI(.9500)<br>/MISSING=ANALYSIS.
资源	处理程序时间	00:00:00.00
	耗用时间	00:00:00.00

配对样本统计

		平均值	个案数	标准 偏差	标准 误差平均值
配对 1	DQ_B_T2	77.687198067632860	69	17.090585534999860	2.057464719366266
	DQ_B_T1	52.074964531269520	69	27.457225433250930	3.305461507150193

配对样本相关性

		个案数	相关性	显著性
配对 1	DQ_B_T2 & DQ_B_T1	69	-.059	.631

配对样本检验

		配对差值							
		平均值	标准 偏差	标准 误差平均值	差值 95% 置信区间				
					下限	上限			
配对 1	DQ_B_T2 - DQ_B_T1	25.612233536363340	33.185341909454250	3.995045695712579	17.640243970927852	33.584223101798830			

```
T-TEST PAIRS=DQ_C_T2 WITH DQ_C_T1 (PAIRED)
  /CRITERIA=CI(.9500)
  /MISSING=ANALYSIS.
```

T-检验

备注

已创建输出		04-OCT-2022 20:18:32
注释		
输入	活动数据集	数据集2
	过滤器	<无>
	权重	<无>
	拆分文件	<无>
	工作数据文件中的行数	69
缺失值处理	对缺失的定义	将用户定义的缺失值视为缺失。
	使用的个案数	每项分析的统计都基于符合以下条件的个案：对于该分析中的任何变量，都不具有缺失数据或超范围数据。
语法		T-TEST PAIRS=DQ_C_T2 WITH DQ_C_T1 (PAIRED) /CRITERIA=CI(.9500) /MISSING=ANALYSIS.
资源	处理程序时间	00:00:00.01
	耗用时间	00:00:00.00

配对样本统计

		平均值	个案数	标准 偏差	标准 误差平均值
配对 1	DQ_C_T2	78.260869565217400	69	17.646873814981706	2.124433958513495
	DQ_C_T1	52.963168804533770	69	28.534251998247070	3.435120268973288

配对样本相关性

		个案数	相关性	显著性
配对 1	DQ_C_T2 & DQ_C_T1	69	.065	.597

配对样本检验

	配对差值							
	平均值	标准 偏差	标准 误差平均值	差值 95% 置信区间 下限	上限			
配对 1 DQ_C_T2 - DQ_C_T1	25.297700760683625	32.564631954274620	3.920320998239450	17.474822007466230	33.120579513901020			

T-TEST PAIRS=DQ_D_T2 WITH DQ_D_T1 (PAIRED)

/CRITERIA=CI(.9500)
/MISSING=ANALYSIS.

T-检验

备注

已创建输出		04-OCT-2022 20:19:08
注释		
输入	活动数据集	数据集2
	过滤器	<无>
	权重	<无>
	拆分文件	<无>
	工作数据文件中的行数	69
缺失值处理	对缺失的定义	将用户定义的缺失值视为缺失。
	使用的个案数	每项分析的统计都基于符合以下条件的个案：对于该分析中的任何变量，都不具有缺失数据或超范围数据。
语法		T-TEST PAIRS=DQ_D_T2 WITH DQ_D_T1 (PAIRED) /CRITERIA=CI(.9500) /MISSING=ANALYSIS.
资源	处理程序时间	00:00:00.00
	耗用时间	00:00:00.00

配对样本统计

		平均值	个案数	标准 偏差	标准 误差平均值
配对 1	DQ_D_T2	80.766908212560410	69	16.582758018583310	1.996329470582031
	DQ_D_T1	59.843324598446210	69	31.552462009557430	3.798470055976895

配对样本相关性

		个案数	相关性	显著性
配对 1	DQ_D_T2 & DQ_D_T1	69	-.170	.163

配对样本检验

		配对差值							
		平均值	标准 偏差	标准 误差平均值	差值 95% 置信区间 下限	上限			
配对 1	DQ_D_T2 - DQ_D_T1	20.923583614114200	38.058087881622280	4.581655376442272	11.781032655906847	30.066134572321555			

```
T-TEST PAIRS=DQ_E_T2 WITH DQ_E_T1 (PAIRED)
  /CRITERIA=CI(.9500)
  /MISSING=ANALYSIS.
```

T-检验

备注

已创建输出		04-OCT-2022 20:20:18
注释		
输入	活动数据集	数据集2
	过滤器	<无>
	权重	<无>
	拆分文件	<无>
	工作数据文件中的行数	69
缺失值处理	对缺失的定义	将用户定义的缺失值视为缺失。
	使用的个案数	每项分析的统计都基于符合以下条件的个案：对于该分析中的任何变量，都不具有缺失数据或超范围数据。
语法		T-TEST PAIRS=DQ_E_T2 WITH DQ_E_T1 (PAIRED) /CRITERIA=CI(.9500) /MISSING=ANALYSIS.
资源	处理程序时间	00:00:00.00
	耗用时间	00:00:00.00

配对样本统计

		平均值	个案数	标准 偏差	标准 误差平均值
配对 1	DQ_E_T2	79.438405797101440	69	17.928322999053684	2.158316458638294
	DQ_E_T1	59.645541355342990	69	32.100823650328790	3.864485039900668

配对样本相关性

		个案数	相关性	显著性
配对 1	DQ_E_T2 & DQ_E_T1	69	.055	.655

配对样本检验

		配对差值							
					差值 95% 置信区间				
		平均值	标准 偏差	标准 误差平均值	下限	上限			
配对 1	DQ_E_T2 - DQ_E_T1	19.792864441758447	35.899646016368340	4.321809511157638	11.168827834686988	28.416901048829907			

```
T-TEST PAIRS=GQ_T2 WITH GQ_T1 (PAIRED)
  /CRITERIA=CI(.9500)
  /MISSING=ANALYSIS.
```

T-检验

备注

已创建输出		04-OCT-2022 20:20:45
注释		
输入	活动数据集	数据集2
	过滤器	<无>
	权重	<无>
	拆分文件	<无>
	工作数据文件中的行数	69
缺失值处理	对缺失的定义	将用户定义的缺失值视为缺失。

	使用的个案数	每项分析的统计都基于符合以下条件的个案：对于该分析中的任何变量，都不具有缺失数据或超范围数据。
语法		T-TEST PAIRS=GQ_T2 WITH GQ_T1 (PAIRED)<br>/CRITERIA=CI(.9500)<br>/MISSING=ANALYSIS.
资源	处理程序时间	00:00:00.00
	耗用时间	00:00:00.00

配对样本统计

		平均值	个案数	标准 偏差	标准 误差平均值
配对 1	GQ_T2	79.070048309178760	69	15.441677004882669	1.858959459307706
	GQ_T1	54.751218529968120	69	21.035551828765730	2.532382852035874

配对样本相关性

		个案数	相关性	显著性
配对 1	GQ_T2 & GQ_T1	69	-.089	.468

配对样本检验

		配对差值							
				标准 误差	差值 95% 置信区间				
		平均值	标准 偏差	平均值	下限	上限	t		
配对 1	GQ_T2 - GQ_T1	24.318829779210624	27.178747261038595	3.271936674822645	17.789781798997380	30.847877759423866	7.433		

```
GET DATA
  /TYPE=XLSX
  /FILE='/Users/jasmine/Documents/工作/我的科研课题/GDD早期家庭干预队列/GDD 数据集(t检验用）.xlsx'
  /SHEET=name 'GDS-C差值两组间比较'
  /CELLRANGE=FULL
  /READNAMES=ON
  /DATATYPEMIN PERCENTAGE=95.0
  /HIDDEN IGNORE=YES.
EXECUTE.
```

```
DATASET NAME 数据集3 WINDOW=FRONT.
DATASET CLOSE 数据集2.

GET DATA
  /TYPE=XLSX
  /FILE='/Users/jasmine/Documents/工作/我的科研课题/GDD早期家庭干预队列/GDD 数据集(t检验用）.xlsx'
  /SHEET=name 'GDS-C差值两组间比较'
  /CELLRANGE=FULL
  /READNAMES=ON
  /DATATYPEMIN PERCENTAGE=95.0
  /HIDDEN IGNORE=YES.
EXECUTE.
DATASET NAME 数据集4 WINDOW=FRONT.
DATASET ACTIVATE 数据集3.
T-TEST GROUPS=group(1 2)
  /MISSING=ANALYSIS
  /VARIABLES=DQ_AT2T1
  /CRITERIA=CI(.95).
```

T-检验

备注

已创建输出		04-OCT-2022 20:23:23
注释		
输入	活动数据集	数据集3
	过滤器	<无>
	权重	<无>
	拆分文件	<无>
	工作数据文件中的行数	146
缺失值处理	对缺失的定义	将用户定义的缺失值视为缺失。
	使用的个案数	每项分析的统计都基于符合以下条件的个案：对于该分析中的任何变量，都不具有缺失数据或超范围数据。
语法		T-TEST GROUPS=group(1 2) /MISSING=ANALYSIS /VARIABLES=DQ_AT2T1 /CRITERIA=CI(.95).

资源	处理程序时间	00:00:00.00
	耗用时间	00:00:00.00

组统计

	group	个案数	平均值	标准 偏差	标准 误差平均值
DQ_A(T2-T1)	1	77	39.604505028930960	23.733743335958565	2.704711072110929
	2	69	29.967766543133607	32.310383109466585	3.889713034161163

独立样本检验

		莱文方差等同性检验		平均值等同性 t 检验						
		F	显著性	t	自由度	Sig.（双尾）	平均值差值	标准误差差值		
DQ_A(T2-T1)	假定等方差	4.722	.031	2.068	144	.040	9.636738485797352	4.660100701517904		
	不假定等方差			2.034	123.766	.044	9.636738485797352	4.737650205716193		

```
T-TEST GROUPS=group(1 2)
  /MISSING=ANALYSIS
  /VARIABLES=DQ_BT2T1
  /CRITERIA=CI(.95).
```

T-检验

备注

已创建输出		04-OCT-2022 20:24:30
注释		
输入	活动数据集	数据集3
	过滤器	<无>
	权重	<无>
	拆分文件	<无>
	工作数据文件中的行数	146

缺失值处理	对缺失的定义	将用户定义的缺失值视为缺失。
	使用的个案数	每项分析的统计都基于符合以下条件的个案：对于该分析中的任何变量，都不具有缺失数据或超范围数据。
语法		T-TEST GROUPS=group(1 2)<br>/MISSING=ANALYSIS<br>/VARIABLES=DQ_BT2T1<br>/CRITERIA=CI(.95).
资源	处理程序时间	00:00:00.00
	耗用时间	00:00:00.00

组统计

	group	个案数	平均值	标准 偏差	标准 误差平均值
DQ_B(T2-T1)	1	77	36.428791736349930	27.554059668432338	3.140076523617809
	2	69	25.612233536363338	33.185341909454250	3.995045695712579

独立样本检验

		莱文方差等同性检验		平均值等同性 t 检验						
		F	显著性	t	自由度	Sig.（双尾）	平均值差值	标准误差差值		
DQ_B(T2-T1)	假定等方差	1.326	.251	2.150	144	.033	10.816558199986591	5.030096373214756		
	不假定等方差			2.129	132.668	.035	10.816558199986591	5.081384721215990		

```
T-TEST GROUPS=group(1 2)
  /MISSING=ANALYSIS
  /VARIABLES=DQ_CT2T1
  /CRITERIA=CI(.95).
```

T-检验

备注

已创建输出		04-OCT-2022 20:25:15
注释		
输入	活动数据集	数据集3
	过滤器	<无>
	权重	<无>
	拆分文件	<无>
	工作数据文件中的行数	146
缺失值处理	对缺失的定义	将用户定义的缺失值视为缺失。
	使用的个案数	每项分析的统计都基于符合以下条件的个案：对于该分析中的任何变量，都不具有缺失数据或超范围数据。
语法		T-TEST GROUPS=group(1 2)<br>/MISSING=ANALYSIS<br>/VARIABLES=DQ_CT2T1<br>/CRITERIA=CI(.95).
资源	处理程序时间	00:00:00.00
	耗用时间	00:00:00.00

组统计

	group	个案数	平均值	标准 偏差	标准 误差平均值
DQ_C(T2-T1)	1	77	40.443868069230895	26.220938431227943	2.988153258935411
	2	69	25.297700760683615	32.564631954274620	3.920320998239450

独立样本检验

		莱文方差等同性检验		平均值等同性 t 检验						
		F	显著性	t	自由度	Sig.（双尾）	平均值差值	标准误差差值		
DQ_C(T2-T1)	假定等方差	1.044	.309	3.109	144	.002	15.1461673085472880	4.871607532953687		
	不假定等方差			3.073	130.541	.003	15.1461673085472880	4.929297782455780		

```
T-TEST GROUPS=group(1 2)
  /MISSING=ANALYSIS
  /VARIABLES=DQ_DT2T1
  /CRITERIA=CI(.95).
```

T-检验

备注

已创建输出		04-OCT-2022 20:25:36
注释		
输入	活动数据集	数据集3
	过滤器	<无>
	权重	<无>
	拆分文件	<无>
	工作数据文件中的行数	146
缺失值处理	对缺失的定义	将用户定义的缺失值视为缺失。
	使用的个案数	每项分析的统计都基于符合以下条件的个案：对于该分析中的任何变量，都不具有缺失数据或超范围数据。
语法		T-TEST GROUPS=group(1 2) /MISSING=ANALYSIS /VARIABLES=DQ_DT2T1 /CRITERIA=CI(.95).
资源	处理程序时间	00:00:00.00
	耗用时间	00:00:00.00

组统计

	group	个案数	平均值	标准 偏差	标准 误差平均值
DQ_D(T2-T1)	1	77	29.672113113585063	27.881905118359388	3.177437980081166
	2	69	20.923583614114204	38.058087881622280	4.581655376442272

独立样本检验

		莱文方差等同性检验		平均值等同性 t 检验						
		F	显著性	t	自由度	Sig.（双尾）	平均值差值			
DQ_D(T2-T1)	假定等方差	3.826	.052	1.595	144	.113	8.748529499470859			
	不假定等方差			1.569	123.566	.119	8.748529499470859			

```
T-TEST GROUPS=group(1 2)
  /MISSING=ANALYSIS
  /VARIABLES=DQ_ET2T1
  /CRITERIA=CI(.95).
```

T-检验

备注

已创建输出		04-OCT-2022 20:25:58
注释		
输入	活动数据集	数据集3
	过滤器	<无>
	权重	<无>
	拆分文件	<无>
	工作数据文件中的行数	146
缺失值处理	对缺失的定义	将用户定义的缺失值视为缺失。
	使用的个案数	每项分析的统计都基于符合以下条件的个案：对于该分析中的任何变量，都不具有缺失数据或超范围数据。
语法		T-TEST GROUPS=group(1 2) /MISSING=ANALYSIS /VARIABLES=DQ_ET2T1 /CRITERIA=CI(.95).
资源	处理程序时间	00:00:00.00
	耗用时间	00:00:00.00

组统计

	group	个案数	平均值	标准 偏差	标准 误差平均值
DQ_E(T2-T1)	1	77	27.419966486270670	30.175817234481837	3.438853527182228
	2	69	19.792864441758454	35.899646016368330	4.321809511157637

独立样本检验

		莱文方差等同性检验		平均值等同性 t 检验						
		F	显著性	t	自由度	Sig.（双尾）	平均值差值	标准误差差值		
DQ_E(T2-T1)	假定等方差	1.535	.217	1.394	144	.165	7.627102044512220	5.470860830842157		
	不假定等方差			1.381	133.488	.170	7.627102044512220	5.523020100646590		

```
T-TEST GROUPS=group(1 2)
  /MISSING=ANALYSIS
  /VARIABLES=GQT2T1
  /CRITERIA=CI(.95).
```

T-检验

备注

已创建输出		04-OCT-2022 20:26:20
注释		
输入	活动数据集	数据集3
	过滤器	<无>
	权重	<无>
	拆分文件	<无>
	工作数据文件中的行数	146
缺失值处理	对缺失的定义	将用户定义的缺失值视为缺失。
	使用的个案数	每项分析的统计都基于符合以下条件的个案：对于该分析中的任何变量，都不具有缺失数据或超范围数据。

语法		T-TEST GROUPS=group(1 2)<br>/MISSING=ANALYSIS<br>/VARIABLES=GQT2T1<br>/CRITERIA=CI(.95).
资源	处理程序时间	00:00:00.00
	耗用时间	00:00:00.00

组统计

	group	个案数	平均值	标准 偏差	标准 误差平均值
GQ(T2-T1)	1	77	33.490905163929800	19.442336178552473	2.215659838629914
	2	69	24.318829779210635	27.178747261038590	3.271936674822644

独立样本检验

		莱文方差等同性检验		平均值等同性 t 检验						
		F	显著性	t	自由度	Sig.（双尾）	平均值差值	标准误差差值		
GQ(T2-T1)	假定等方差	3.862	.051	2.363	144	.019	9.172075384719168	3.881733676096324		
	不假定等方差			2.321	121.756	.022	9.172075384719168	3.951546295384504		

```
DATASET ACTIVATE 数据集4.
DATASET CLOSE 数据集3.

GET DATA
  /TYPE=XLSX
  /FILE='/Users/jasmine/Documents/工作/我的科研课题/GDD早期家庭干预队列/GDD 数据集(t检验用）.xlsx'
  /SHEET=name '干预组-PSI'
  /CELLRANGE=FULL
  /READNAMES=ON
  /DATATYPEMIN PERCENTAGE=95.0
  /HIDDEN IGNORE=YES.
EXECUTE.
DATASET NAME 数据集5 WINDOW=FRONT.
T-TEST PAIRS=Paternt_distress_T2 WITH Paternt_distress_T1 (PAIRED)
  /CRITERIA=CI(.9500)
  /MISSING=ANALYSIS.
```

T-检验

备注

已创建输出		04-OCT-2022 20:32:22
注释		
输入	活动数据集	数据集5
	过滤器	<无>
	权重	<无>
	拆分文件	<无>
	工作数据文件中的行数	31
缺失值处理	对缺失的定义	将用户定义的缺失值视为缺失。
	使用的个案数	每项分析的统计都基于符合以下条件的个案：对于该分析中的任何变量，都不具有缺失数据或超范围数据。
语法		T-TEST PAIRS=Paternt_distress_T2 WITH Paternt_distress_T1 (PAIRED) /CRITERIA=CI(.9500) /MISSING=ANALYSIS.
资源	处理程序时间	00:00:00.00
	耗用时间	00:00:00.00

[数据集5]

配对样本统计

		平均值	个案数	标准 偏差	标准 误差平均值
配对 1	Paternt_distress_T2	30.61	31	12.669	2.275
	Paternt_distress_T1	33.26	31	9.284	1.668

配对样本相关性

	个案数	相关性	显著性

配对 1	Paternt_distress_T2 & Paternt_distress_T1	31	.091	.626

配对样本检验

		配对差值							
					差值 95% 置信区间				
		平均值	标准 偏差	标准 误差平均值	下限	上限	t	自由度	
配对 1	Paternt_distress_T2 - Paternt_distress_T1	-2.645	15.010	2.696	-8.151	2.861	-.981	30	

```
T-TEST PAIRS=Dysfunctional_interaction_T2 WITH Dysfunctional_interaction_T1 (PAIRED)
  /CRITERIA=CI(.9500)
  /MISSING=ANALYSIS.
```

T-检验

备注

已创建输出		04-OCT-2022 20:33:32
注释		
输入	活动数据集	数据集5
	过滤器	<无>
	权重	<无>
	拆分文件	<无>
	工作数据文件中的行数	31
缺失值处理	对缺失的定义	将用户定义的缺失值视为缺失。
	使用的个案数	每项分析的统计都基于符合以下条件的个案：对于该分析中的任何变量，都不具有缺失数据或超范围数据。
语法		T-TEST PAIRS=Dysfunctional_interaction_T2 WITH Dysfunctional_interaction_T1 (PAIRED) /CRITERIA=CI(.9500) /MISSING=ANALYSIS.
资源	处理程序时间	00:00:00.00

耗用时间	00:00:00.00

配对样本统计

		平均值	个案数	标准 偏差	标准 误差平均值
配对 1	Dysfunctional_interaction_T2	16.68	31	4.764	.856
	Dysfunctional_interaction_T1	27.29	31	8.517	1.530

配对样本相关性

		个案数	相关性	显著性
配对 1	Dysfunctional_interaction_T2 & Dysfunctional_interaction_T1	31	-.072	.702

配对样本检验

		配对差值							
					差值 95% 置信区间				
		平均值	标准 偏差	标准 误差平均值	下限	上限	t	自由度	
配对 1	Dysfunctional_interaction_T2 - Dysfunctional_interaction_T1	-10.613	10.052	1.805	-14.300	-6.926	-5.878	30	

```
T-TEST PAIRS=Difficult_childT2T1 WITH Difficult_child_T1 (PAIRED)
  /CRITERIA=CI(.9500)
  /MISSING=ANALYSIS.
```

```
T-TEST PAIRS=Difficult_child_T2 WITH Difficult_child_T1 (PAIRED)
  /CRITERIA=CI(.9500)
  /MISSING=ANALYSIS.
```

T-检验

备注

已创建输出		04-OCT-2022 20:37:11
注释		
输入	活动数据集	数据集5
	过滤器	<无>
	权重	<无>
	拆分文件	<无>
	工作数据文件中的行数	31
缺失值处理	对缺失的定义	将用户定义的缺失值视为缺失。
	使用的个案数	每项分析的统计都基于符合以下条件的个案：对于该分析中的任何变量，都不具有缺失数据或超范围数据。
语法		T-TEST PAIRS=Difficult_child_T2 WITH Difficult_child_T1 (PAIRED)<br>/CRITERIA=CI(.9500)<br>/MISSING=ANALYSIS.
资源	处理程序时间	00:00:00.00
	耗用时间	00:00:00.00

配对样本统计

		平均值	个案数	标准 偏差	标准 误差平均值
配对 1	Difficult_child_T2	22.97	31	9.112	1.637
	Difficult_child_T1	30.42	31	8.797	1.580

配对样本相关性

		个案数	相关性	显著性
配对 1	Difficult_child_T2 & Difficult_child_T1	31	-.089	.635

配对样本检验

配对差值							
			差值 95% 置信区间				
平均值	标准 偏差	标准 误差平均值	下限	上限	t	自由度	

配对 1	Difficult_child_T2 - Difficult_child_T1	-7.452	13.216	2.374	-12.299	-2.604	-3.139	30	

```
T-TEST PAIRS=PSI_total_T2 WITH PSI_total_T1 (PAIRED)
  /CRITERIA=CI(.9500)
  /MISSING=ANALYSIS.
```

T-检验

备注

已创建输出		04-OCT-2022 20:37:52
注释		
输入	活动数据集	数据集5
	过滤器	<无>
	权重	<无>
	拆分文件	<无>
	工作数据文件中的行数	31
缺失值处理	对缺失的定义	将用户定义的缺失值视为缺失。
	使用的个案数	每项分析的统计都基于符合以下条件的个案：对于该分析中的任何变量，都不具有缺失数据或超范围数据。
语法		T-TEST PAIRS=PSI_total_T2 WITH PSI_total_T1 (PAIRED) /CRITERIA=CI(.9500) /MISSING=ANALYSIS.
资源	处理程序时间	00:00:00.00
	耗用时间	00:00:00.00

配对样本统计

		平均值	个案数	标准 偏差	标准 误差平均值
配对 1	PSI_total_T2	70.26	31	17.451	3.134
	PSI_total_T1	90.97	31	20.026	3.597

配对样本相关性

		个案数	相关性	显著性
配对 1	PSI_total_T2 & PSI_total_T1	31	-.237	.200

配对样本检验

		配对差值							
					差值 95% 置信区间				
		平均值	标准 偏差	标准 误差平均值	下限	上限	t	自由度	
配对 1	PSI_total_T2 - PSI_total_T1	-20.710	29.512	5.300	-31.535	-9.885	-3.907	30	

```
DATASET ACTIVATE  数据集4.
DATASET CLOSE  数据集5.

GET DATA
  /TYPE=XLSX
  /FILE='/Users/jasmine/Documents/工作/我的科研课题/GDD早期家庭干预队列/GDD  数据集(t检验用）.xlsx'
  /SHEET=name '对照组-PSI'
  /CELLRANGE=FULL
  /READNAMES=ON
  /DATATYPEMIN PERCENTAGE=95.0
  /HIDDEN IGNORE=YES.
EXECUTE.
DATASET NAME  数据集6 WINDOW=FRONT.
T-TEST PAIRS=Paternt_distress_T2 WITH Paternt_distress_T1 (PAIRED)
  /CRITERIA=CI(.9500)
  /MISSING=ANALYSIS.
```

T-检验

备注

已创建输出		04-OCT-2022 20:39:06
注释		
输入	活动数据集	数据集6
	过滤器	<无>
	权重	<无>

	拆分文件	<无>
	工作数据文件中的行数	30
缺失值处理	对缺失的定义	将用户定义的缺失值视为缺失。
	使用的个案数	每项分析的统计都基于符合以下条件的个案：对于该分析中的任何变量，都不具有缺失数据或超范围数据。
语法		T-TEST PAIRS=Paternt_distress_T2 WITH Paternt_distress_T1 (PAIRED)<br>/CRITERIA=CI(.9500)<br>/MISSING=ANALYSIS.
资源	处理程序时间	00:00:00.00
	耗用时间	00:00:00.00

[数据集6]

配对样本统计

		平均值	个案数	标准 偏差	标准 误差平均值
配对 1	Patarnt_distress_T2	25.73	30	9.924	1.812
	Patarnt_distress_T1	29.53	30	12.803	2.337

配对样本相关性

		个案数	相关性	显著性
配对 1	Patarnt_distress_T2 & Patarnt_distress_T1	30	.087	.646

配对样本检验

		配对差值							
					差值 95% 置信区间				
		平均值	标准 偏差	标准 误差平均值	下限	上限	t	自由度	
配对 1	Patarnt_distress_T2 - Patarnt_distress_T1	-3.800	15.497	2.829	-9.587	1.987	-1.343	29	

T-TEST PAIRS=Dysfunctional_interaction_T2 WITH Dysfunctional_interaction_T1 (PAIRED)
/CRITERIA=CI(.9500)

/MISSING=ANALYSIS.

T-检验

备注

已创建输出		04-OCT-2022 20:39:55
注释		
输入	活动数据集	数据集6
	过滤器	<无>
	权重	<无>
	拆分文件	<无>
	工作数据文件中的行数	30
缺失值处理	对缺失的定义	将用户定义的缺失值视为缺失。
	使用的个案数	每项分析的统计都基于符合以下条件的个案：对于该分析中的任何变量，都不具有缺失数据或超范围数据。
语法		T-TEST PAIRS=Dysfunctional_interaction_T2 WITH Dysfunctional_interaction_T1 (PAIRED)<br>/CRITERIA=CI(.9500)<br>/MISSING=ANALYSIS.
资源	处理程序时间	00:00:00.00
	耗用时间	00:00:00.00

配对样本统计

		平均值	个案数	标准 偏差	标准 误差平均值
配对 1	Dysfunctional_interaction_T2	23.47	30	6.339	1.157
	Dysfunctional_interaction_T1	29.93	30	9.738	1.778

配对样本相关性

		个案数	相关性	显著性

配对 1	Dysfunctional_interaction_T2 & Dysfunctional_interaction_T1	30	.279	.135

配对样本检验

		配对差值							
					差值 95% 置信区间				
		平均值	标准 偏差	标准 误差平均值	下限	上限	t	自由度	
配对 1	Dysfunctional_interaction_T2 - Dysfunctional_interaction_T1	-6.467	10.027	1.831	-10.211	-2.723	-3.533	29	

```
T-TEST PAIRS=Difficult_child_T2 WITH Difficult_child_T1 (PAIRED)
  /CRITERIA=CI(.9500)
  /MISSING=ANALYSIS.
```

T-检验

备注

已创建输出		04-OCT-2022 20:40:23
注释		
输入	活动数据集	数据集6
	过滤器	<无>
	权重	<无>
	拆分文件	<无>
	工作数据文件中的行数	30
缺失值处理	对缺失的定义	将用户定义的缺失值视为缺失。
	使用的个案数	每项分析的统计都基于符合以下条件的个案：对于该分析中的任何变量，都不具有缺失数据或超范围数据。

语法		T-TEST PAIRS=Difficult_child_T2 WITH Difficult_child_T1 (PAIRED)<br>/CRITERIA=CI(.9500)<br>/MISSING=ANALYSIS.
资源	处理程序时间	00:00:00.00
	耗用时间	00:00:00.00

配对样本统计

		平均值	个案数	标准 偏差	标准 误差平均值
配对 1	Difficult_child_T2	24.87	30	9.786	1.787
	Difficult_child_T1	28.20	30	8.704	1.589

配对样本相关性

		个案数	相关性	显著性
配对 1	Difficult_child_T2 & Difficult_child_T1	30	-.049	.795

配对样本检验

		配对差值							
					差值 95% 置信区间				
		平均值	标准 偏差	标准 误差平均值	下限	上限	t	自由度	
配对 1	Difficult_child_T2 - Difficult_child_T1	-3.333	13.415	2.449	-8.342	1.676	-1.361	29	

```
T-TEST PAIRS=PSI_total_T2 WITH PSI_total_T1 (PAIRED)
  /CRITERIA=CI(.9500)
  /MISSING=ANALYSIS.
```

T-检验

备注

已创建输出	04-OCT-2022 20:40:55

注释		
输入	活动数据集	数据集6
	过滤器	<无>
	权重	<无>
	拆分文件	<无>
	工作数据文件中的行数	30
缺失值处理	对缺失的定义	将用户定义的缺失值视为缺失。
	使用的个案数	每项分析的统计都基于符合以下条件的个案：对于该分析中的任何变量，都不具有缺失数据或超范围数据。
语法		T-TEST PAIRS=PSI_total_T2 WITH PSI_total_T1 (PAIRED)<br>/CRITERIA=CI(.9500)<br>/MISSING=ANALYSIS.
资源	处理程序时间	00:00:00.00
	耗用时间	00:00:00.00

配对样本统计

		平均值	个案数	标准 偏差	标准 误差平均值
配对 1	PSI_total_T2	74.07	30	17.453	3.187
	PSI_total_T1	87.67	30	23.332	4.260

配对样本相关性

		个案数	相关性	显著性
配对 1	PSI_total_T2 & PSI_total_T1	30	.357	.053

配对样本检验

		配对差值							
					差值 95% 置信区间				
		平均值	标准 偏差	标准 误差平均值	下限	上限	t	自由度	
配对 1	PSI_total_T2 - PSI_total_T1	-13.600	23.633	4.315	-22.425	-4.775	-3.152	29	

DATASET ACTIVATE 数据集4.
DATASET CLOSE 数据集6.

```
GET DATA
  /TYPE=XLSX
  /FILE='/Users/jasmine/Documents/工作/我的科研课题/GDD早期家庭干预队列/GDD 数据集(t检验用）.xlsx'
  /SHEET=name 'PSI-差值两组间比较'
  /CELLRANGE=FULL
  /READNAMES=ON
  /DATATYPEMIN PERCENTAGE=95.0
  /HIDDEN IGNORE=YES.
EXECUTE.
DATASET NAME 数据集7 WINDOW=FRONT.
DATASET ACTIVATE 数据集7.
DATASET CLOSE 数据集4.

GET DATA
  /TYPE=XLSX
  /FILE='/Users/jasmine/Documents/工作/我的科研课题/GDD早期家庭干预队列/GDD 数据集(t检验用）.xlsx'
  /SHEET=name 'PSI-差值两组间比较'
  /CELLRANGE=FULL
  /READNAMES=ON
  /DATATYPEMIN PERCENTAGE=95.0
  /HIDDEN IGNORE=YES.
EXECUTE.
DATASET NAME 数据集8 WINDOW=FRONT.
DATASET ACTIVATE 数据集7.
T-TEST GROUPS=group(1 2)
  /MISSING=ANALYSIS
  /VARIABLES=Paternt_distressT2T1
  /CRITERIA=CI(.95).
```

T-检验

备注

已创建输出		04-OCT-2022 20:42:51
注释		
输入	活动数据集	数据集7
	过滤器	<无>
	权重	<无>
	拆分文件	<无>

	工作数据文件中的行数	61
缺失值处理	对缺失的定义	将用户定义的缺失值视为缺失。
	使用的个案数	每项分析的统计都基于符合以下条件的个案：对于该分析中的任何变量，都不具有缺失数据或超范围数据。
语法		T-TEST GROUPS=group(1 2) /MISSING=ANALYSIS /VARIABLES=Paternt_distressT2T1 /CRITERIA=CI(.95).
资源	处理程序时间	00:00:00.00
	耗用时间	00:00:00.00

组统计

	group	个案数	平均值	标准 偏差	标准 误差平均值
Paternt_distress(T2-T1)	1	31	-2.65	15.010	2.696
	2	30	-3.80	15.497	2.829

独立样本检验

		莱文方差等同性检验		平均值等同性 t 检验						
		F	显著性	t	自由度	Sig.（双尾）	平均值差值			
Paternt_distress(T2-T1)	假定等方差	.125	.724	.296	59	.769	1.155			
	不假定等方差			.295	58.750	.769	1.155			

```
T-TEST GROUPS=group(1 2)
  /MISSING=ANALYSIS
  /VARIABLES=Dysfunctional_interactionT2T1
  /CRITERIA=CI(.95).
```

T-检验

备注

已创建输出		04-OCT-2022 20:43:17
注释		
输入	活动数据集	数据集7
	过滤器	<无>
	权重	<无>
	拆分文件	<无>
	工作数据文件中的行数	61
缺失值处理	对缺失的定义	将用户定义的缺失值视为缺失。
	使用的个案数	每项分析的统计都基于符合以下条件的个案：对于该分析中的任何变量，都不具有缺失数据或超范围数据。
语法		T-TEST GROUPS=group(1 2)<br>/MISSING=ANALYSIS<br><br>/VARIABLES=Dysfunctional_interactionT2T1<br>/CRITERIA=CI(.95).
资源	处理程序时间	00:00:00.00
	耗用时间	00:00:00.00

组统计

	group	个案数	平均值	标准 偏差	标准 误差平均值
Dysfunctional_interaction(T2-T1)	1	31	-10.61	10.052	1.805
	2	30	-6.47	10.027	1.831

独立样本检验

		莱文方差等同性检验		平均值等同性 t 检验						
		F	显著性	t	自由度	Sig.（双尾）	平均值差值			
Dysfunctional_interaction(T2-T1)	假定等方差	.034	.854	-1.613	59	.112	-4.146			
	不假定等方差			-1.613	58.944	.112	-4.146			

```
T-TEST GROUPS=group(1 2)
  /MISSING=ANALYSIS
  /VARIABLES=Difficult_childT2T1
```

/CRITERIA=CI(.95).

T-检验

备注

已创建输出		04-OCT-2022 20:43:43
注释		
输入	活动数据集	数据集7
	过滤器	<无>
	权重	<无>
	拆分文件	<无>
	工作数据文件中的行数	61
缺失值处理	对缺失的定义	将用户定义的缺失值视为缺失。
	使用的个案数	每项分析的统计都基于符合以下条件的个案：对于该分析中的任何变量，都不具有缺失数据或超范围数据。
语法		T-TEST GROUPS=group(1 2)<br>/MISSING=ANALYSIS<br>/VARIABLES=Difficult_childT2T1<br>/CRITERIA=CI(.95).
资源	处理程序时间	00:00:00.00
	耗用时间	00:00:00.00

组统计

	group	个案数	平均值	标准 偏差	标准 误差平均值
Difficult_child(T2-T1)	1	31	-7.45	13.216	2.374
	2	30	-3.33	13.415	2.449

独立样本检验

莱文方差等同性检验		平均值等同性 t 检验					
F	显著性	t	自由度	Sig.（双尾）	平均值差值		

Difficult_child(T2-T1)	假定等方差	.049	.825	-1.208	59	.232	-4.118			
	不假定等方差			-1.207	58.863	.232	-4.118			

```
T-TEST GROUPS=group(1 2)
  /MISSING=ANALYSIS
  /VARIABLES=PSI_totalT2T1
  /CRITERIA=CI(.95).
```

T-检验

备注

已创建输出		04-OCT-2022 20:44:17
注释		
输入	活动数据集	数据集7
	过滤器	<无>
	权重	<无>
	拆分文件	<无>
	工作数据文件中的行数	61
缺失值处理	对缺失的定义	将用户定义的缺失值视为缺失。
	使用的个案数	每项分析的统计都基于符合以下条件的个案：对于该分析中的任何变量，都不具有缺失数据或超范围数据。
语法		T-TEST GROUPS=group(1 2) /MISSING=ANALYSIS /VARIABLES=PSI_totalT2T1 /CRITERIA=CI(.95).
资源	处理程序时间	00:00:00.00
	耗用时间	00:00:00.00

组统计

	group	个案数	平均值	标准 偏差	标准 误差平均值
PSI_total(T2-T1)	1	31	-20.71	29.512	5.300

	2	30	-13.60	23.633	4.315

独立样本检验

		莱文方差等同性检验		平均值等同性 t 检验						
		F	显著性	t	自由度	Sig.（双尾）	平均值差值	标准误差差值		
PSI_total(T2-T1)	假定等方差	.496	.484	-1.036	59	.304	-7.110	6.860		
	不假定等方差			-1.040	57.028	.303	-7.110	6.835		

```
DATASET ACTIVATE 数据集8.
DATASET CLOSE 数据集7.

GET DATA
  /TYPE=XLSX
  /FILE='/Users/jasmine/Documents/工作/我的科研课题/GDD早期家庭干预队列/GDD 数据集(回归分析用).xlsx'
  /SHEET=name '回归分析各变量'
  /CELLRANGE=FULL
  /READNAMES=ON
  /DATATYPEMIN PERCENTAGE=95.0
  /HIDDEN IGNORE=YES.
EXECUTE.
DATASET NAME 数据集9 WINDOW=FRONT.
REGRESSION
  /MISSING LISTWISE
  /STATISTICS COEFF OUTS R ANOVA
  /CRITERIA=PIN(.05) POUT(.10)
  /NOORIGIN
  /DEPENDENT DQ_AT2T1
  /METHOD=STEPWISE group 性别 基线月龄 出生胎龄 出生体重 父亲年龄 母亲年龄 父母受教育程度 家庭年收入 PSI_totalT2T1
    Difficult_childT2T1 Dysfunctional_interactionT2T1 Paternt_distressT2T1 DQ_A_T1
  /RESIDUALS DURBIN HISTOGRAM(ZRESID) NORMPROB(ZRESID).
```

回归

备注

已创建输出		04-OCT-2022 21:08:01
注释		
输入	活动数据集	数据集9
	过滤器	<无>
	权重	<无>
	拆分文件	<无>
	工作数据文件中的行数	146
缺失值处理	对缺失的定义	将用户定义的缺失值视为缺失。
	使用的个案数	统计基于那些对于任何所用变量都不具有缺失值的个案。
语法		REGRESSION<br>/MISSING LISTWISE<br>/STATISTICS COEFF OUTS R ANOVA<br>/CRITERIA=PIN(.05) POUT(.10)<br>/NOORIGIN<br>/DEPENDENT DQ_AT2T1<br>/METHOD=STEPWISE group 性别 基线月龄 出生胎龄 出生体重 父亲年龄 母亲年龄 父母受教育程度 家庭年收入 PSI_totalT2T1 Difficult_childT2T1 Dysfunctional_interactionT2T1 Paternt_distressT2T1 DQ_A_T1<br>/RESIDUALS DURBIN HISTOGRAM(ZRESID) NORMPROB(ZRESID).
资源	处理程序时间	00:00:00.80
	耗用时间	00:00:00.00
	所需内存量	16352 字节
	残差图需要更多内存	432 字节

[数据集9]

输入/除去的变量[a]

模型	输入的变量	除去的变量	方法

1	DQ_A_T1	.	步进（条件：要输入的 F 的概率 <= .050，要除去的 F 的概率 >= .100）。
2	group	.	步进（条件：要输入的 F 的概率 <= .050，要除去的 F 的概率 >= .100）。
3	Dysfunctional_interaction(T2-T1)	.	步进（条件：要输入的 F 的概率 <= .050，要除去的 F 的概率 >= .100）。
4	基线月龄	.	步进（条件：要输入的 F 的概率 <= .050，要除去的 F 的概率 >= .100）。

a. 因变量：DQ_A(T2-T1)

模型摘要[e]

模型	R	R 方	调整后 R 方	标准估算的错误	德宾-沃森
1	.809[a]	.654	.649	17.442004659642915	
2	.827[b]	.683	.672	16.838812622002570	
3	.842[c]	.709	.694	16.273052447353230	
4	.854[d]	.729	.710	15.848181779820324	1.789

a. 预测变量：(常量), DQ_A_T1
b. 预测变量：(常量), DQ_A_T1, group
c. 预测变量：(常量), DQ_A_T1, group, Dysfunctional_interaction(T2-T1)
d. 预测变量 :(常量), DQ_A_T1, group, Dysfunctional_interaction(T2-T1), 基线月龄
e. 因变量：DQ_A(T2-T1)

ANOVA[a]

模型		平方和	自由度	均方	F	显著性
1	回归	33995.438	1	33995.438	111.745	.000[b]

	残差	17949.188	59	304.224		
	总计	51944.627	60			
2	回归	35498.981	2	17749.491	62.598	.000[c]
	残差	16445.645	58	283.546		
	总计	51944.627	60			
3	回归	36850.329	3	12283.443	46.385	.000[d]
	残差	15094.297	57	264.812		
	总计	51944.627	60			
4	回归	37879.394	4	9469.849	37.704	.000[e]
	残差	14065.232	56	251.165		
	总计	51944.627	60			

a. 因变量：DQ_A(T2-T1)
b. 预测变量：(常量), DQ_A_T1
c. 预测变量：(常量), DQ_A_T1, group
d. 预测变量：(常量), DQ_A_T1, group, Dysfunctional_interaction(T2-T1)
e. 预测变量：(常量), DQ_A_T1, group, Dysfunctional_interaction(T2-T1), 基线月龄

系数[a]

模型		未标准化系数 B	标准错误	标准化系数 Beta	t	显著性
1	(常量)	88.791	5.189		17.111	.000
	DQ_A_T1	-1.132	.107	-.809	-10.571	.000
2	(常量)	103.108	7.985		12.913	.000
	DQ_A_T1	-1.120	.103	-.801	-10.822	.000
	group	-9.943	4.318	-.170	-2.303	.025
3	(常量)	111.651	8.593		12.993	.000
	DQ_A_T1	-1.155	.101	-.826	-11.412	.000
	group	-11.871	4.259	-.203	-2.787	.007
	Dysfunctional_interaction(T2-T1)	.482	.214	.167	2.259	.028
4	(常量)	134.064	13.880		9.659	.000
	DQ_A_T1	-1.161	.099	-.830	-11.773	.000
	group	-13.342	4.211	-.229	-3.168	.002
	Dysfunctional_interaction(T2-T1)	.497	.208	.172	2.390	.020
	基线月龄	-4.238	2.094	-.143	-2.024	.048

a. 因变量：DQ_A(T2-T1)

排除的变量[a]

模型	输入 Beta	t	显著性	偏相关	共线性统计 容差

1	group	-.170[b]	-2.303	.025	-.289	.998
	性别	-.084[b]	-1.061	.293	-.138	.937
	基线月龄	-.104[b]	-1.372	.175	-.177	.999
	出生胎龄	-.014[b]	-.185	.854	-.024	.987
	出生体重	.016[b]	.209	.835	.027	.975
	父亲年龄	.010[b]	.128	.898	.017	.984
	母亲年龄	-.034[b]	-.440	.662	-.058	.987
	父母受教育程度	-.110[b]	-1.452	.152	-.187	.996
	家庭年收入	-.033[b]	-.426	.672	-.056	.973
	PSI_total(T2-T1)	.016[b]	.208	.836	.027	.967
	Difficult_child(T2-T1)	.048[b]	.626	.534	.082	.998
	Dysfunctional_interaction(T2-T1)	.126[b]	1.643	.106	.211	.974
	Paternt_distress(T2-T1)	-.103[b]	-1.315	.194	-.170	.935
2	性别	-.068[c]	-.879	.383	-.116	.928
	基线月龄	-.137[c]	-1.864	.067	-.240	.970
	出生胎龄	-.021[c]	-.280	.781	-.037	.985
	出生体重	-.015[c]	-.191	.849	-.025	.945
	父亲年龄	.016[c]	.219	.828	.029	.982
	母亲年龄	-.036[c]	-.479	.634	-.063	.987
	父母受教育程度	-.118[c]	-1.619	.111	-.210	.994
	家庭年收入	-.015[c]	-.194	.847	-.026	.962
	PSI_total(T2-T1)	.039[c]	.511	.611	.068	.951
	Difficult_child(T2-T1)	.077[c]	1.029	.308	.135	.973
	Dysfunctional_interaction(T2-T1)	.167[c]	2.259	.028	.287	.935
	Paternt_distress(T2-T1)	-.113[c]	-1.493	.141	-.194	.933
3	性别	-.074[d]	-1.004	.320	-.133	.927
	基线月龄	-.143[d]	-2.024	.048	-.261	.969
	出生胎龄	.015[d]	.197	.845	.026	.940
	出生体重	.029[d]	.378	.707	.051	.885
	父亲年龄	.039[d]	.534	.595	.071	.964
	母亲年龄	-.029[d]	-.401	.690	-.053	.985
	父母受教育程度	-.094[d]	-1.307	.197	-.172	.967
	家庭年收入	-.013[d]	-.171	.865	-.023	.961
	PSI_total(T2-T1)	-.112[d]	-1.184	.242	-.156	.564
	Difficult_child(T2-T1)	.025[d]	.318	.752	.042	.862
	Paternt_distress(T2-T1)	-.144[d]	-1.971	.054	-.255	.908
4	性别	-.076[e]	-1.046	.300	-.140	.927
	出生胎龄	-.003[e]	-.036	.972	-.005	.927
	出生体重	.007[e]	.092	.927	.012	.865
	父亲年龄	.029[e]	.406	.687	.055	.959
	母亲年龄	-.043[e]	-.614	.542	-.082	.975
	父母受教育程度	-.070[e]	-.966	.338	-.129	.932
	家庭年收入	-.034[e]	-.471	.640	-.063	.942
	PSI_total(T2-T1)	-.082[e]	-.874	.386	-.117	.548

Difficult_child(T2-T1)	.027[e]	.363	.718	.049	.862
Paternt_distress(T2-T1)	-.117[e]	-1.580	.120	-.208	.862

a. 因变量：DQ_A(T2-T1)
b. 模型中的预测变量：(常量), DQ_A_T1
c. 模型中的预测变量：(常量), DQ_A_T1, group
d. 模型中的预测变量：(常量), DQ_A_T1, group, Dysfunctional_interaction(T2-T1)
e. 模型中的预测变量：(常量), DQ_A_T1, group, Dysfunctional_interaction(T2-T1), 基线月龄

残差统计[a]

	最小值	最大值	平均值	标准偏差	个案数
预测值	-40.776420593261720	72.058784484863280	39.277736648118605	25.126146426402357	61
残差	-32.976524353027344	29.505245208740234	.000000000000030	15.310798194231420	61
标准预测值	-3.186	1.305	.000	1.000	61
标准残差	-2.081	1.862	.000	.966	61

a. 因变量：DQ_A(T2-T1)

图表

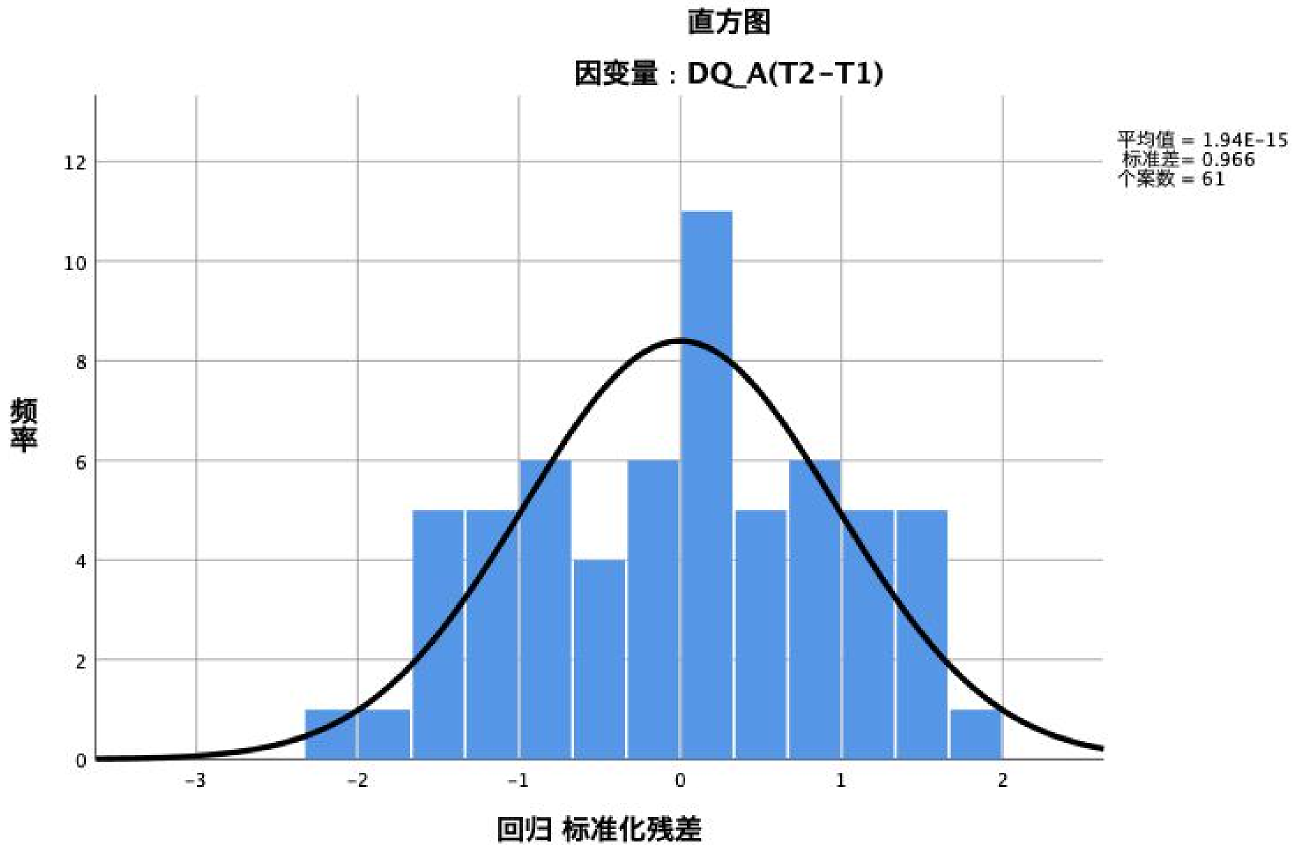

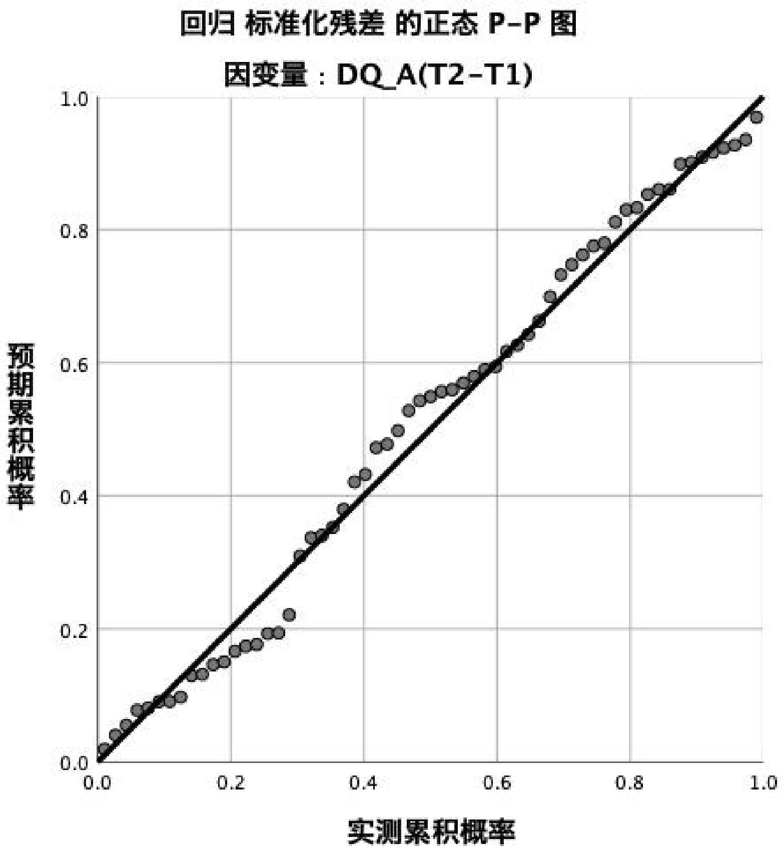


```
REGRESSION
  /MISSING LISTWISE
  /STATISTICS COEFF OUTS R ANOVA
  /CRITERIA=PIN(.05) POUT(.10)
  /NOORIGIN
  /DEPENDENT DQ_BT2T1
  /METHOD=STEPWISE group 性别 基线月龄 出生胎龄 出生体重 父亲年龄 母亲年龄 父母受教育程度 家庭年收入 PSI_totalT2T1
    Difficult_childT2T1 Dysfunctional_interactionT2T1 Paternt_distressT2T1 DQ_B_T1
  /RESIDUALS DURBIN HISTOGRAM(ZRESID) NORMPROB(ZRESID).
```

回归

备注

已创建输出		04-OCT-2022 21:09:35
注释		
输入	活动数据集	数据集9

	过滤器	<无>
	权重	<无>
	拆分文件	<无>
	工作数据文件中的行数	146
缺失值处理	对缺失的定义	将用户定义的缺失值视为缺失。
	使用的个案数	统计基于那些对于任何所用变量都不具有缺失值的个案。
语法		REGRESSION<br>/MISSING LISTWISE<br>/STATISTICS COEFF OUTS R ANOVA<br>/CRITERIA=PIN(.05) POUT(.10)<br>/NOORIGIN<br>/DEPENDENT DQ_BT2T1<br>/METHOD=STEPWISE group 性别 基线月龄 出生胎龄 出生体重 父亲年龄 母亲年龄 父母受教育程度 家庭年收入 PSI_totalT2T1<br>Difficult_childT2T1 Dysfunctional_interactionT2T1 Paternt_distressT2T1 DQ_B_T1<br>/RESIDUALS DURBIN HISTOGRAM(ZRESID) NORMPROB(ZRESID).
资源	处理程序时间	00:00:00.35
	耗用时间	00:00:01.00
	所需内存量	16352 字节
	残差图需要更多内存	432 字节

输入/除去的变量[a]

模型	输入的变量	除去的变量	方法
1	DQ_B_T1	.	步进（条件：要输入的 F 的概率 <= .050，要除去的 F 的概率 >= .100）。

2	group		.	步进（条件：要输入的 F 的概率 <= .050，要除去的 F 的概率 >= .100）。
3	基线月龄		.	步进（条件：要输入的 F 的概率 <= .050，要除去的 F 的概率 >= .100）。

a. 因变量：DQ_B(T2-T1)

模型摘要[d]

模型	R	R 方	调整后 R 方	标准估算的错误	德宾-沃森
1	.885[a]	.784	.780	15.258998084236763	
2	.900[b]	.810	.803	14.441607181093133	
3	.911[c]	.829	.820	13.807756274514588	1.765

a. 预测变量：(常量), DQ_B_T1
b. 预测变量：(常量), DQ_B_T1, group
c. 预测变量：(常量), DQ_B_T1, group, 基线月龄
d. 因变量：DQ_B(T2-T1)

ANOVA[a]

模型		平方和	自由度	均方	F	显著性
1	回归	49873.589	1	49873.589	214.200	.000[b]
	残差	13737.384	59	232.837		
	总计	63610.973	60			
2	回归	51514.492	2	25757.246	123.500	.000[c]
	残差	12096.481	58	208.560		
	总计	63610.973	60			
3	回归	52743.688	3	17581.229	92.215	.000[d]
	残差	10867.286	57	190.654		
	总计	63610.973	60			

a. 因变量：DQ_B(T2-T1)
b. 预测变量：(常量), DQ_B_T1
c. 预测变量：(常量), DQ_B_T1, group
d. 预测变量：(常量), DQ_B_T1, group, 基线月龄

系数[a]

模型		未标准化系数 B	标准错误	标准化系数 Beta	t	显著性
1	(常量)	88.564	4.252		20.830	.000
	DQ_B_T1	-1.114	.076	-.885	-14.636	.000
2	(常量)	102.143	6.295		16.226	.000
	DQ_B_T1	-1.068	.074	-.849	-14.445	.000
	group	-10.644	3.795	-.165	-2.805	.007
3	(常量)	126.328	11.267		11.212	.000
	DQ_B_T1	-1.074	.071	-.853	-15.186	.000
	group	-12.125	3.675	-.188	-3.300	.002
	基线月龄	-4.630	1.824	-.141	-2.539	.014

a. 因变量：DQ_B(T2-T1)

排除的变量[a]

模型		输入 Beta	t	显著性	偏相关	共线性统计 容差
1	group	-.165[b]	-2.805	.007	-.346	.950
	性别	.002[b]	.025	.980	.003	.998
	基线月龄	-.112[b]	-1.887	.064	-.240	.995
	出生胎龄	.048[b]	.761	.449	.099	.921
	出生体重	.051[b]	.812	.420	.106	.928
	父亲年龄	-.014[b]	-.226	.822	-.030	.978
	母亲年龄	-.033[b]	-.540	.591	-.071	.995
	父母受教育程度	-.115[b]	-1.946	.056	-.248	.995
	家庭年收入	-.004[b]	-.061	.951	-.008	.983
	PSI_total(T2-T1)	-.046[b]	-.759	.451	-.099	.999
	Difficult_child(T2-T1)	-.008[b]	-.133	.895	-.017	1.000
	Dysfunctional_interaction(T2-T1)	.045[b]	.748	.458	.098	.998
	Paternt_distress(T2-T1)	-.106[b]	-1.771	.082	-.227	.993
2	性别	.017[c]	.285	.777	.038	.990
	基线月龄	-.141[c]	-2.539	.014	-.319	.970
	出生胎龄	.031[c]	.520	.605	.069	.911
	出生体重	.011[c]	.185	.854	.024	.873
	父亲年龄	-.012[c]	-.208	.836	-.028	.978
	母亲年龄	-.036[c]	-.631	.531	-.083	.994
	父母受教育程度	-.121[c]	-2.171	.034	-.276	.994
	家庭年收入	.011[c]	.183	.856	.024	.975
	PSI_total(T2-T1)	-.026[c]	-.440	.661	-.058	.982
	Difficult_child(T2-T1)	.018[c]	.303	.763	.040	.975

	Dysfunctional_interaction(T2-T1)	.085[c]	1.468	.148	.191	.948
	Paternt_distress(T2-T1)	-.115[c]	-2.062	.044	-.263	.990
3	性别	.016[d]	.294	.769	.039	.990
	出生胎龄	.014[d]	.242	.810	.032	.898
	出生体重	-.011[d]	-.189	.850	-.025	.854
	父亲年龄	-.023[d]	-.405	.687	-.054	.973
	母亲年龄	-.051[d]	-.920	.362	-.122	.984
	父母受教育程度	-.099[d]	-1.807	.076	-.235	.961
	家庭年收入	-.010[d]	-.172	.864	-.023	.955
	PSI_total(T2-T1)	-.005[d]	-.086	.932	-.012	.960
	Difficult_child(T2-T1)	.022[d]	.392	.697	.052	.974
	Dysfunctional_interaction(T2-T1)	.090[d]	1.615	.112	.211	.947
	Paternt_distress(T2-T1)	-.089[d]	-1.605	.114	-.210	.943

a. 因变量：DQ_B(T2-T1)
b. 模型中的预测变量：(常量), DQ_B_T1
c. 模型中的预测变量：(常量), DQ_B_T1, group
d. 模型中的预测变量：(常量), DQ_B_T1, group, 基线月龄

残差统计[a]

	最小值	最大值	平均值	标准偏差	个案数
预测值	-36.892959594726560	100.312294006347670	31.445900812658344	28.115130906240797	146
残差	-44.801429748535160	36.738346099853510	-.129044116028095	16.066218496978140	146
标准预测值	-2.367	2.260	-.062	.948	146
标准残差	-3.245	2.661	-.009	1.164	146

a. 因变量：DQ_B(T2-T1)

图表

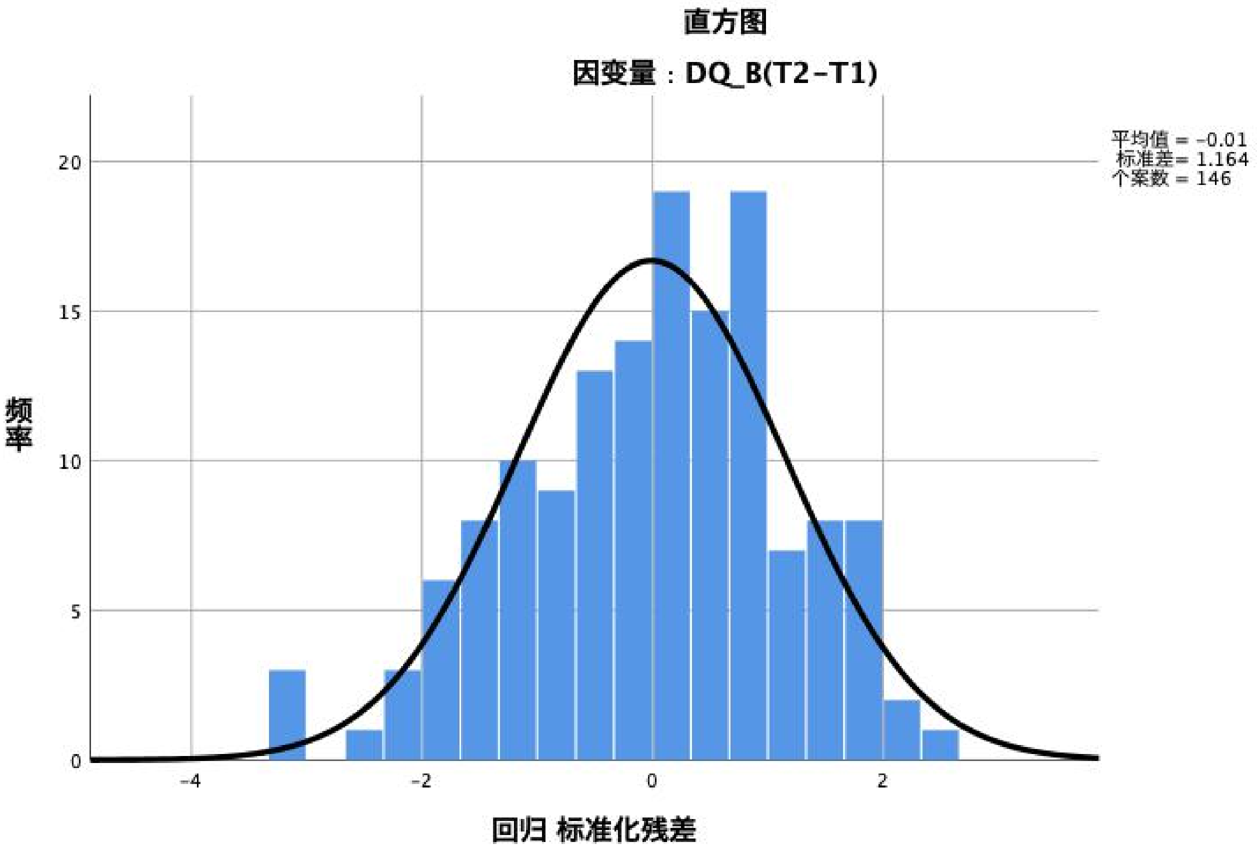

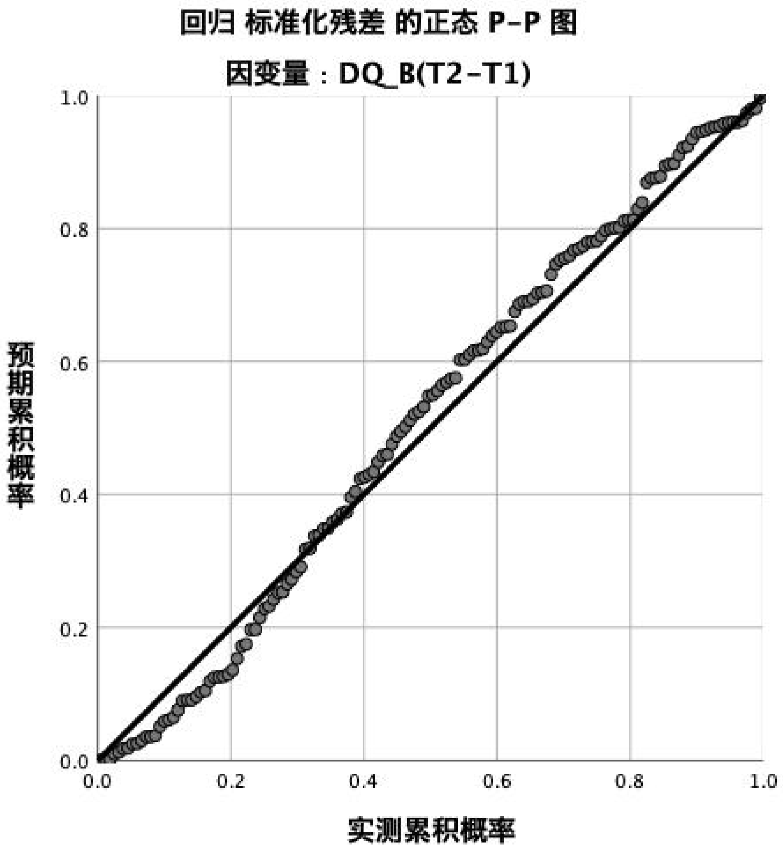


```
REGRESSION
  /MISSING LISTWISE
  /STATISTICS COEFF OUTS R ANOVA
```

```
  /CRITERIA=PIN(.05) POUT(.10)
  /NOORIGIN
  /DEPENDENT DQ_CT2T1
  /METHOD=STEPWISE group 性别 基线月龄 出生胎龄 出生体重 父亲年龄 母亲年龄 父母受教育程度 家庭年收入 PSI_totalT2T1
    Difficult_childT2T1 Dysfunctional_interactionT2T1 Paternt_distressT2T1 DQ_C_T1
  /RESIDUALS DURBIN HISTOGRAM(ZRESID) NORMPROB(ZRESID).
```

回归

备注

已创建输出		04-OCT-2022 21:10:34
注释		
输入	活动数据集	数据集9
	过滤器	<无>
	权重	<无>
	拆分文件	<无>
	工作数据文件中的行数	146
缺失值处理	对缺失的定义	将用户定义的缺失值视为缺失。
	使用的个案数	统计基于那些对于任何所用变量都不具有缺失值的个案。

语法		REGRESSION<br>/MISSING LISTWISE<br>/STATISTICS COEFF OUTS R ANOVA<br>/CRITERIA=PIN(.05) POUT(.10)<br>/NOORIGIN<br>/DEPENDENT DQ_CT2T1<br>/METHOD=STEPWISE group 性别 基线月龄 出生胎龄 出生体重 父亲年龄 母亲年龄 父母受教育程度 家庭年收入 PSI_totalT2T1 Difficult_childT2T1 Dysfunctional_interactionT2T1 Paternt_distressT2T1 DQ_C_T1<br>/RESIDUALS DURBIN HISTOGRAM(ZRESID) NORMPROB(ZRESID).
资源	处理程序时间	00:00:00.42
	耗用时间	00:00:01.00
	所需内存量	16352 字节
	残差图需要更多内存	432 字节

输入/除去的变量[a]

模型	输入的变量	除去的变量	方法
1	DQ_C_T1	.	步进（条件：要输入的 F 的概率 <= .050，要除去的 F 的概率 >= .100）。
2	基线月龄	.	步进（条件：要输入的 F 的概率 <= .050，要除去的 F 的概率 >= .100）。
3	group	.	步进（条件：要输入的 F 的概率 <= .050，要除去的 F 的概率 >= .100）。

4	Dysfunctional_interaction(T2-T1)		.	步进（条件：要输入的 F 的概率 <= .050，要除去的 F 的概率 >= .100）。

a. 因变量：DQ_C(T2-T1)

模型摘要[e]

模型	R	R 方	调整后 R 方	标准估算的错误	德宾-沃森
1	.794[a]	.631	.625	17.374788841014748	
2	.814[b]	.663	.651	16.750464892628624	
3	.834[c]	.695	.679	16.053372724482454	
4	.846[d]	.716	.696	15.642934017737026	1.650

a. 预测变量：(常量), DQ_C_T1
b. 预测变量：(常量), DQ_C_T1, 基线月龄
c. 预测变量：(常量), DQ_C_T1, 基线月龄, group
d. 预测变量：(常量), DQ_C_T1, 基线月龄, group, Dysfunctional_interaction(T2-T1)
e. 因变量：DQ_C(T2-T1)

ANOVA[a]

模型		平方和	自由度	均方	F	显著性
1	回归	30430.019	1	30430.019	100.801	.000[b]
	残差	17811.114	59	301.883		
	总计	48241.133	60			
2	回归	31967.604	2	15983.802	56.967	.000[c]
	残差	16273.528	58	280.578		
	总计	48241.133	60			
3	回归	33551.618	3	11183.873	43.397	.000[d]
	残差	14689.514	57	257.711		
	总计	48241.133	60			
4	回归	34537.855	4	8634.464	35.286	.000[e]
	残差	13703.278	56	244.701		
	总计	48241.133	60			

a. 因变量：DQ_C(T2-T1)
b. 预测变量：(常量), DQ_C_T1
c. 预测变量：(常量), DQ_C_T1, 基线月龄

d. 预测变量：(常量), DQ_C_T1, 基线月龄, group
e. 预测变量：(常量), DQ_C_T1, 基线月龄, group, Dysfunctional_interaction(T2-T1)

系数[a]

模型		未标准化系数 B	标准错误	标准化系数 Beta	t	显著性
1	(常量)	80.163	5.320		15.068	.000
	DQ_C_T1	-.979	.097	-.794	-10.040	.000
2	(常量)	102.430	10.807		9.478	.000
	DQ_C_T1	-.938	.096	-.761	-9.821	.000
	基线月龄	-5.185	2.215	-.182	-2.341	.023
3	(常量)	121.367	12.869		9.431	.000
	DQ_C_T1	-.911	.092	-.739	-9.878	.000
	基线月龄	-6.200	2.162	-.217	-2.868	.006
	group	-10.419	4.202	-.185	-2.479	.016
4	(常量)	127.462	12.902		9.879	.000
	DQ_C_T1	-.873	.092	-.709	-9.508	.000
	基线月龄	-6.510	2.112	-.228	-3.082	.003
	group	-12.396	4.212	-.220	-2.943	.005
	Dysfunctional_interaction(T2-T1)	.416	.207	.149	2.008	.050

a. 因变量：DQ_C(T2-T1)

排除的变量[a]

模型		输入 Beta	t	显著性	偏相关	共线性统计 容差
1	group	-.145[b]	-1.859	.068	-.237	.993
	性别	.011[b]	.130	.897	.017	.968
	基线月龄	-.182[b]	-2.341	.023	-.294	.967
	出生胎龄	-.020[b]	-.243	.809	-.032	.936
	出生体重	-.005[b]	-.067	.947	-.009	.965
	父亲年龄	.010[b]	.122	.903	.016	1.000
	母亲年龄	-.048[b]	-.599	.552	-.078	.966
	父母受教育程度	-.128[b]	-1.636	.107	-.210	.990
	家庭年收入	.039[b]	.490	.626	.064	.995
	PSI_total(T2-T1)	-.045[b]	-.564	.575	-.074	.978
	Difficult_child(T2-T1)	-.061[b]	-.764	.448	-.100	.975
	Dysfunctional_interaction(T2-T1)	.093[b]	1.162	.250	.151	.970
	Paternt_distress(T2-T1)	-.086[b]	-1.093	.279	-.142	1.000
2	group	-.185[c]	-2.479	.016	-.312	.957
	性别	.002[c]	.026	.979	.003	.966

	出生胎龄	-.053[c]	-.663	.510	-.087	.908
	出生体重	-.035[c]	-.448	.656	-.059	.940
	父亲年龄	-.006[c]	-.078	.938	-.010	.992
	母亲年龄	-.061[c]	-.785	.436	-.103	.961
	父母受教育程度	-.100[c]	-1.294	.201	-.169	.960
	家庭年收入	.012[c]	.154	.878	.020	.972
	PSI_total(T2-T1)	-.018[c]	-.230	.819	-.030	.956
	Difficult_child(T2-T1)	-.056[c]	-.717	.477	-.094	.974
	Dysfunctional_interaction(T2-T1)	.098[c]	1.274	.208	.166	.969
	Paternt_distress(T2-T1)	-.049[c]	-.626	.534	-.083	.951
3	性别	.013[d]	.172	.864	.023	.962
	出生胎龄	-.071[d]	-.925	.359	-.123	.901
	出生体重	-.082[d]	-1.055	.296	-.140	.893
	父亲年龄	-.001[d]	-.017	.986	-.002	.991
	母亲年龄	-.062[d]	-.823	.414	-.109	.961
	父母受教育程度	-.105[d]	-1.419	.161	-.186	.959
	家庭年收入	.030[d]	.399	.691	.053	.963
	PSI_total(T2-T1)	.016[d]	.213	.832	.028	.924
	Difficult_child(T2-T1)	-.023[d]	-.305	.762	-.041	.942
	Dysfunctional_interaction(T2-T1)	.149[d]	2.008	.050	.259	.916
	Paternt_distress(T2-T1)	-.048[d]	-.643	.523	-.086	.951
4	性别	.007[e]	.102	.919	.014	.961
	出生胎龄	-.051[e]	-.673	.504	-.090	.883
	出生体重	-.057[e]	-.736	.465	-.099	.865
	父亲年龄	.014[e]	.197	.845	.027	.980
	母亲年龄	-.053[e]	-.729	.469	-.098	.958
	父母受教育程度	-.083[e]	-1.122	.267	-.150	.932
	家庭年收入	.029[e]	.399	.692	.054	.963
	PSI_total(T2-T1)	-.131[e]	-1.379	.174	-.183	.550
	Difficult_child(T2-T1)	-.074[e]	-.956	.343	-.128	.859
	Paternt_distress(T2-T1)	-.079[e]	-1.060	.294	-.142	.917

a. 因变量：DQ_C(T2-T1)
b. 模型中的预测变量：(常量), DQ_C_T1
c. 模型中的预测变量：(常量), DQ_C_T1, 基线月龄
d. 模型中的预测变量：(常量), DQ_C_T1, 基线月龄, group
e. 模型中的预测变量：(常量), DQ_C_T1, 基线月龄, group, Dysfunctional_interaction(T2-T1)

残差统计[a]

	最小值	最大值	平均值	标准偏差	个案数
预测值	-10.876517295837402	80.767845153808600	31.643981913820040	23.992309585635600	61

残差	-42.323474884033200	34.256805419921875	.0000000000000009	15.112510017787358	61
标准预测值	-1.772	2.047	.000	1.000	61
标准残差	-2.706	2.190	.000	.966	61

a. 因变量：DQ_C(T2-T1)

图表

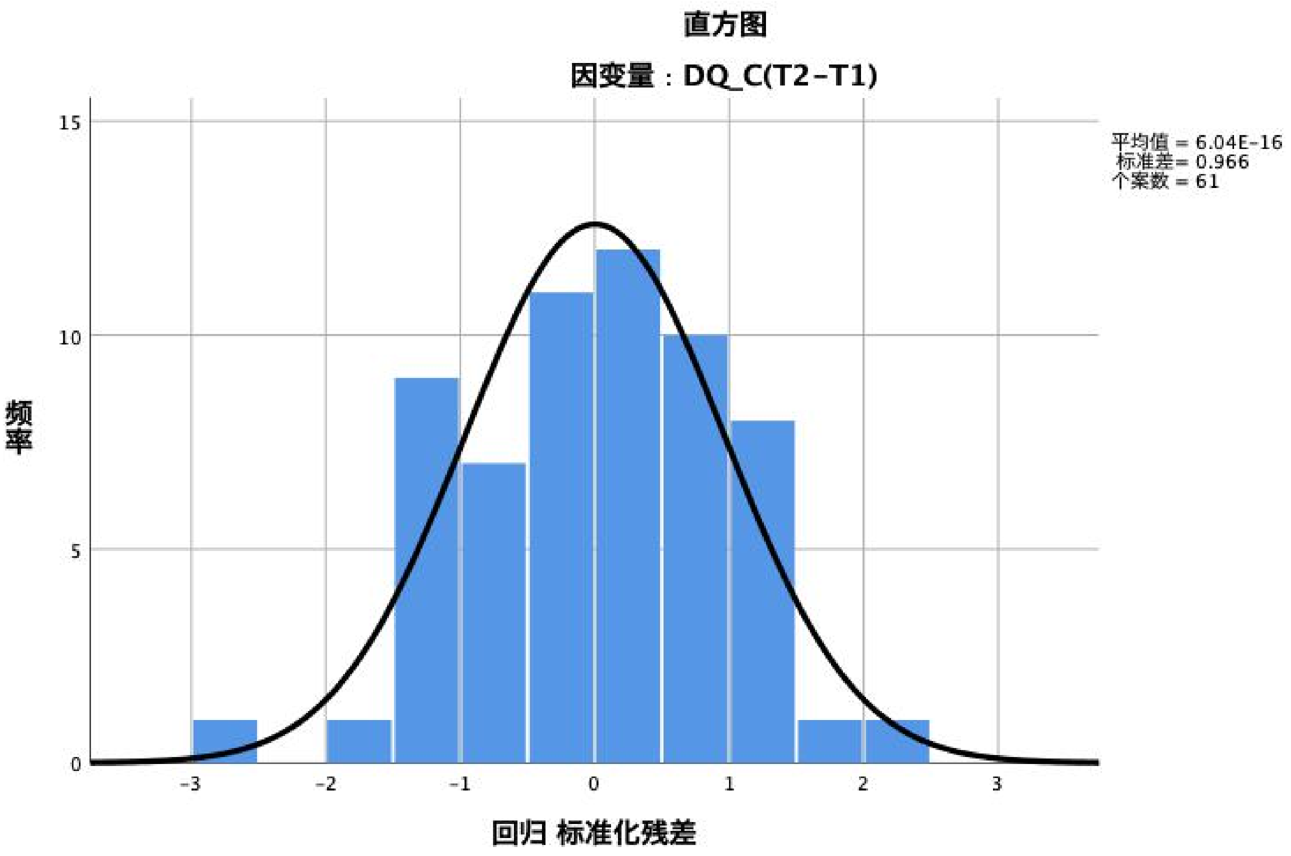

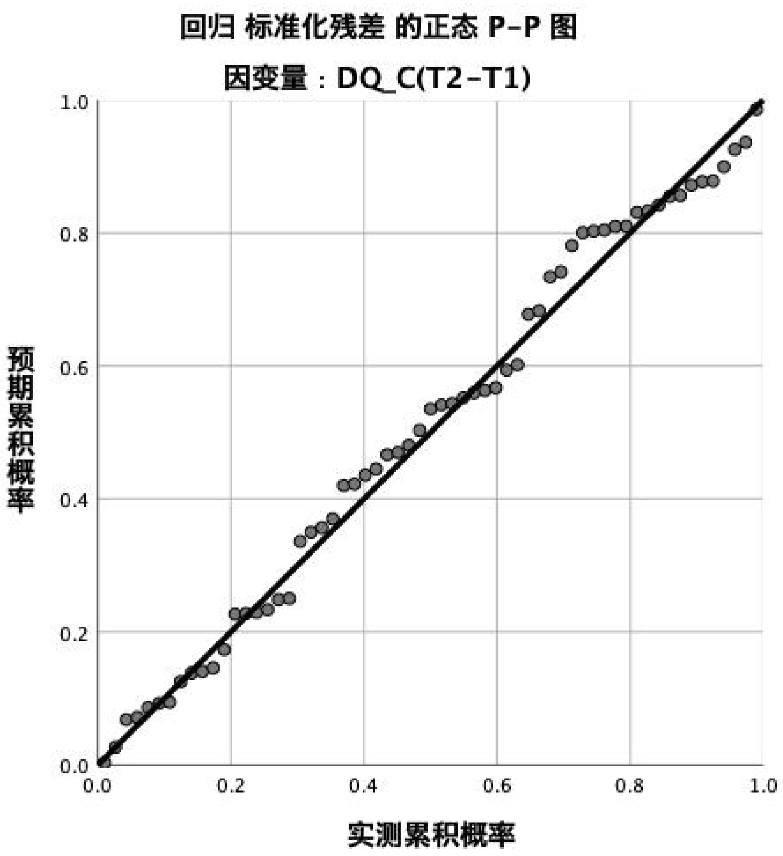


```
REGRESSION
  /MISSING LISTWISE
  /STATISTICS COEFF OUTS R ANOVA
  /CRITERIA=PIN(.05) POUT(.10)
  /NOORIGIN
  /DEPENDENT DQ_DT2T1
  /METHOD=STEPWISE group 性别 基线月龄 出生胎龄 出生体重 父亲年龄 母亲年龄 父母受教育程度 家庭年收入 PSI_totalT2T1
    Difficult_childT2T1 Dysfunctional_interactionT2T1 Paternt_distressT2T1 DQ_D_T1
  /RESIDUALS DURBIN HISTOGRAM(ZRESID) NORMPROB(ZRESID).
```

回归

备注

已创建输出		04-OCT-2022 21:11:37
注释		
输入	活动数据集	数据集9
	过滤器	<无>
	权重	<无>

	拆分文件	<无>
	工作数据文件中的行数	146
缺失值处理	对缺失的定义	将用户定义的缺失值视为缺失。
	使用的个案数	统计基于那些对于任何所用变量都不具有缺失值的个案。
语法		REGRESSION<br>/MISSING LISTWISE<br>/STATISTICS COEFF OUTS R ANOVA<br>/CRITERIA=PIN(.05) POUT(.10)<br>/NOORIGIN<br>/DEPENDENT DQ_DT2T1<br>/METHOD=STEPWISE group 性别 基线月龄 出生胎龄 出生体重 父亲年龄 母亲年龄 父母受教育程度 家庭年收入 PSI_totalT2T1 Difficult_childT2T1 Dysfunctional_interactionT2T1 Paternt_distressT2T1 DQ_D_T1<br>/RESIDUALS DURBIN HISTOGRAM(ZRESID) NORMPROB(ZRESID).
资源	处理程序时间	00:00:00.33
	耗用时间	00:00:00.00
	所需内存量	16352 字节
	残差图需要更多内存	432 字节

输入/除去的变量[a]

模型	输入的变量	除去的变量	方法
1	DQ_D_T1	.	步进（条件：要输入的 F 的概率 <= .050，要除去的 F 的概率 >= .100）。

a. 因变量：DQ_D(T2-T1)

模型摘要[b]

模型	R	R 方	调整后 R 方	标准估算的错误	德宾-沃森
1	.860[a]	.740	.735	16.475920867319715	1.922

a. 预测变量：(常量), DQ_D_T1
b. 因变量：DQ_D(T2-T1)

ANOVA[a]

模型		平方和	自由度	均方	F	显著性
1	回归	45481.523	1	45481.523	167.547	.000[b]
	残差	16015.902	59	271.456		
	总计	61497.425	60			

a. 因变量：DQ_D(T2-T1)
b. 预测变量：(常量), DQ_D_T1

系数[a]

模型		未标准化系数		标准化系数	t	显著性
		B	标准错误	Beta		
1	(常量)	83.287	4.776		17.437	.000
	DQ_D_T1	-1.005	.078	-.860	-12.944	.000

a. 因变量：DQ_D(T2-T1)

排除的变量[a]

模型		输入 Beta	t	显著性	偏相关	共线性统计 容差
1	group	-.113[b]	-1.717	.091	-.220	.988
	性别	-.008[b]	-.126	.901	-.016	.997
	基线月龄	-.129[b]	-1.975	.053	-.251	.985
	出生胎龄	.077[b]	1.165	.249	.151	.997
	出生体重	.056[b]	.836	.406	.109	.994
	父亲年龄	-.009[b]	-.126	.900	-.017	.951
	母亲年龄	-.042[b]	-.631	.531	-.083	.984
	父母受教育程度	-.102[b]	-1.478	.145	-.190	.913
	家庭年收入	.022[b]	.333	.741	.044	.990
	PSI_total(T2-T1)	.011[b]	.160	.873	.021	.966
	Difficult_child(T2-T1)	.039[b]	.582	.563	.076	1.000
	Dysfunctional_interaction(T2-T1)	.078[b]	1.165	.249	.151	.988
	Patent_distress(T2-T1)	-.072[b]	-1.047	.299	-.136	.934

a. 因变量：DQ_D(T2-T1)
b. 模型中的预测变量：(常量), DQ_D_T1

残差统计[a]

	最小值	最大值	平均值	标准偏差	个案数
预测值	-78.866294860839840	83.287010192871100	25.942301676365220	28.044029942074140	146
残差	-51.388469696044920	25.450132369995117	-.404767572804072	15.469546948389919	146
标准预测值	-3.875	2.015	-.068	1.019	146
标准残差	-3.119	1.545	-.025	.939	146

a. 因变量：DQ_D(T2-T1)

图表

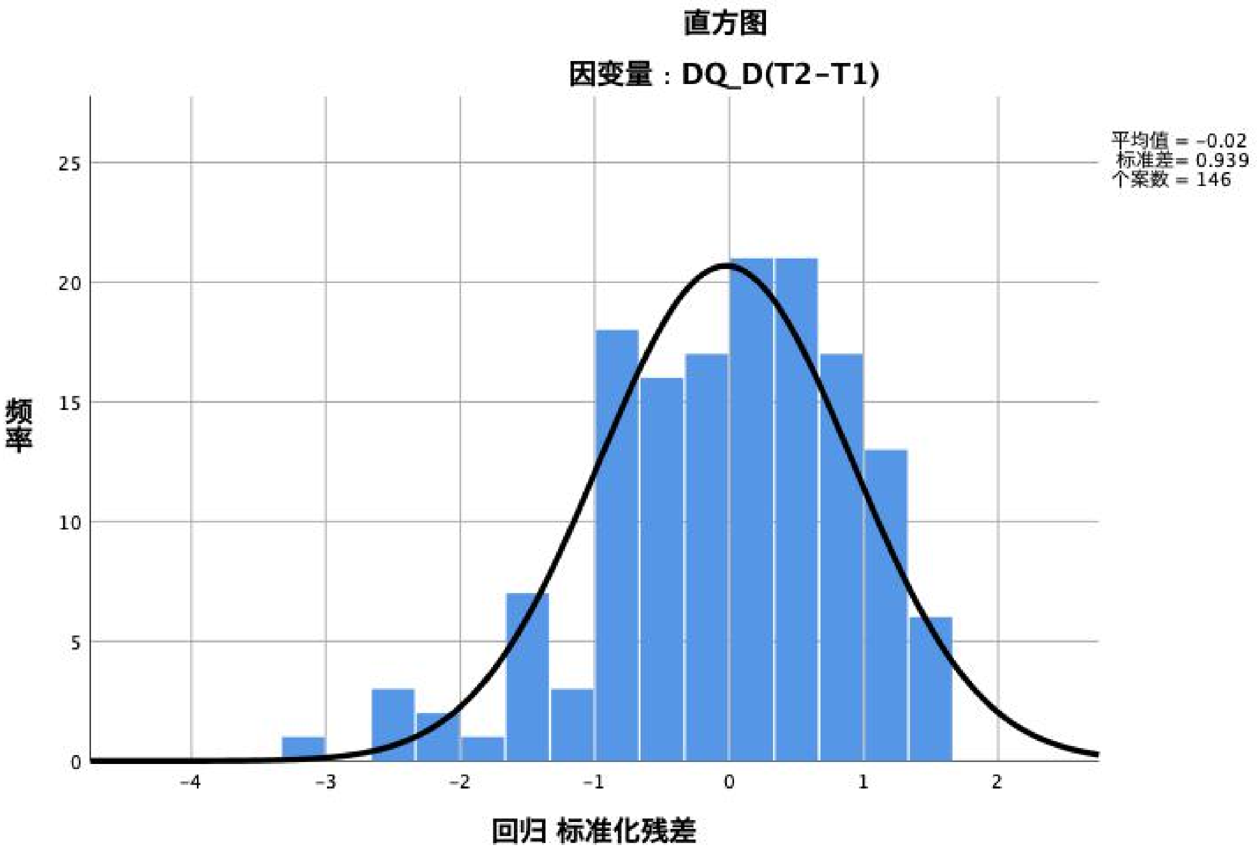

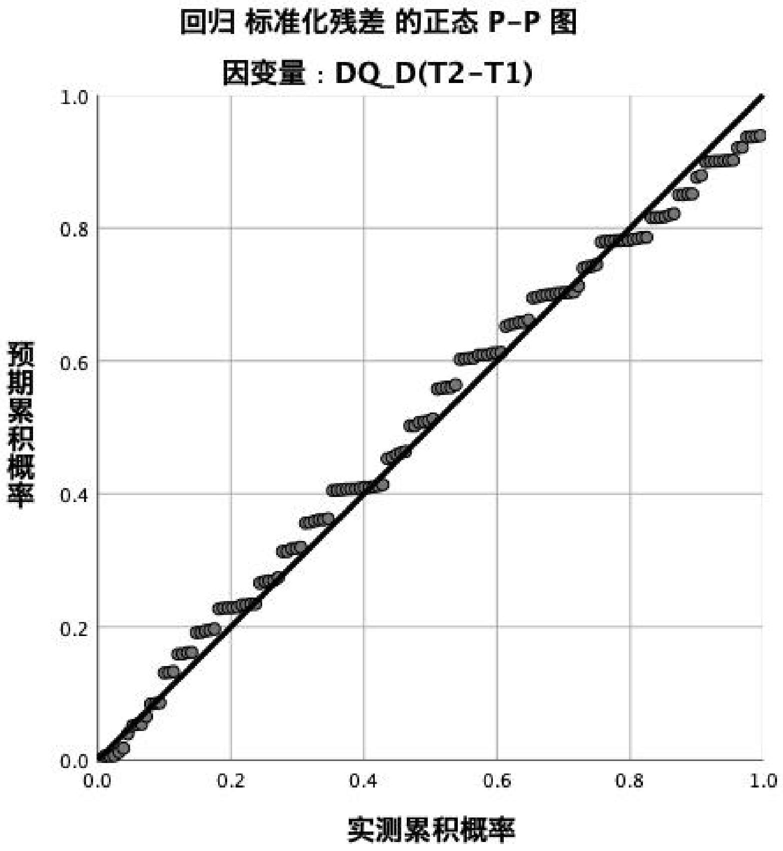


```
REGRESSION
  /MISSING LISTWISE
  /STATISTICS COEFF OUTS R ANOVA
  /CRITERIA=PIN(.05) POUT(.10)
  /NOORIGIN
  /DEPENDENT DQ_ET2T1
  /METHOD=STEPWISE group 性别 基线月龄 出生胎龄 出生体重 父亲年龄 母亲年龄 父母受教育程度 家庭年收入 PSI_totalT2T1
    Difficult_childT2T1 Dysfunctional_interactionT2T1 Paternt_distressT2T1 DQ_E_T1
  /RESIDUALS DURBIN HISTOGRAM(ZRESID) NORMPROB(ZRESID).
```

回归

备注

已创建输出		04-OCT-2022 21:12:25
注释		
输入	活动数据集	数据集9
	过滤器	<无>
	权重	<无>

	拆分文件	<无>
	工作数据文件中的行数	146
缺失值处理	对缺失的定义	将用户定义的缺失值视为缺失。
	使用的个案数	统计基于那些对于任何所用变量都不具有缺失值的个案。
语法		REGRESSION /MISSING LISTWISE /STATISTICS COEFF OUTS R ANOVA /CRITERIA=PIN(.05) POUT(.10) /NOORIGIN /DEPENDENT DQ_ET2T1 /METHOD=STEPWISE group 性别 基线月龄 出生胎龄 出生体重 父亲年龄 母亲年龄 父母受教育程度 家庭年收入 PSI_totalT2T1 Difficult_childT2T1 Dysfunctional_interactionT2T1 Paternt_distressT2T1 DQ_E_T1 /RESIDUALS DURBIN HISTOGRAM(ZRESID) NORMPROB(ZRESID).
资源	处理程序时间	00:00:00.31
	耗用时间	00:00:00.00
	所需内存量	16352 字节
	残差图需要更多内存	432 字节

输入/除去的变量[a]

模型	输入的变量	除去的变量	方法
1	DQ_E_T1	.	步进（条件：要输入的 F 的概率 <= .050，要除去的 F 的概率 >= .100）。
2	group	.	步进（条件：要输入的 F 的概率 <= .050，要除去的 F 的概率 >= .100）。

3	Dysfunctional_interaction(T2-T1)	.	步进（条件：要输入的 F 的概率 <= .050，要除去的 F 的概率 >= .100）。
4	基线月龄	.	步进（条件：要输入的 F 的概率 <= .050，要除去的 F 的概率 >= .100）。

a. 因变量：DQ_E(T2-T1)

模型摘要[e]

模型	R	R 方	调整后 R 方	标准估算的错误	德宾-沃森
1	.846[a]	.715	.710	17.225396668630210	
2	.869[b]	.754	.746	16.127486045636807	
3	.881[c]	.776	.765	15.527200719498476	
4	.892[d]	.796	.782	14.952338928864190	2.277

a. 预测变量：(常量), DQ_E_T1
b. 预测变量：(常量), DQ_E_T1, group
c. 预测变量：(常量), DQ_E_T1, group, Dysfunctional_interaction(T2-T1)
d. 预测变量 :(常量), DQ_E_T1, group, Dysfunctional_interaction(T2-T1), 基线月龄
e. 因变量：DQ_E(T2-T1)

ANOVA[a]

模型		平方和	自由度	均方	F	显著性
1	回归	43929.051	1	43929.051	148.052	.000[b]
	残差	17506.143	59	296.714		
	总计	61435.194	60			
2	回归	46349.637	2	23174.819	89.101	.000[c]
	残差	15085.557	58	260.096		
	总计	61435.194	60			
3	回归	47692.838	3	15897.613	65.939	.000[d]
	残差	13742.356	57	241.094		
	总计	61435.194	60			
4	回归	48915.137	4	12228.784	54.697	.000[e]
	残差	12520.057	56	223.572		

总计	61435.194	60			

a. 因变量：DQ_E(T2-T1)
b. 预测变量：(常量), DQ_E_T1
c. 预测变量：(常量), DQ_E_T1, group
d. 预测变量：(常量), DQ_E_T1, group, Dysfunctional_interaction(T2-T1)
e. 预测变量：(常量), DQ_E_T1, group, Dysfunctional_interaction(T2-T1), 基线月龄

系数[a]

模型		未标准化系数 B	标准错误	标准化系数 Beta	t	显著性
1	(常量)	79.043	5.015		15.762	.000
	DQ_E_T1	-.945	.078	-.846	-12.168	.000
2	(常量)	97.137	7.565		12.841	.000
	DQ_E_T1	-.933	.073	-.834	-12.801	.000
	group	-12.621	4.137	-.199	-3.051	.003
3	(常量)	103.818	7.814		13.286	.000
	DQ_E_T1	-.926	.070	-.829	-13.196	.000
	group	-14.613	4.072	-.230	-3.589	.001
	Dysfunctional_interaction(T2-T1)	.476	.201	.151	2.360	.022
4	(常量)	127.630	12.662		10.080	.000
	DQ_E_T1	-.920	.068	-.823	-13.594	.000
	group	-16.246	3.983	-.256	-4.079	.000
	Dysfunctional_interaction(T2-T1)	.491	.194	.156	2.528	.014
	基线月龄	-4.621	1.976	-.143	-2.338	.023

a. 因变量：DQ_E(T2-T1)

排除的变量[a]

模型		输入 Beta	t	显著性	偏相关	共线性统计 容差
1	group	-.199[b]	-3.051	.003	-.372	.997
	性别	-.016[b]	-.223	.824	-.029	.994
	基线月龄	-.100[b]	-1.450	.152	-.187	.999
	出生胎龄	.054[b]	.774	.442	.101	.995
	出生体重	.050[b]	.722	.473	.094	.999
	父亲年龄	-.016[b]	-.225	.823	-.030	.993
	母亲年龄	-.050[b]	-.715	.477	-.093	.986
	父母受教育程度	-.048[b]	-.668	.507	-.087	.960
	家庭年收入	-.023[b]	-.317	.752	-.042	.954
	PSI_total(T2-T1)	.046[b]	.652	.517	.085	.976

	Difficult_child(T2-T1)	.051[b]	.719	.475	.094	.984
	Dysfunctional_interaction(T2-T1)	.104[b]	1.505	.138	.194	.999
	Paternt_distress(T2-T1)	-.035[b]	-.488	.627	-.064	.979
2	性别	.001[c]	.020	.984	.003	.987
	基线月龄	-.138[c]	-2.156	.035	-.275	.969
	出生胎龄	.047[c]	.715	.478	.094	.994
	出生体重	.017[c]	.260	.796	.034	.971
	父亲年龄	-.008[c]	-.123	.903	-.016	.992
	母亲年龄	-.053[c]	-.799	.428	-.105	.986
	父母受教育程度	-.060[c]	-.903	.370	-.119	.957
	家庭年收入	-.001[c]	-.020	.984	-.003	.944
	PSI_total(T2-T1)	.077[c]	1.156	.252	.151	.956
	Difficult_child(T2-T1)	.086[c]	1.296	.200	.169	.958
	Dysfunctional_interaction(T2-T1)	.151[c]	2.360	.022	.298	.956
	Paternt_distress(T2-T1)	-.041[c]	-.615	.541	-.081	.978
3	性别	.002[d]	.029	.977	.004	.987
	基线月龄	-.143[d]	-2.338	.023	-.298	.968
	出生胎龄	.078[d]	1.223	.226	.161	.958
	出生体重	.053[d]	.806	.424	.107	.925
	父亲年龄	.008[d]	.131	.896	.017	.980
	母亲年龄	-.050[d]	-.784	.437	-.104	.986
	父母受教育程度	-.036[d]	-.549	.585	-.073	.930
	家庭年收入	-.004[d]	-.068	.946	-.009	.943
	PSI_total(T2-T1)	-.037[d]	-.441	.661	-.059	.552
	Difficult_child(T2-T1)	.041[d]	.606	.547	.081	.858
	Paternt_distress(T2-T1)	-.072[d]	-1.118	.268	-.148	.942
4	性别	.002[e]	.040	.968	.005	.986
	出生胎龄	.061[e]	.982	.331	.131	.943
	出生体重	.031[e]	.486	.629	.065	.903
	父亲年龄	-.003[e]	-.045	.964	-.006	.974
	母亲年龄	-.066[e]	-1.074	.287	-.143	.974
	父母受教育程度	-.009[e]	-.139	.890	-.019	.898
	家庭年收入	-.028[e]	-.446	.658	-.060	.919
	PSI_total(T2-T1)	-.004[e]	-.043	.966	-.006	.534
	Difficult_child(T2-T1)	.045[e]	.693	.491	.093	.857
	Paternt_distress(T2-T1)	-.042[e]	-.657	.514	-.088	.897

a. 因变量：DQ_E(T2-T1)
b. 模型中的预测变量：(常量), DQ_E_T1
c. 模型中的预测变量：(常量), DQ_E_T1, group
d. 模型中的预测变量：(常量), DQ_E_T1, group, Dysfunctional_interaction(T2-T1)
e. 模型中的预测变量：(常量), DQ_E_T1, group, Dysfunctional_interaction(T2-T1), 基线月龄

残差统计[a]

	最小值	最大值	平均值	标准偏差	个案数
预测值	-48.352260589599610	77.283729553222660	24.240603138682314	28.552623167438680	61
残差	-31.174627304077150	34.033870697021484	-.000000000000009	14.445331776992354	61
标准预测值	-2.542	1.858	.000	1.000	61
标准残差	-2.085	2.276	.000	.966	61

a. 因变量：DQ_E(T2-T1)

图表

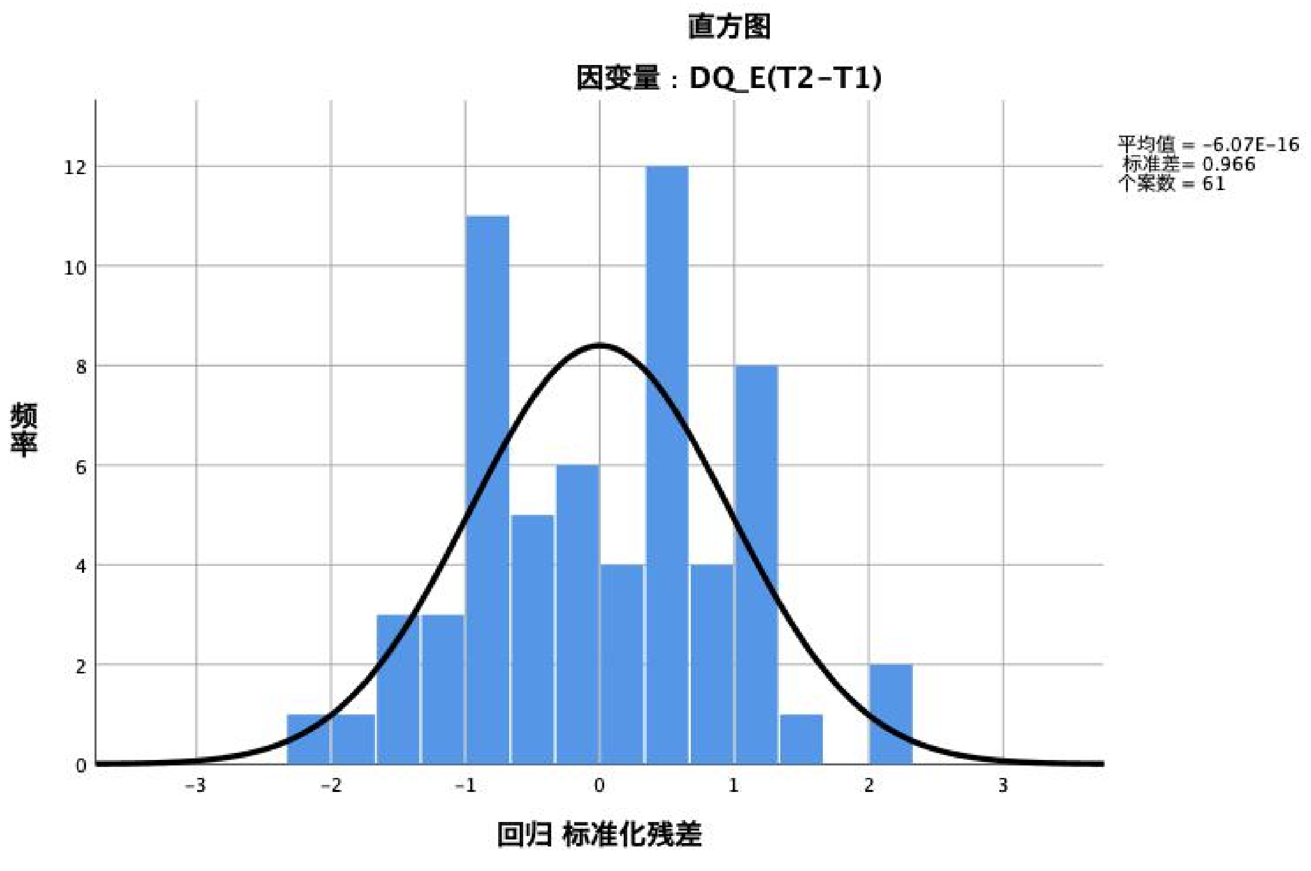

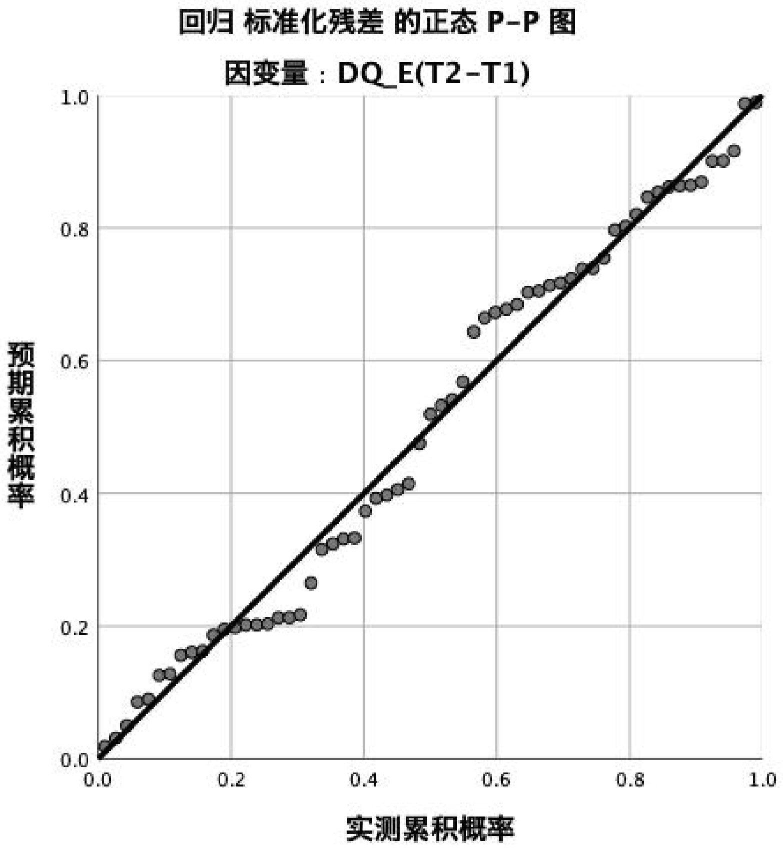


```
REGRESSION
  /MISSING LISTWISE
  /STATISTICS COEFF OUTS R ANOVA
  /CRITERIA=PIN(.05) POUT(.10)
  /NOORIGIN
  /DEPENDENT GQT2T1
  /METHOD=STEPWISE group 性别 基线月龄 出生胎龄 出生体重 父亲年龄 母亲年龄 父母受教育程度 家庭年收入 PSI_totalT2T1
    Difficult_childT2T1 Dysfunctional_interactionT2T1 Paternt_distressT2T1 GQ_T1
  /RESIDUALS DURBIN HISTOGRAM(ZRESID) NORMPROB(ZRESID).
```

回归

备注

已创建输出		04-OCT-2022 21:13:03
注释		
输入	活动数据集	数据集9
	过滤器	<无>
	权重	<无>

	拆分文件	<无>
	工作数据文件中的行数	146
缺失值处理	对缺失的定义	将用户定义的缺失值视为缺失。
	使用的个案数	统计基于那些对于任何所用变量都不具有缺失值的个案。
语法		REGRESSION<br>/MISSING LISTWISE<br>/STATISTICS COEFF OUTS R ANOVA<br>/CRITERIA=PIN(.05) POUT(.10)<br>/NOORIGIN<br>/DEPENDENT GQT2T1<br>/METHOD=STEPWISE group 性别 基线月龄 出生胎龄 出生体重 父亲年龄 母亲年龄 父母受教育程度 家庭年收入 PSI_totalT2T1 Difficult_childT2T1 Dysfunctional_interactionT2T1 Paternt_distressT2T1 GQ_T1<br>/RESIDUALS DURBIN HISTOGRAM(ZRESID) NORMPROB(ZRESID).
资源	处理程序时间	00:00:00.29
	耗用时间	00:00:00.00
	所需内存量	16352 字节
	残差图需要更多内存	432 字节

输入/除去的变量[a]

模型	输入的变量	除去的变量	方法
1	GQ_T1	.	步进（条件：要输入的 F 的概率 <= .050，要除去的 F 的概率 >= .100）。
2	group	.	步进（条件：要输入的 F 的概率 <= .050，要除去的 F 的概率 >= .100）。

3	基线月龄	.	步进（条件：要输入的 F 的概率 <= .050，要除去的 F 的概率 >= .100）。
4	Dysfunctional_interaction(T2-T1)	.	步进（条件：要输入的 F 的概率 <= .050，要除去的 F 的概率 >= .100）。

a. 因变量：GQ(T2-T1)

模型摘要[e]

模型	R	R 方	调整后 R 方	标准估算的错误	德宾-沃森
1	.772[a]	.595	.588	15.352348559142783	
2	.798[b]	.637	.625	14.662700453467286	
3	.821[c]	.675	.657	14.005999290748058	
4	.837[d]	.700	.679	13.556380452735233	1.756

a. 预测变量：(常量), GQ_T1
b. 预测变量：(常量), GQ_T1, group
c. 预测变量：(常量), GQ_T1, group, 基线月龄
d. 预测变量：(常量), GQ_T1, group, 基线月龄, Dysfunctional_interaction(T2-T1)
e. 因变量：GQ(T2-T1)

ANOVA[a]

模型		平方和	自由度	均方	F	显著性
1	回归	20449.330	1	20449.330	86.762	.000[b]
	残差	13905.982	59	235.695		
	总计	34355.311	60			
2	回归	21885.614	2	10942.807	50.898	.000[c]
	残差	12469.698	58	214.995		
	总计	34355.311	60			
3	回归	23173.734	3	7724.578	39.377	.000[d]
	残差	11181.577	57	196.168		
	总计	34355.311	60			
4	回归	24063.886	4	6015.972	32.735	.000[e]
	残差	10291.425	56	183.775		
	总计	34355.311	60			

a. 因变量：GQ(T2-T1)
b. 预测变量：(常量), GQ_T1
c. 预测变量：(常量), GQ_T1, group
d. 预测变量：(常量), GQ_T1, group, 基线月龄
e. 预测变量：(常量), GQ_T1, group, 基线月龄, Dysfunctional_interaction(T2-T1)

系数[a]

模型		未标准化系数 B	标准错误	标准化系数 Beta	t	显著性
1	(常量)	84.702	6.065		13.965	.000
	GQ_T1	-1.044	.112	-.772	-9.315	.000
2	(常量)	97.176	7.540		12.888	.000
	GQ_T1	-1.001	.108	-.740	-9.248	.000
	group	-9.820	3.799	-.207	-2.585	.012
3	(常量)	120.742	11.681		10.337	.000
	GQ_T1	-.976	.104	-.722	-9.397	.000
	group	-11.548	3.691	-.243	-3.128	.003
	基线月龄	-4.758	1.857	-.197	-2.563	.013
4	(常量)	126.870	11.644		10.896	.000
	GQ_T1	-.969	.101	-.717	-9.637	.000
	group	-13.235	3.654	-.279	-3.622	.001
	基线月龄	-4.894	1.798	-.203	-2.722	.009
	Dysfunctional_interaction(T2-T1)	.387	.176	.165	2.201	.032

a. 因变量：GQ(T2-T1)

排除的变量[a]

模型		输入 Beta	t	显著性	偏相关	共线性统计 容差
1	group	-.207[b]	-2.585	.012	-.321	.977
	性别	-.019[b]	-.222	.825	-.029	.995
	基线月龄	-.153[b]	-1.887	.064	-.241	.996
	出生胎龄	.040[b]	.470	.640	.062	.954
	出生体重	.040[b]	.480	.633	.063	.978
	父亲年龄	-.006[b]	-.067	.947	-.009	.970
	母亲年龄	-.054[b]	-.651	.517	-.085	.994
	父母受教育程度	-.117[b]	-1.413	.163	-.182	.979
	家庭年收入	.001[b]	.010	.992	.001	.975
	PSI_total(T2-T1)	-.011[b]	-.137	.892	-.018	1.000
	Difficult_child(T2-T1)	.015[b]	.178	.859	.023	.991

	Dysfunctional_interaction(T2-T1)	.109[b]	1.318	.193	.171	1.000
	Paternt_distress(T2-T1)	-.108[b]	-1.299	.199	-.168	.987
2	性别	.000[c]	.005	.996	.001	.987
	基线月龄	-.197[c]	-2.563	.013	-.321	.962
	出生胎龄	.026[c]	.319	.751	.042	.950
	出生体重	.000[c]	.004	.997	.001	.941
	父亲年龄	-.002[c]	-.025	.980	-.003	.970
	母亲年龄	-.058[c]	-.725	.471	-.096	.994
	父母受教育程度	-.133[c]	-1.684	.098	-.218	.974
	家庭年收入	.020[c]	.251	.803	.033	.966
	PSI_total(T2-T1)	.016[c]	.197	.844	.026	.982
	Difficult_child(T2-T1)	.052[c]	.640	.525	.084	.962
	Dysfunctional_interaction(T2-T1)	.158[c]	1.999	.050	.256	.957
	Paternt_distress(T2-T1)	-.120[c]	-1.519	.134	-.197	.983
3	性别	.002[d]	.026	.979	.004	.987
	出生胎龄	-.004[d]	-.048	.962	-.006	.929
	出生体重	-.036[d]	-.446	.657	-.059	.913
	父亲年龄	-.021[d]	-.270	.788	-.036	.961
	母亲年龄	-.080[d]	-1.053	.297	-.139	.982
	父母受教育程度	-.102[d]	-1.327	.190	-.175	.945
	家庭年收入	-.012[d]	-.152	.880	-.020	.941
	PSI_total(T2-T1)	.046[d]	.594	.555	.079	.960
	Difficult_child(T2-T1)	.060[d]	.779	.439	.104	.960
	Dysfunctional_interaction(T2-T1)	.165[d]	2.201	.032	.282	.956
	Paternt_distress(T2-T1)	-.083[d]	-1.072	.288	-.142	.942
4	性别	.002[e]	.034	.973	.005	.987
	出生胎龄	.029[e]	.369	.714	.050	.896
	出生体重	.001[e]	.011	.991	.001	.870
	父亲年龄	-.003[e]	-.046	.964	-.006	.950
	母亲年龄	-.077[e]	-1.047	.299	-.140	.981
	父母受教育程度	-.075[e]	-.985	.329	-.132	.915
	家庭年收入	-.016[e]	-.205	.838	-.028	.940
	PSI_total(T2-T1)	-.105[e]	-1.072	.288	-.143	.554
	Difficult_child(T2-T1)	.008[e]	.101	.920	.014	.860
	Paternt_distress(T2-T1)	-.121[e]	-1.593	.117	-.210	.905

a. 因变量：GQ(T2-T1)
b. 模型中的预测变量：(常量), GQ_T1
c. 模型中的预测变量：(常量), GQ_T1, group
d. 模型中的预测变量：(常量), GQ_T1, group, 基线月龄
e. 模型中的预测变量：(常量), GQ_T1, group, 基线月龄, Dysfunctional_interaction(T2-T1)

残差统计[a]

	最小值	最大值	平均值	标准偏差	个案数
预测值	-12.785430908203123	77.448554992675780	31.255003319507670	20.026601497883362	61
残差	-30.788562774658203	24.397701263427734	.000000000000016	13.096707763684284	61
标准预测值	-2.199	2.307	.000	1.000	61
标准残差	-2.271	1.800	.000	.966	61

a. 因变量：GQ(T2-T1)

图表

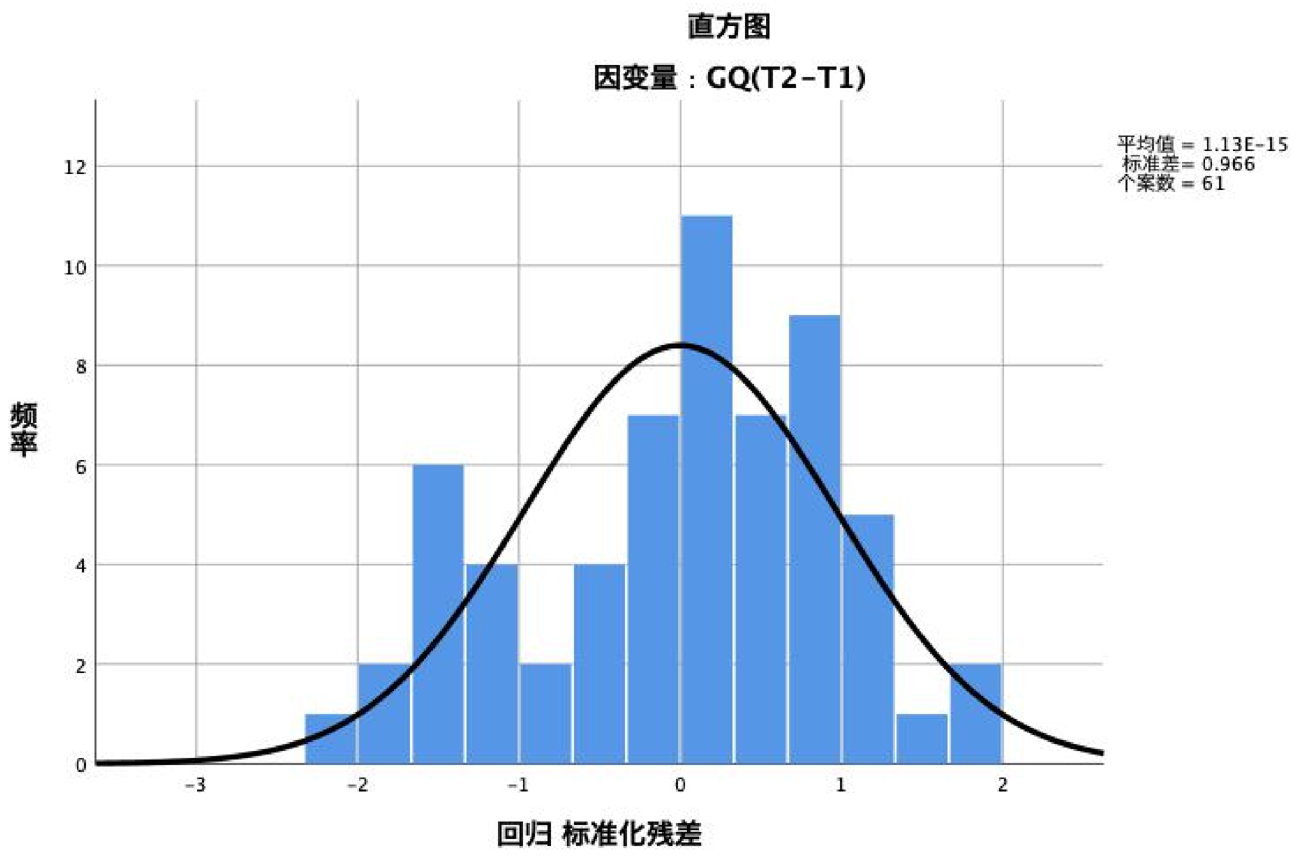

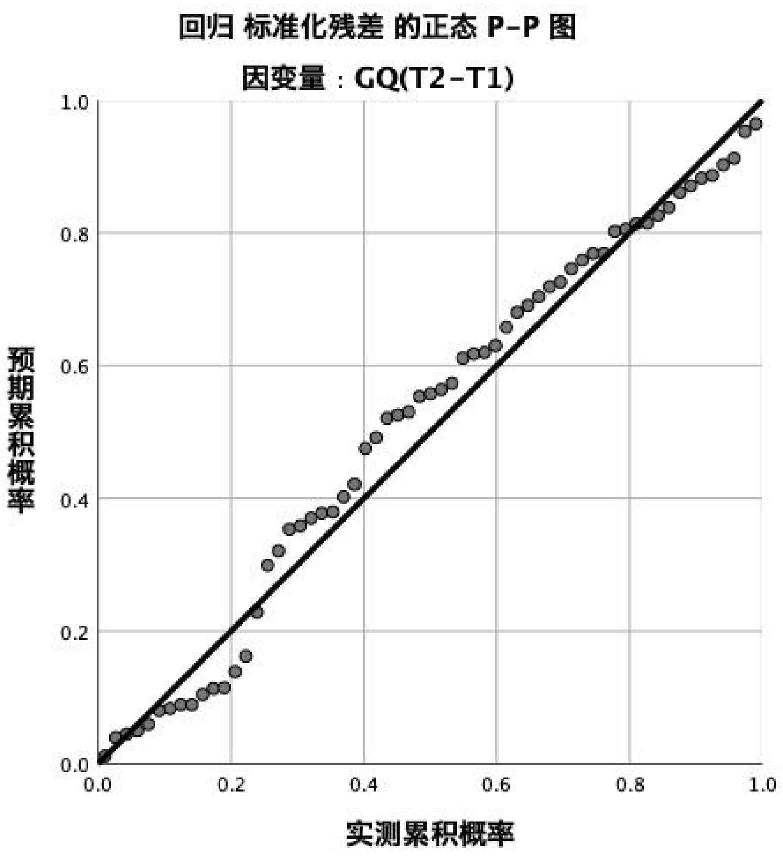
回归 标准化残差 的正态 P-P 图
因变量：GQ(T2-T1)
预期累积概率
实测累积概率
0.0
0.2
0.4
0.6
0.8
1.0
